# Shifting social-ecological fire regimes explain increasing structure loss from Western wildfires

**DOI:** 10.1093/pnasnexus/pgad005

**Published:** 2023-02-01

**Authors:** Philip E Higuera, Maxwell C Cook, Jennifer K Balch, E Natasha Stavros, Adam L Mahood, Lise A St. Denis

**Affiliations:** Department of Ecosystem and Conservation Sciences, University of Montana, 32 Campus Drive, Missoula, MT 59812, USA; Earth Lab, Cooperative Institute for Research in Environmental Sciences, University of Colorado Boulder, 4001 Discovery Drive, Suite S348, 611 UCB, Boulder, CO 80303, USA; Department of Geography, University of Colorado Boulder, Guggenheim 110, 260 UCB, Boulder, CO 80309, USA; Earth Lab, Cooperative Institute for Research in Environmental Sciences, University of Colorado Boulder, 4001 Discovery Drive, Suite S348, 611 UCB, Boulder, CO 80303, USA; Department of Geography, University of Colorado Boulder, Guggenheim 110, 260 UCB, Boulder, CO 80309, USA; Earth Lab, Cooperative Institute for Research in Environmental Sciences, University of Colorado Boulder, 4001 Discovery Drive, Suite S348, 611 UCB, Boulder, CO 80303, USA; Earth Lab, Cooperative Institute for Research in Environmental Sciences, University of Colorado Boulder, 4001 Discovery Drive, Suite S348, 611 UCB, Boulder, CO 80303, USA; Water Resources, Agriculture Research Service, United States Department of Agriculture, 2150 Centre Avenue, Building D, Fort Collins, CO 80526, USA; Earth Lab, Cooperative Institute for Research in Environmental Sciences, University of Colorado Boulder, 4001 Discovery Drive, Suite S348, 611 UCB, Boulder, CO 80303, USA

**Keywords:** anthropogenic wildfires, fire disasters, western United States, human impacts, wildfire crisis

## Abstract

Structure loss is an acute, costly impact of the wildfire crisis in the western conterminous United States (“West”), motivating the need to understand recent trends and causes. We document a 246% rise in West-wide structure loss from wildfires between 1999–2009 and 2010–2020, driven strongly by events in 2017, 2018, and 2020. Increased structure loss was not due to increased area burned alone. Wildfires became significantly more destructive, with a 160% higher structure-loss rate (loss/kha burned) over the past decade. Structure loss was driven primarily by wildfires from unplanned human-related ignitions (e.g. backyard burning, power lines, etc.), which accounted for 76% of all structure loss and resulted in 10 times more structures destroyed per unit area burned compared with lightning-ignited fires. Annual structure loss was well explained by area burned from human-related ignitions, while decadal structure loss was explained by state-level structure abundance in flammable vegetation. Both predictors increased over recent decades and likely interacted with increased fuel aridity to drive structure-loss trends. While states are diverse in patterns and trends, nearly all experienced more burning from human-related ignitions and/or higher structure-loss rates, particularly California, Washington, and Oregon. Our findings highlight how fire regimes—characteristics of fire over space and time—are fundamentally social-ecological phenomena. By resolving the diversity of Western fire regimes, our work informs regionally appropriate mitigation and adaptation strategies. With millions of structures with high fire risk, reducing human-related ignitions and rethinking how we build are critical for preventing future wildfire disasters.

**Significance Statement**

Wildfires in the western United States (“West”) have caused significant negative human impacts in recent years, in part by destroying homes and other structures. We summarized recent trends in wildfire-caused structure loss across the West and found that structure loss more than tripled between 1999–2010 and 2010–2020, and not just because of more burning. Rather, the average number of structures destroyed per unit area burned increased by 160%. Wildfires from human-related ignitions, more common in states with more structures in flammable vegetation, played a far outsized role in causing structure loss compared with lightning-ignited wildfires. Addressing the wildfire crisis requires minimizing unplanned ignitions, carefully considering if and how we build among flammable vegetation, and treating wildfires as coupled social-ecological phenomena.

## Introduction

Increasing global fire danger over the past four decades (1–3) has officially reached crisis levels (4, 5). Climate change (6), land use (7–9), and other accumulated impacts of industrialization are compounding to turn more wildfires into human disasters (3). While fire is a longstanding and fundamental ecological process in most terrestrial ecosystems (10), evidence is accumulating that fire activity is exceeding the range of variability that has characterized some ecosystems for millennia (11, 12).

In the western conterminous United States (“West,” "Western"), area burned by wildfires has doubled over the past four decades (13), in part enabled by increased fuel aridity due to anthropogenic climate change (14–17). Drier fuels ignite more easily. Once started, fires spread faster, burn at higher intensities, and produce more extreme fire behavior that limits fire control. Past land uses and policies that limit indigenous fire stewardship and focus on fire suppression have increased flammable fuels and compounded climate-driven fire risk, particularly in ecosystems that historically burned frequently in the past (18, 19).

Increased burning is also happening in the context of a West-wide expansion and densification of structures in flammable vegetation, increasing their exposure to wildfires (8, 20) and the likelihood of unplanned or accidental ignitions (21). Unplanned ignitions from human sources—*hereafter* “*human-related ignitions*”*—*include backyard burning, downed power lines, escaped campfires, etc., and are a well-recognized component of contemporary fire activity across the United States (22, 23). Human-related ignitions expand the fire season beyond periods when lightning ignition is common, and they tend to be concentrated near homes, making them more costly and destructive to valued human resources compared with lightning-ignited fires (21).

**Fig. 1.**
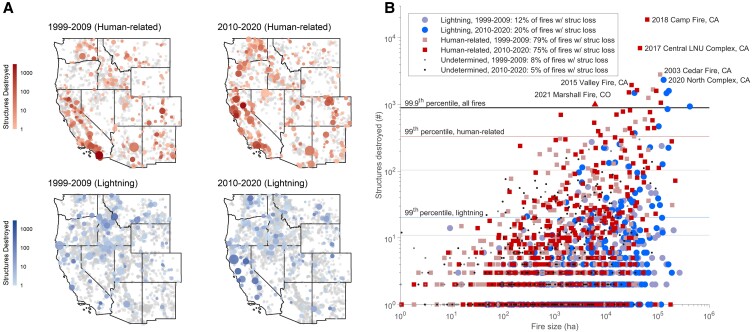
Area burned and structure loss from wildfires in the western United States, 1999–2020. (A) Geographic distribution of wildfires, by ignition source (rows) and time period (columns). Circle size represents fire size (log scale), color represents structure loss (log scale), with gray indicating no associated structure loss. (B) Total structure loss as a function of fire size for the 1,825 fires from A (12% of total) with associated structure loss, stratified by ignition source and time period. Extremes are defined by 99th percentile for each ignition type and decade, and by the 99.9th percentile for the overall data set. Legend includes the percent of total fires in each category with structure loss. Note: the 2021 Marshall Fire (triangle) is included as an example but is not part of the 1999–2020 data set analyzed; although it occurred without regional lightning activity, at time of publication it has a yet-undetermined ignition source.

These interacting drivers of wildfires and fire disasters have peaked in recent years in the West, resulting in a surge of exceptionally destructive wildfires (Fig. 1) and catalyzing policy and management efforts aimed at more safely living in an increasingly flammable region (e.g. 5). These efforts highlight an urgent need to understand the degree to which changes in climate, human development patterns, and human-related ignitions are driving trends in area burned and the negative impacts of wildfire. For example, two narratives that could explain increasing structure loss from wildfires have different implications for identifying and prioritizing mitigation and adaptation strategies. Is increasing area burned—from a range of interacting causes (e.g. climate, fuels, land use)—the main enabler of increasing structure loss, by creating more interactions between wildfire and human communities? Or, are recent trends in structure loss largely driven by increasing development and associated human-related ignitions? Both narratives (and more) are well supported as causes for increasing wildfire disasters in California, for example (17, 22, 24); however, future policy decisions based on trends and causes in one region may not be appropriate in other regions of the West.

Here we characterize the patterns and trends in human and non-human causes of 21st-century wildfires across the West, and focus on structure loss as an acute negative human impact. Our analyses utilize a rich data set of 15,001 wildfires from the 11 Western states spanning 1999–2020 (Fig. 1A), including all fires that resulted in a suppression response, and any associated fire-caused structure loss. *Our data set does not include prescribed fires, unless they escaped prescription.* Annual area burned in our data set is highly correlated with the commonly used Monitoring Trends in Burn Severity data set (*r* = 0.99, *P* < 0.001; *y*-intercept not different from 0, *P* = 0.30; Fig. S1). We adopt the term “fire regime” from the field of ecology, where characteristics of fires are summarized over space and time based on summary statistics from individual fire events, including fire timing, size, and ecological impacts (25, 26). We explicitly quantified biophysical and human components and drivers of fire regimes, including area burned by ignition source, structure density in flammable vegetation, and structure loss as a fire impact. Notably, our work does not integrate other important human drivers or impacts of wildfires, including socioeconomic factors or health impacts from wildfire smoke (e.g. 27, 28).

By integrating structure loss with other metrics reflecting the diversity of wildfires across the West, our work elucidates distinct components of social-ecological fire regimes (29, 30) and how they have changed over the 21st century. Our findings add to a growing body of evidence highlighting the importance of integrating human causes and impacts into our understanding of changing fire activity (e.g. 8, 20–23, 31–33). Critically, we also highlight important variability among Western states, providing necessary context for understanding the causes and impacts of rapidly changing fire activity, and for informing managers, policymakers, and community members seeking to develop regionally appropriate mitigation and adaptation strategies (e.g. 5, 34, 35).

## Results and discussion

### Structure loss from wildfires increased substantially over the past two decades

**Fig. 2.**
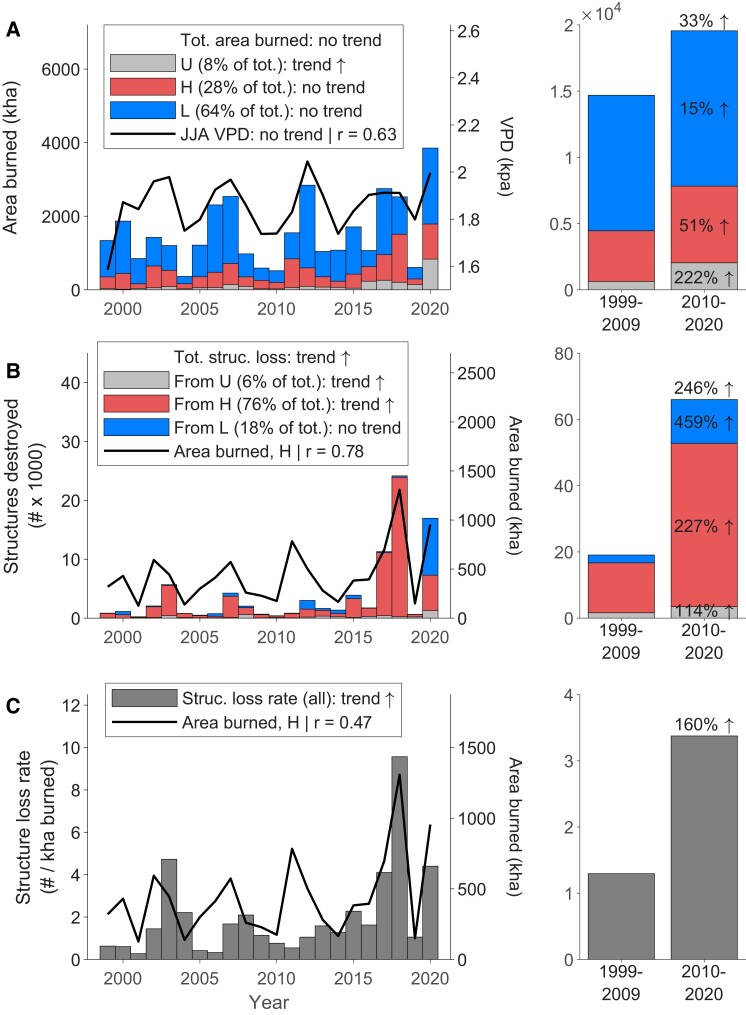
Temporal patterns of area burned and structure loss from wildfires in the western United States. (A) Annual area burned, by ignition source, and annual average June–August vapor pressure deficit (JJA VPD): “H” unplanned human-related ignitions; “L” lightning-caused ignitions; “U” undetermined ignition source. Only annual area burned from undetermined ignition sources exhibited a significant temporal trend over the analysis period. Annual area burned was significantly correlated with JJA VPD. Right panel summarizes statistics over the two halves of the analysis period. (B) Annual structure loss and (C) structure-loss rate (i.e. structures destroyed per 1,000 ha burned) both increased significantly over time, largely from a significant increase in structure loss from unplanned human-related ignitions. Both structure loss and the structure-loss rate were correlated with annual area burned from human-related ignitions. Temporal trends were assessed via the non-parametric Theil-Sen slope test, and correlations were assessed with a Pearson correlation (between log-transformed values); trends and correlations were considered significant at *P* < 0.10 level (see “Materials and methods”).

Annual structure loss from wildfires increased significantly over the analysis period (*n* = 22 years; Theil-Sen slope = 128 structures/year; *P* = 0.06), with over three times more structures destroyed from 2010 to 2020 compared with 1999 to 2009 (+246%, Fig. 2B). Over half (62%) of the total structures destroyed since 1999 occurred during 2017, 2018, and 2020, when annual structure loss was an order of magnitude greater than other years in the record. Trends in structure loss were also evident in the increasing extremes from individual fire events. In the first decade of the 21st century, the 99.9th percentile for wildfire-caused structure loss per fire was 338, whereas over the 2010–2020 period, this increased by an order of magnitude, to >1,500.

The West-wide pattern of increased structure loss was dominated by California, which accounted for 65,551 (77%) of the 85,014 structures destroyed over the 1999–2020 period. Nonetheless, structure loss was higher over the past decade, relative to 1999–2009, in all states except New Mexico (0% change), with changes ranging from +13% (Arizona) to +1796% (Oregon) (Fig. S4). Trends in increasing annual structure loss over the analysis period were significant in California, Oregon, and Washington (*n* = 22; Theil-Sen slope = 62, 2, 5 structures/year; *P* = 0.06, 0.07, 0.04, respectively).

### Increased structure loss not just from increasing area burned

*Wildfire-related structure loss did not increase simply due to increased area burned*. Wildfires have become significantly more destructive over the 21st century, as indicated by the number of structures destroyed per unit area burned (“structure-loss rate”). While area burned showed no significant trend in our 22-year data set (*n* = 22; Theil-Sen slope 25.85 kha/year; *P* = 0.31; Fig. 2A; SI Results), the West-wide structure-loss rate increased significantly over the analysis period (*n* = 22; Theil-Sen slope = 0.087; *P* = 0.03), more than doubling from 1.3 to 3.4 structures destroyed per 1,000 ha burned between 1999–2009 and 2010–2020 (+160%, Fig. 2C). Trends in structure-loss rates were dominated by California, although structure-loss rates were higher over the past decade, relative to 1999–2009, in all states except Arizona (−24%) and New Mexico (−4%), ranging from +7% (Colorado) to +1,063% (Oregon; Figs. 4I and S3). Arizona illustrates the important difference between total structure loss and structure-loss rates: while total structure loss was higher (by 13%) in the recent decade, the structure-loss rate was lower because more area actually burned (Fig. S4).

### Unplanned human-related ignitions result in orders of magnitude higher structure loss

**Fig. 3.**
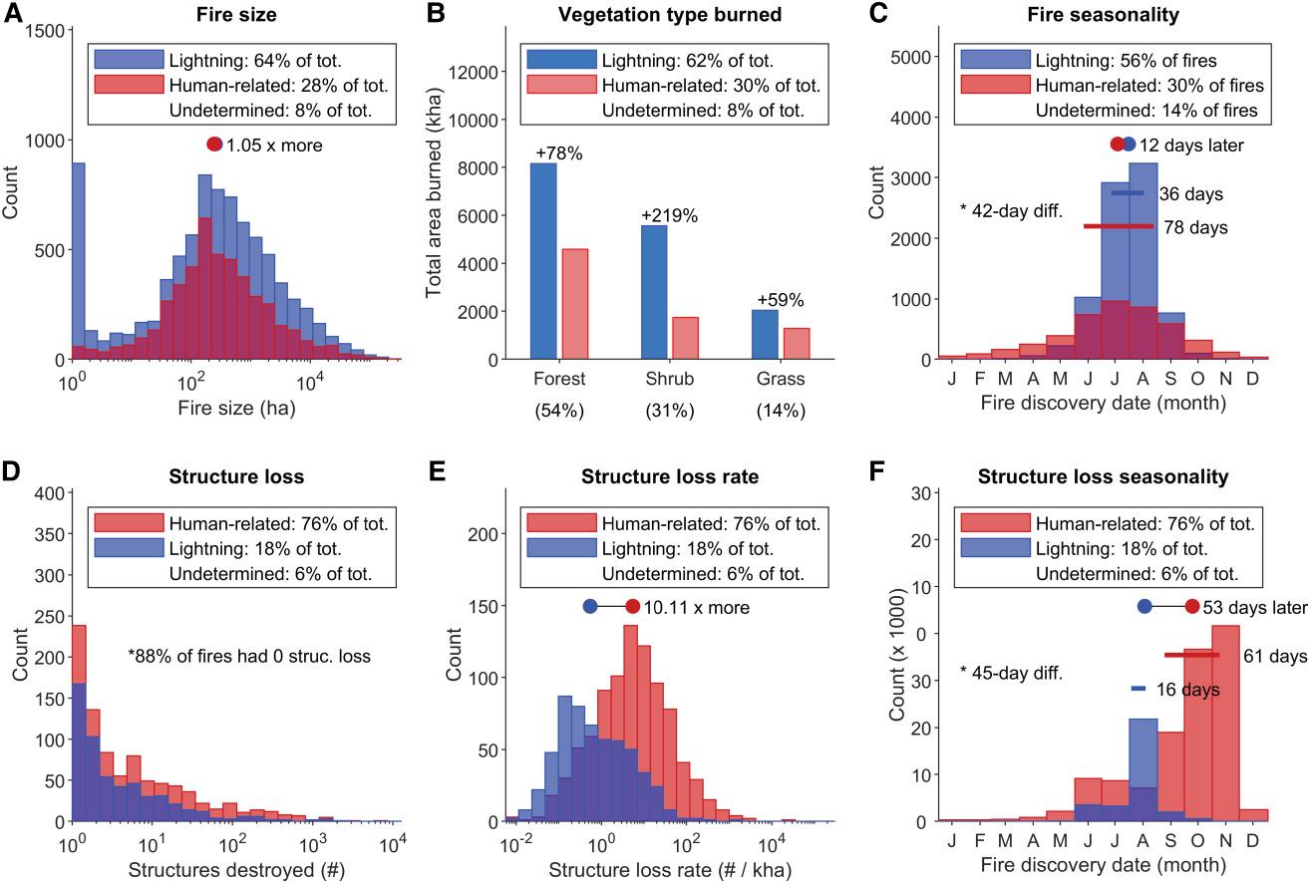
Selected fire-regime attributes by ignition source for the western United States. Fire characteristics (A–C) and fire impacts (D–F) differ significantly between fires from human-related and lightning ignitions, as summarized from 15,001 fire events between 1999 and 2020. Legends include the percent of total area burned (A, B) and total number of fires (C) accounted for by fires from each ignition source. Fire seasonality (C) and structure-loss seasonality (F) include bars spanning the season length, with * indicating difference in season length. Legends across the bottom row (D–E) include percentages for the total number of structures destroyed from fires originating from each ignition source, and total number of fires (F). For all panels except (B), “count” refers to the number of fires; median values are displayed as dots (excluding structure loss [D]; medians = 0) and are significantly different, based on a Wilcoxon rank-sum test (0.000 < *P* < 0.005). “Structure-loss rate” (E) is defined as the number of structures destroyed per 1,000 ha burned in a single fire event. Note: analysis for vegetation type burned is from a subset of fires (*n* = 6321, accounting for 75% of the total area burned in the larger data set); thus, percentages in legend differ from (A) (see “Materials and methods”).

A striking pattern in 21st-century statistics is the critical difference between wildfires started from human-related compared with lightning ignitions. The median structure-loss rate in wildfires from human-related ignitions was 10 times higher than from lighting-ignited wildfires (Fig. 3E; Wilcoxon rank-sum *z*-statistic = −16.82; *P* < 0.001). Likewise, extremes in total structure loss (defined by the 99th percentile) were an order of magnitude higher for wildfires from human-related ignitions compared with lightning ignitions (Fig. 1B). As a consequence, 76% of structures destroyed across the West were attributable to wildfires from human-related ignitions (Fig. 3D). The outsized impacts of human-related ignitions on structure loss reflect in part their non-random spatial locations: human-ignited wildfires tend to occur close to structures (20, 21) and in areas with higher grass vs. tree cover (Fig.3B; 31).

### Consequences of human-related ignitions interact with high fuel aridity

Wildfires from human-related ignitions had outsized impacts on structure loss when they happened in the late summer and fall, when fuel aridity is high and lightning ignitions are rare. For example, while the West-wide fire season for human-related ignitions (defined by the interquartile range of fire starts; 22) was 42 days longer than for lightning-ignited fires and peaked in late July (Fig. 3C), the peak in structure loss from human-related wildfires occurred in late October (Fig. 3F). This pattern reflects, in part, the most destructive wildfires from recent years (2017, 2018, 2020; Fig. 2B). Despite the strong influence of California, this asynchrony was seen across eight of the 11 Western states (Fig. S5). In the Southwest (e.g. New Mexico, Arizona), the peak in structure loss from human-related ignitions occurred in the middle to late spring, when low fuel moisture can precede the start of the lightning-caused fire season (Fig. S5); such conditions apply to New Mexico's 2022 Calf Canyon Fire, which ignited in May from continued smoldering combustion after a January pile burn (36).

Higher structure loss from human-related ignitions outside of the lightning-caused fire season, especially in late summer and fall, results specifically from the combination of seasonally high fuel aridity and high-wind events (21, 37, 38). This combination leads to more extreme fire behavior—a mechanism in part supported by more extreme fire weather and fire behavior in human-ignited wildfires in California (31). This pattern also highlights how human-related ignitions can interact with increased fuel aridity and longer periods of high fire danger annually, due to anthropogenic climate change and the varying sensitivity of vegetation to resulting increases in aridity (14–17, 37, 39, 40). Human-related ignitions will have increasingly destructive consequences as climate change causes higher fuel aridity, for more days each year.

### Increasing structure loss largely explained by human factors

**Fig. 4.**
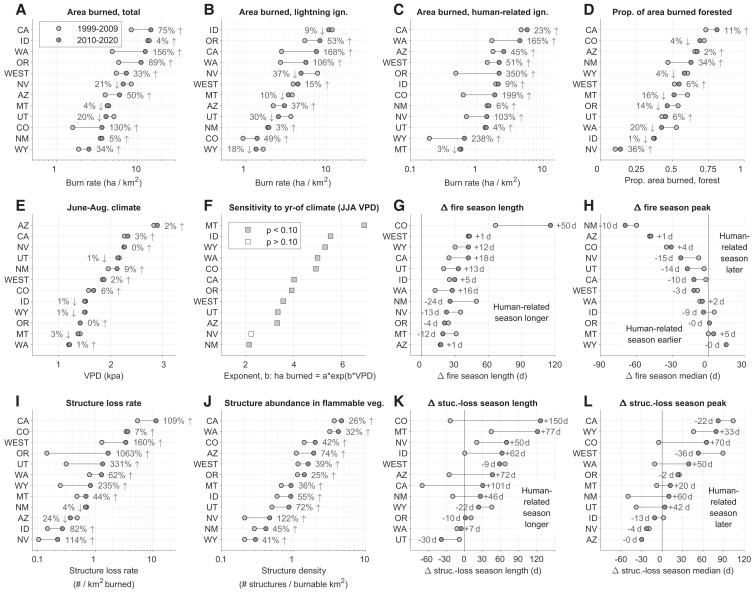
Rank and change in fire-regime attributes for the western United States. Area burned by ignition source (A–C), and proportion of total area burned in forest vegetation (D). Climate (E), summarized by mean June–August VPD (VPD). Fire-climate relationships (F), summarized by the *b* parameter for regression models predicting annual area burned as a function of June–August vapor pressure deficit (*n* = 22, over 1999–2020). Difference in fire-season length (based on fire start dates; G) and the peak in fire ignitions (i.e. median start date; H) between human-related and lightning ignitions. Structure loss (I), structure abundance (J), and differences in the timing (K) and peak (L) of fire-related structure loss (based on fire start dates). The ordering of states differs among panels, based on the rank for each specific metric.

Total West-wide area burned from human-related ignitions was 51% higher from 2010 to 2020 compared with 1999 to 2009. Although not detected as a statistically significant annual trend (Fig. 2A; SI Results), the West-wide pattern of higher area burned from human-related ignitions was exhibited across all states except Montana (−3%), ranging from +4% (Utah) to +350% (Oregon; Figs. 4C and S6). More area burning from human-related ignitions occurred in conjunction with a 39% higher density of structures in flammable vegetation across the West, a pattern exhibited in all Western states, ranging from +25% (OR) to +122% (UT) (4J).

**Fig. 5.**
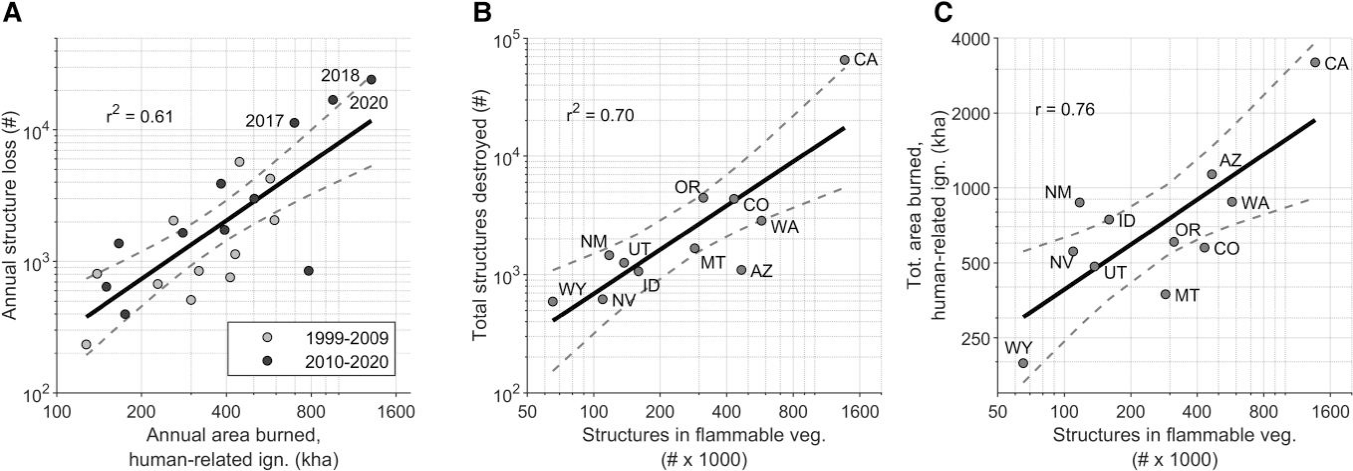
Predictors and correlates of annual and total structure loss from wildfires in the western United States. (A) Annual area burned from human-related ignitions explains 61% of the variability in annual structure loss. (B) Total structure loss from wildfires at the state level (1999–2020) is well explained by structure abundance in flammable vegetation (from 2010). (C) Total area burned from human-related ignitions at the state level is well correlated with structure abundance in flammable vegetation (from 2010). See Fig. S3 for the same relationships in (A) and (C), using area burned from human-related and undetermined ignitions (*r*^2^ = 0.68 for A, and *r* = 0.79 for C).

Strikingly, these two elements—area burned from human-related ignitions and structure density in flammable vegetation—helped explain the majority of variability in structure loss across the West, in time and space. For example, 61% of the variability in annual structure loss from 1999 to 2020 was explained by total annual area burned from human-related ignitions (log–log relationships, *n* = 22, *r*^2^ = 0.61, *F* = 31.4, *P* < 0.001; Figs. 5A and S3). In contrast, annual area burned from lightning-caused wildfires explained only 28% of the variability in structure loss over this period (log–log relationship, *n* = 22, *r*^2^ = 0.28, *F* = 7.82, *P* = 0.011). Across Western states, 70% of the variability in total structure loss from 1999 to 2020 was explained by regional variability in the abundance of structures in flammable vegetation (log–log relationship, *n* = 11, *r*^2^ = 0.70, *F* = 20.6, *P* = 0.001; Fig. 5B).

Structure abundance in flammable vegetation not only exposes structures to wildfires, but it also reflects human-related ignition pressure. Structure abundance (from 2010) was well correlated with total area burned by human-caused ignitions over the entire 1999–2020 record (log–log relationship, *r* = 0.76, *P* = 0.006; Fig. 5C). In contrast, structure abundance was uncorrelated with total area burned by lightning-caused ignitions (log–log relationship, *r* = 0.16, *P* = 0.64). This pattern is consistent with previous research documenting the coincidence of human ignitions near homes (21), highlighting that with human development comes accidental human ignitions. *Thus, an expected outcome of the increased structure abundance across the West in recent decades (*Fig. 4J*) is an increase in both area burned from human-related ignitions and wildfire-caused structure loss, predominantly from those very ignitions*.

### Social-ecological fire regimes shifting to more human-caused burning and structure loss

**Fig. 6.**
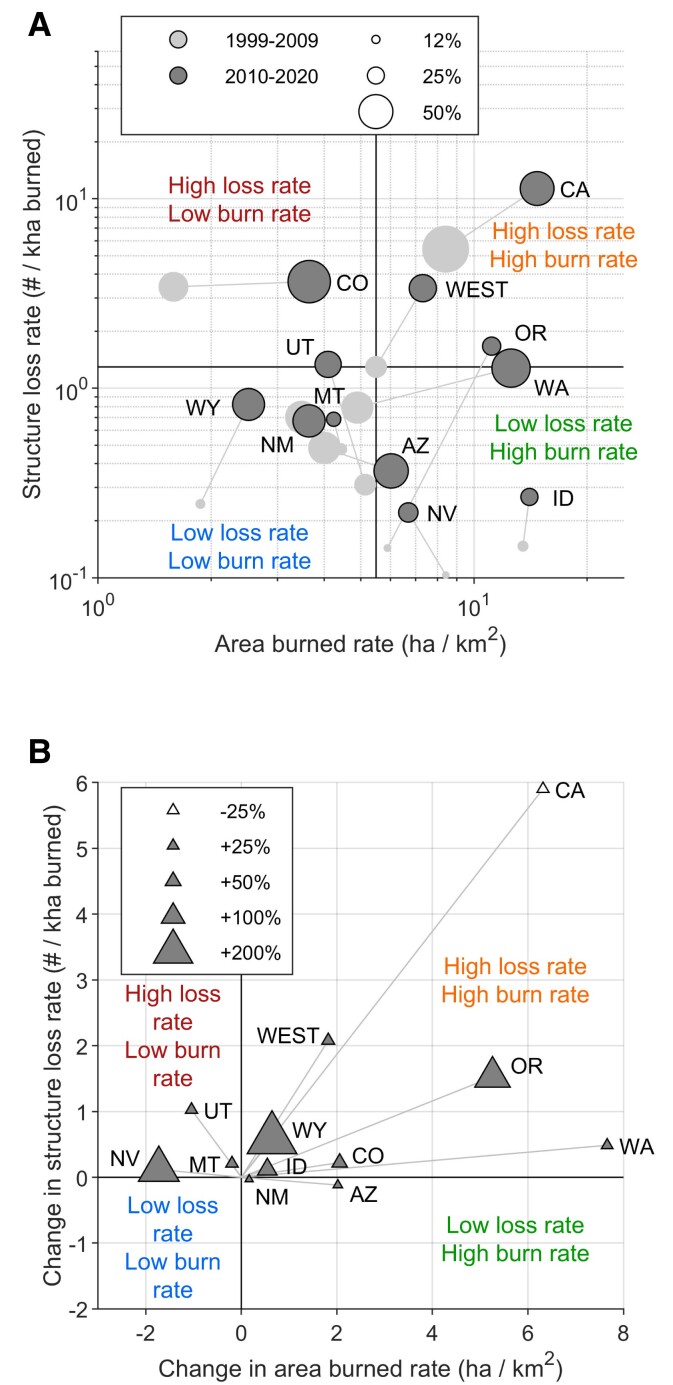
Changes in core components of social-ecological fire regimes in the western United States over the first two decades of the 21st century. (A) Western states plotted based on the rates of area burned (*x*) and structure loss (*y*), with circle size scaled to the percent of total area burned from human-related and undetermined ignitions. Quadrants are labeled relative to the West-wide rates from 1999 to 2009. Note: axes are log scales. (B) Changes in rates of area burned (*x*) and structure loss (*y*) between the two analysis periods (i.e. radial vector analysis of A), with triangle size scaled to change in percent of total area burned from human-related and undetermined ignitions (+/gray indicates more area burned from human and undetermined sources). Note: axes are on linear scales, to illustrate absolute change and accommodate negative changes.

We classified Western states into four fire regimes based on standardized rates of total area burned and structure loss, and the percent of area burned from human-related ignitions. The state-level classification is critical, in part because decision-making and resilience planning is happening at these scales (e.g. 35). Our classification reveals notable variability among states based on fundamental human and environmental factors (Fig. 6A). Below, we refer to the standardized rate of burning (ha burned/km^2^ of flammable vegetation) and structure loss (#/kha burned) as “high” or “low,” relative to the West-wide rates from 1999 to 2009.

“***Low burn–High loss***” fire regimes are epitomized by Colorado, which has a high structure-loss rate, despite a low area-burned rate. This combination is indicative of high area burned from human-related ignitions, late in the fire season and near structures around flammable vegetation. Of all Western states, Colorado had the highest proportion of area burned from human-related ignitions—51% over the 2010–2020 period and 47% over the entire 1999–2020 period (Fig. S6)—consistent with fire starts close to structures (21). Further, around 38% of structure loss in Colorado occurred in late summer and early fall, when fuel aridity is typically highest and when high-wind events create extreme fire weather. The 30 December 2021, Marshall Fire in Colorado exemplified this pattern (but is not included in this data set); at the time of publication this was the state's record-setting wildfire for structure loss, despite its size (Fig. 1B), and had a yet-undetermined ignition source (although it occurred without regional lightning activity).

“***High burn–High loss***” fire regimes are epitomized by California, which alone accounted for 76% of all structures destroyed by wildfire, and 20% of the total area burned over 1999–2020. While high structure loss is consistent with the high rate of burning, California stands out from other states like Idaho, Washington, and Oregon (in the 2010–2020 period), which experienced similar rates of burning with lower structure-loss rates. Notably, California had the second highest proportion of total area burned from human-related ignitions over the 1999–2020 period (45%, second to Colorado), which actually decreased from 55 to 39% between 1999–2009 and 2010–2020 (Figs. 6B and S6). California also had the highest structure density in flammable vegetation (Fig. 4J).

Together, regions with “…High loss” fire regimes would benefit most from intensive efforts to reduce human-related ignitions, particularly in the critical times of the fire season when fuel aridity is highest, and from efforts focused on utilizing fire-resistant building materials and careful consideration of nearby vegetation (32). In these regions, it is when and where area burns—not total area burned itself—that is most threatening to structures.

***Low burn–Low loss*** or ***High burn–Low loss*** fire regimes characterize much of the West (see also SI Results). Although we focus on structure loss as an acute negative human impact of wildfires, most Western states experience low loss rates (Fig. 6A). In fact, most wildfires in the West do not destroy structures: 88% of the 15,001 fires analyzed, accounting for 45% of the total area burned, had no associated structure loss (Fig. 1A). Considering lightning-ignited fires only, 94% (accounting for 53% of the total lightning-ignited area burned) had no associated structure loss. Thousands of hectares burn annually in the West, the majority from lightning-ignited fires, with no structure loss. A minority of events result in structure loss that most demand and capture our attention.

Together, regions with “…Low loss” fire regimes accommodate landscape burning from wildfires, including the many ecological benefits (41, 42), without incurring high structure loss. This is true across states with varying levels of structure density in flammable vegetation and human-related ignitions. Careful planning in these regions could help avoid the pitfalls experienced among “…High loss” regions, including crossing any thresholds in human-related ignitions, and maintaining safe landscape burning to support continued below-average structure-loss rates.

Our characterization of social-ecological fire regimes is necessarily coarse in space and time. We present each state as an example of variability in key characteristics, useful for comparison, but not implying that states have been or will remain unchanged. Changes over the past decade exemplify how state-level and West-wide patterns are strongly determined by extreme events and extreme years. In Oregon, for example, record-setting structure-loss rates during the 2020 fire season moved the state from the “Low loss” into the “High loss” category (Fig. 6A).

The diversity in social-ecological fire regimes across Western states contrasts with the similarity in trends experienced over the past two decades (Fig. 6B). Six of the 11 Western states moved toward “High burn–High loss” regimes, with California, Oregon, and Washington exhibiting the largest changes. Most states also experienced an increased proportion of area burned from human-related ignitions, particularly lower population states like Wyoming (+16%), Nevada (+13%), and Oregon (+11%). Only California (−16%) and Arizona (−2%) experienced decreases in proportion of area burned from human-related ignitions. Even with the decrease, 39% of area burned in California between 2010 and 2020 originated from human-related ignitions, suggesting this state likely surpassed any “threshold of concern” (29) with respect to human-related ignitions by the early 21st century (Fig. S6). Three of the remaining states—Utah, Nevada, and Montana—moved toward “*Low burn–High-loss*” regimes, experiencing increased structure-loss rates, despite small or moderate decreases in area burned. Only Arizona and New Mexico experienced decreased structure-loss rates over the two decades.

## Conclusions

Our study reveals key drivers of the wildfire crisis in the West, and therefore when, where, and which “levers” can be pulled to reduce the chances of future fire disasters. We focused on structure loss as an acute negative impact of wildfires, highlighting a significant increase in structures destroyed by wildfires, by over 3× between 1999–2009 and 2010–2020 (Fig. 2B). Critically, we also show that wildfires have become more destructive, with the number of structures destroyed per 1000 ha burned increasing, by 160% between the last two decades (Fig. 2C). This West-wide pattern was dominated by California and from events in just three recent years (2017, 2018, 2020), but nearly every Western state exhibited higher structure-loss rates over the second decade of the 21st century (Figs. 4I and S4).

Increased loss rates indicate that *wildfire-related structure loss did not increase simply due to more area burning.* Likewise, although our analysis did not resolve structure exposure to individual wildfires, we estimate that the proportion of structures destroyed by wildfire outpaced higher structure abundance in flammable vegetation alone (Table S1). The causes of increased structure loss thus reflect complex human–environment interactions, not “either” more area burning “or” increased development, but both, and more. When and where human-related ignitions occur, how many structures are built among flammable vegetation, and how climate and land use affect fuel abundance and fuel aridity over timescales of days to decades are all interacting to drive trends in structure loss.

Recognizing the complex drivers of fire disasters has important implications for mitigation and adaptation. For example, efforts to reduce structure loss must look beyond simply limiting area burned generically. In fact, across the West 88% of wildfires, accounting for 45% of the total area burned, had no associated structure loss (Fig. 1A). While not explicitly assessed here, much of this burning is not a crisis *per se* and can provide resource benefits (41–44). The wildfire crisis generally and structure loss specifically are largely driven by extreme events, highlighted clearly in recent years (Fig. 1B). The overwhelming majority of wildfires that result in structure loss are started by human-related ignitions, and they are occurring in regions with increasingly high structure density within flammable vegetation (21,; Fig.4J). Consequently, the number of structures destroyed by wildfire in the West in any single year was well explained by the total area burned from human-related ignitions; and, total structure loss in a state over the past two decades was well explained by the number of structures in flammable vegetation (Fig. 5). Both elements have increased between the past two decades—area burned from human-related ignitions by 51%, and structure density by 39% (Fig. 4)—and are leading contributors to increased structure-loss rates.

While numerous studies highlight increased fuel aridity from anthropogenic climate change as a key enabler of rising wildfire activity in the West (6, 14–17), in parallel to these changes we have shown that human factors operating over shorter time scales are increasingly contributing to wildfire disasters. The consequences of human-related ignitions, specifically *when and where fuel aridity is high and lightning ignitions are rare*, are becoming magnified in the context of climate change. Therefore, efforts to reduce human-related ignitions will be increasingly important beyond historical lightning-caused fire seasons, for example in spring, fall, and even winter months (Figs. 3F and S5).

The patterns highlighted here varied widely across the West, emphasizing the importance of understanding spatial and temporal characteristics of wildfires—fire regimes—as integrated social-ecological phenomena (29, 30, 45). Mitigating anthropogenic climate change, given its impacts on fuel aridity (6, 14, 19), is a clear overarching necessity for addressing the wildfire crisis. At smaller scales of individual states, policymakers and managers may benefit from emphasizing other aspects driving increased structure loss, including structure expansion into flammable vegetation, increased ignition from human-related sources, or in states with below-average structure loss, reducing the chances of lighting-ignited fires spreading into developed areas (32, 43). Mitigation and adaptation approaches in predominantly rural states, like Wyoming, Montana, and Idaho, may look different than in more densely populated states, like California, Colorado, and Washington. Additionally, states with low structure-loss rates may look to those with high structures loss as harbingers of future change, and ideally glean ways to avoid similar outcomes as structure expansion and densification trends continue. Two clear implications emerge from this and other recent work (20, 21, 32, 33) to help prevent wildfires from becoming disasters: reduce unintentional human-related ignitions, *particularly near homes and during periods of extreme fire danger*; and carefully consider if and how structures are built, including building with fire-resistant materials, minimizing flammable vegetation near structures, and providing mechanisms to do so equitably across socioeconomic conditions.

## Materials and methods

We utilized a unique data set that captures multiple aspects of the causes and impacts of wildfires from 1999 to 2020; the ICS-209-PLUS data set (describe below) forms the basis of our analyses of fire sizes, area burned, ignitions source, structure loss, and estimated incident-command costs. To complement these analyses, we draw on additional data sets to summarize the number of structures in flammable vegetation, the dominant vegetation types burned, and fire-conducive annual climate conditions. We focus on West-wide and state-level patterns to help inform local, regional, and state-level efforts addressing the wildfire crisis (e.g. 35). For other audiences, we provide a subset of summaries at management and ecologically relevant scales in SI Results (i.e. geographic area coordination centers, level II ecoregions; Figs. S7–S12).

### Fire characteristics and impacts: ICS-209-PLUS data set

We obtained fire characteristics and impacts from point-specific wildfire incident-command reports from the ICS-209-PLUS data set, published by St. Denis et al (46) and updated here from 2018 to 2020. This data set includes wildfires that resulted in an emergency response; it does not include prescribed fires or agricultural burning—planned human-set fires—unless the fire escaped prescription and transitioned into a wildfire. The distilled and cleaned database is mined from the public archive of the United States National Incident Management System/Incident Command System (NIMS/ICS) Incident Status Summary Form or ICS-209 report. An ICS-209 report is completed for any significant wildfire that is under full suppression management strategy that exceeds 40.5 ha (100 acres) in fuel classified as timber, 121 ha (300 acres) in fuel classified as grass and brush, or has a type 1 or 2 incident management team assigned (www.nifc.gov). The ICS-209-PLUS database compiles the ICS-209 reports, capturing critical details of significant wildfire incidents (46). We used the following fields in the current work: incident name, year, discover day of year, total area burned, ignition source (discussed further below), total estimated incident management costs, and total structures destroyed. The “total structures destroyed” field accounts for confirmed structures destroyed in a fire event, including residences, commercial property, and other minor structures affixed to a permanent site (e.g. barns, sheds) (47).

The West-wide, 1999–2020 data set used here includes 15,001 individual incident summary reports representing 34 million ha burned, $29.7 billion estimated suppression costs, and 85,014 structures destroyed by wildfire. Notably, the West accounts for 79% of burned area, 96% of estimated suppression costs, and 81% of wildfire-related structure loss across the conterminous United States (1999–2020). Due to the complex nature of ICS-209 reporting and potential for reporting uncertainty, some manual quality control was performed on the data set to capture and address duplicated records and missing information on the ignition cause. For all fields except ignition source (SI Methods), quality control and assurance is described by St. Denis et al. (46).

We categorized ignitions into four categories: human-related, lightning, undetermined, and other. For “human-related” we included any ignition not attributed to lightning (“natural”) or undetermined sources; examples of human-related ignition sources include: “Recreation and ceremony,” “Arson/incendiarism,” “Power generation/transmission/distribution,” “Debris and open burning,” “Equipment and vehicle use,” and “Misuse of fire by a minor.” Given our focus on human-related ignition sources, we implemented additional steps to update fires with an undetermined cause (SI Methods). After updating cases, 1947 fires (13% of total) had an undetermined cause. The majority of these fires occurred within the last several years, both because of the active fire years of 2017, 2018, and 2020, and because in many cases investigations for these fires are ongoing. Finally, there were 165 fires with an ignition source of “other” in the final data set, accounting for 1.1% of all fires by number, 0.4% of all area burned, and 0.2% of all structures destroyed. Given the small fraction of fires with this cause, we combined events from the “other” category with events with “undetermined” ignition sources.

### Structures in flammable vegetation

We characterized the density of structures in flammable vegetation using two data sets. Our metric of structure density is purposefully coarse and does not reflect the precise number of structures exposed to wildfires. In addition, it does not delineate among urban areas, wildland areas, or their interface. Nonetheless, the patterns highlighted here for structure density in flammable vegetation—in space and time—are broadly consistent with other work characterizing the growth of the wildland-urban interface (e.g. 8).

To identify potentially flammable landscapes, we leveraged the United States Forest Service (USFS) spatial data set of probabilistic wildfire risk components (48) to identify all potentially flammable land cover across the West. Specifically, we used the burn probability (BP) surface which was generated for the conterminous United States using the geospatial Fire Simulation (FSim) system developed by the USFS Missoula Fire Sciences Laboratory (49). The BP-gridded product represents the probability of a pixel burning under current conditions (baseline 2014). We created a binary grid depicting flammable (“1”) and non-flammable (“0”) landscapes by labeling all pixels with a BP greater than zero as flammable. This classification is purposefully broad to capture a majority of wildland fire potential across the West in various vegetation types.

To quantify structure abundance, we used the Historical Settlement Data Compilation for the United States (HISDAC-US) (50). The HISDAC-US database offers historical gridded settlement layers derived from property records compiled in the Zillow Transaction and Assessment Dataset (ZTRAX) and describes the built environment of most of the conterminous United States back to the year 1810 at fine temporal (5 years) and spatial (250 meters) granularity using different settlement measures. Specifically, we leveraged the Built-up Property Records (BUPR) gridded layer (51) to quantify the number of properties with structures within flammable landscapes. We used the 2010 data when comparing fire and structure-loss statistics spanning the entire 1999–2020 period, and we used the 2005 and 2015 intervals when assessing the difference between the 1999–2009 and 2010–2020 periods. In all cases, the structure abundance data come from approximately the middle of the analysis time period.

Finally, we calculated structure density in flammable landscapes by combining these two metrics. We aligned the projection and spatial resolution of the flammability (30 m) and structure abundance metrics (250 m) using a nearest neighbor resampling technique. Then, we multiplied the two metrics on a per-pixel basis. Pixel values labeled as flammable (“1”) in the resampled BP raster were thus assigned the value of the matching BUPR pixel (# of structures). We then summarized this product within each state, calculating the sum of structures within flammable vegetation.

### Vegetation burned

To quantify the general vegetation types burned by wildfires in our analyses, we used fire events from the ICS-209-PLUS data set that co-occurred with fires perimeters delineated by the Fire Events Delineation (FIRED) database (52). This was necessary because the ICS-209-PLUS data are point specific. Specifically, the fire perimeters of a subset of 6,367 incidents, accounting for 76% of the total West-wide area burned and 95% of structure loss, were overlaid with the USFS Landscape Change Monitoring System (LCMS) data set (53, 54) to obtain the percent area of grassland, shrubland, and forest cover types within each fire perimeter. The LCMS database provides standardized annual maps of land cover and landscape change from 1984 to present, based on *in situ* field data and optical satellite imagery. The granularity of land cover classes combined with annual temporal resolution of the LCMS allow for this calculation to be performed for the year prior to the fire. Analysis was performed in the Google Earth Engine platform. The similarity in key summary statistics (i.e. % area burned from lightning vs. human-related ignitions) from the full ICS-209-PLUS (Fig. 3A) and the subset of fires from the FIRED database (Fig. 3B) indicate that the FIRED subsample was representative of the larger data set.

### Fire-conducive climate

We used vapor pressure deficit (VPD) as a broad proxy for fuel moisture and the flammability of vegetation. VPD is a widely used metric to represent climate suitability for burning, and it is well correlated with area burned at multiple times scales, across the West and globally (e.g. 1, 17, 39). Specifically, we used the annual mean June–August VPD as the single predictor variable in regression models predicting the log of annual area burned, at the West-wide scale and for each region in regional analyses (e.g. states). VPD estimates were obtained from GRIDMET, a daily high-resolution (∼4 km) surface meteorological data set available from 1979 to present (55). These data and summaries were accessed and generated within the Google Earth Engine platform (56).

### Statistical analyses and decadal summaries

We assessed trends in annual area burned, structure loss, structure-loss rates, and VPD using the non-parametric Theil-Sen slope estimator. To compare fire-regime attributes of fire size and structure-loss rate between ignition sources, we compared median values from among all fires from human-related and lighting ignitions using the non-parametric Wilcoxon rank-sum test. Finally, to assess relationships between metrics, we used regression analyzes when there was a reasonable *a priori* direct mechanistic link between the predictor and response variables (e.g. area burned and structure loss); and we used correlation analysis when there was an indirect link between two variables (e.g. structure density in flammable vegetation and total area burned from human-related ignitions). For regression and correlation analysis the following variables were log-transformed because they were log-normally distributed (e.g. Figs. 2 and 5): annual area burned, structure loss, structure-loss rate, and structures in flammable vegetation.

Statistical tests were performed in MATLAB software (MathWorks), and we consider trends, correlations, or explained variance significant at the alpha = 0.10 level. For trends over time in particular, this high alpha level accounts for the lower statistical power from 22 years of data, and the non-parametric analyses.

Finally, to elucidate variability in fire activity and impacts, we calculated summary statistics from 1999–2009 to 2010–2020. We did not statistically compare these periods (approximately decades), given no *a priori* expectation of a shift around 2009–2010. Decadal summaries highlight what humans are experiencing, reacting to, and questioning, regardless of statistical significance. For example, significant federal funding is being directed at the wildfire crisis in the West through the 2022 Infrastructure Investment and Jobs Act (57). Such actions are largely in response to recent fire disasters in 2017, 2018, and 2020, which in turn are within expected trends predicted under anthropogenic climate change (13, 58).

### Social-ecological fire regimes

We classified Western states into one of four social-ecological fire regimes based on three components of fire activity and human impacts: total area burned, standardized to burnable area within each region (kha/km^2^ burnable vegetation); total structure loss, standardized to total area burned (#/kha burned); and the percent of total area burned from human-related ignitions (SI Methods). While we focus on total structure loss, this metric was well correlated with estimated costs of wildfire management during the incident-command phase, another acute negative human impact (*n* = 22; *r*^2^ = 0.71; *P* < 0.05; Fig. S2). Within the two-dimensional space defined by standardized area burned (“Area burned rate”) and standardized structure loss (“Structure loss rate”), a region falls into one of four categories, described relative to the West-wide values over the 1999–2009 period: (1) Low burn–High loss; (2) High burn–High loss; (3) Low burn–Low loss; and (4) High burn–Low loss. These groupings integrate foundational biophysical drivers of fire that have operated for millennia (e.g. variability in climate and vegetation), with human-related factors influencing the causes and consequences of wildfires (e.g. structure density in flammable vegetation).

## Supplementary Material

pgad005_Supplementary_Data

## Data Availability

Data, code, and figures associated with this work are publicly available via the Dryad data repository (https://doi.org/10.5061/dryad.5hqbzkh9m) and GitHub (https://github.com/HigueraLab).
