# Supplementary material for: Shifting social-ecological fire regimes explain increasing structure loss from Western wildfires
Source: PNAS Nexus. 2023 Feb 1;2(3):pgad005. doi: 10.1093/pnasnexus/pgad005 (PMC10019760; doi:10.1093/pnasnexus/pgad005)
Supplement: pgad005_Supplementary_Data [file pgad005_supplementary_data.pdf]

PNAS nexus

Supporting Information for

Shifting social-ecological fire regimes explain increasing structure loss from Western wildfires

Philip E. Higuera[1*], Maxwell C. Cook[2,3], Jennifer K. Balch[2,3], E. Natasha Stavros[2], Adam L. Mahood[2,4], Lise A. St. Denis[2]

[1]Department of Ecosystem and Conservation Sciences, University of Montana, Missoula, MT, 59812, USA
[2]Earth Lab, CIRES, University of Colorado, Boulder, CO, 80309, USA
[3]Department of Geography, University of Colorado, Boulder, CO, 80309, USA
[4]Water Resources, Agricultural Research Service, United States Department of Agriculture, Fort Collins, CO, USA

***Corresponding author:** Philip Higuera, philip.higuera@umontana.edu, 32 Campus Dr., Missoula, MT 59812.

This PDF file includes:

Supporting methods
Supporting results
Table S1
Figures S1 to S12

Supporting methods

Updated ignition causes

Given our focus on human-related ignition sources, we implemented additional steps to update fires with an undetermined cause. First, we updated the cause identified in the original ICS-209 report with any additional information provided by the final reporting for the fire in the associated Fire Protection Analysis Fire-occurrence Database (FPA/FOD) report (1); because the latter is completed after the ICS-209 report, it often has a cause established that is recorded as "unknown" or "undetermined" in the original ICS-209 report. Through this process the cause of 1730 (11.5%) of the 15,001 fire events was updated; of the wildfires that were originally classified as "undetermined" or "other" and later have a cause attributed, the majority ended up classified as human-related ignitions (c. 70% by number, and c. 60% by total area burned). Second, for the remaining fires with an undetermined cause, we systematically searched online fire databases and media to learn if a cause had been attributed to a fire event since its inclusion in the ICS-209-PLUS database. Online fire databases included InciWeb, CalFire, regional Coordination Center annual reports, and United States Forest Service reports. Examples (all accessed in March 2022) include:

- 2020 Inchelium Complex Fire in Washington: https://inciweb.nwcg.gov/incident/7177/
- 2018 Thomas Fire in California: https://www.fire.ca.gov/incidents/2017/12/4/thomas-fire/
- Numerous fires from 2017 and 2018 in the Pacific Northwest: https://gacc.nifc.gov/nwcc/content/pdfs/archives/2018_NWCC_Annual_Fire_Report_FINAL.pdf, https://www.fs.usda.gov/Internet/FSE_DOCUMENTS/fseprd572804.pdf

Media sources included regional and national news media, and wildfire-related professional sources. Example includes (all accessed in March 2022):

- 2020 Dolan Fire in California, "The Californian": https://www.thecalifornian.com/story/news/2020/08/19/california-deputies-arrest-arson-suspect-dolan-fire-big-sur/5609484002/
- 2020 Zogg Fire, California, "Wildfire Today": https://wildfiretoday.com/2021/03/23/investigators-determine-tree-contacting-pge-power-line-started-zogg-fire/?hilite=zogg+fire
- 2020 Silverado Fire, California, "NBC News": https://www.nbcnews.com/news/us-news/cause-southern-california-fire-forced-thousand-evacuate-may-be-lashing-n1244973

We used contextual information – e.g., time of year, lack of known lightning events during the time window of ignition, and coincidence with high winds and/or other fires started by human-related sources as bolstering evidence when a media source speculated that a fire was human-caused. In total, the cause of 141 fires was updated from "undetermined" or "other" to either "human-related" or "lightning," with the full list of fires and sources used to update the cause provided in the computer code associated with this paper.

Supporting results

Trends in area burned

West-wide annual area burned showed no significant trends over the 22-yr analysis period ($n = 22$; Theil-Sen slope = 25.85 kha/yr 0, $p = 0.31$), although total area burned was 33% higher over 2010-2020 compared to 1999-2009 (Fig. 2A, main text). This lack of trend differs from numerous studies that highlight significant increasing trends in area burned across the West, which typically include a longer time period, extending back to the mid 1980's (e.g., 2). Separated by ignition source, neither area burned from lightning-caused nor unplanned human-related ignitions increased over the analysis period ($n = 22$; Theil-Sen slope = 5.44, 12.47 kha/yr, $p = 0.78$, 0.34, respectively). Area burned from fires with an undetermined ignition source did increase over the analysis period ($n = 22$; Theil-Sen slope = 7.58 kha/yr; $p < 0.001$), largely because of the large number of fires in this category in the last five years of the dataset.

Fire-climate relationships and trends in vapor pressure deficit

Consistent with a large body of prior work, we found strong relationships between annual area burned and mean annual June-August vapor pressure deficit (VPD), a proxy for fuel aridity and thus flammability (3–5). West-wide, variability in total annual area burned was significantly correlated with average June-August VPD (Fig. 2A, main text; $n = 22$; Pearson correlation with log area burned = 0.63; $p = 0.002$). This was also exhibited for all states except Nevada ($n = 22$; Pearson correlation with log area burned 0.39-0.92; $p < 0.10$; Fig. 4F, main text). Nevada, where wildfires burn primarily in shrub-dominated vegetation (Fig. S5), exhibited significant correlation between area burned and prior-year VPD, a pattern well described previously, linked to the requirement of prior-year moisture to support fuel growth (6).

The strength of correlation between area burned and climate varied across states and by ignition type (Fig. S4). For example, in California, VPD was most strongly correlated with area burned from human-related ignitions ($r = 0.60$; $p = 0.003$) and was not significantly correlated with lightning-ignited area burned ($r = 0.29$; $p = 0.19$). In contrast, in Montana, the correlation between VPD and lightning-ignited area burned was stronger ($r = 0.92$, $p < 0.001$) compared to human-related area burned ($r = 0.72$, $p < 0.001$).

At the West-wide scale VPD did not exhibit a significant trend over the analysis period. Previous work has demonstrated clear trends in VPD over longer time periods (5, 7), so we take this lack of trends as reflecting the shorter time period and averaging over the entire West. At the state level, VPD increased significantly in California (Fig. S3), which accounted for ca. 20% of the total area burned across the West over the entire record.

Social-ecological fire regimes of the West: states with low structure loss rates

The main text includes full descriptions of "...High loss" fire regimes and a summary of "...Low loss" regimes. Here we more fully describe "...Low loss" fire regimes, as illustrated in Figure 6 in the main text.

"Low burn–Low loss" regimes are epitomized by Wyoming, New Mexico, and Montana. While each state has still experienced fire events with devastating human impacts (e.g. 8), in the context of the West, rates of burning and structure loss were lower than average. These states also have

relatively low structure abundance in flammable vegetation (Fig. 4J, main text). Importantly, the proportion of area burned from human-related ignitions varied widely among these four states, from a 1999-2020 low of 13% in Montana to a high of 41% in New Mexico (Fig. 6, main text; Fig. S6). States like Montana and Wyoming also contrast with other West-wide trends; for example, the area burned from lightning-ignited fires accounts for the majority of structure loss in Montana (Fig. S6), and in Wyoming, structure loss rates were not significantly different between ignition sources (Fig. S5).

"High burn–Low loss" regions are epitomized by Idaho, Arizona, and Nevada. These states exhibit varying ways to support widespread fire on landscapes, with low structure loss. These are also states that can safely support and maintain the ecological benefits of wildfire. Regions like Idaho and Nevada have vast areas of federally designated wilderness or otherwise sparsely populated regions, where structure abundance is low. Fire regimes in these two states also vary widely, from climate-sensitive fire regimes in Idaho forests, to fuel-limited fire regimes in Nevada's rangelands. Arizona likewise has large areas of federally designated wilderness, in addition to one of the most active prescribed fire and forest restoration programs in the West (9), and an historical prevalence of low-intensity surface fire regimes. Despite having above-average structure abundance in flammable vegetation (ranking fourth), Arizona had the third lowest structure loss rate in the West (Fig. 4J, 4I, main text).

References

1. K. C. Short, Spatial wildfire occurrence data for the United States, 1992-2018 [FPA_FOD_20210617] (5th Edition) (2021) https:/doi.org/10.2737/RDS-2013-0009.5 (July 6, 2022).
2. V. Iglesias, J. K. Balch, W. R. Travis, US fires became larger, more frequent, and more widespread in the 2000s. *Science Advances* **8** (2022).
3. J. K. Balch, *et al.*, Warming weakens the night-time barrier to global fire. *Nature* **602**, 442–448 (2022).
4. Y. Zhuang, R. Fu, B. D. Santer, R. E. Dickinson, A. Hall, Quantifying contributions of natural variability and anthropogenic forcings on increased fire weather risk over the western United States. *Proceedings of the National Academy of Sciences* **118** (2021).
5. S. A. Parks, J. T. Abatzoglou, Warmer and Drier Fire Seasons Contribute to Increases in Area Burned at High Severity in Western US Forests From 1985 to 2017. *Geophysical Research Letters* **47**, e2020GL089858 (2020).
6. J. T. Abatzoglou, C. A. Kolden, Relationships between climate and macroscale area burned in the western United States. *International Journal of Wildland Fire* **22**, 1003–1020 (2013).
7. J. T. Abatzoglou, A. P. Williams, Impact of anthropogenic climate change on wildfire across western US forests. *Proceedings of the National Academy of Sciences* **113**, 11770–11775 (2016).
8. M. S. Carroll, T. Paveglio, Local Community Agency and Vulnerability Influences on a Montana Wildfire. *Journal of Forestry* **117**, 104–113 (2019).
9. C. A. Kolden, We're Not Doing Enough Prescribed Fire in the Western United States to Mitigate Wildfire Risk. *Fire* **2**, 30 (2019).

Table S1. Wildfire-caused structure loss, relative to the proportion of the total structures in flammable vegetation, at the West-wide scale. The proportion of structures in flammable vegetation destroyed by wildfires increased nearly 3x over the two decades of the analysis. Importantly, this analysis is coarse, because we do not know the precise location of structures, relative to where fires burned. A more spatially precise analysis would reveal the proportion of structure loss, relative to the proportion of structures exposed to wildfire.

Time period	Tot. structures destroyed (#)	Tot. structures in flammable veg. (#)	Proportional loss (# destroyed / # in flammable veg.)
1999-2009	19,066	3,627,879	0.53%
2010-2020	66,014	4,259,774	1.55%

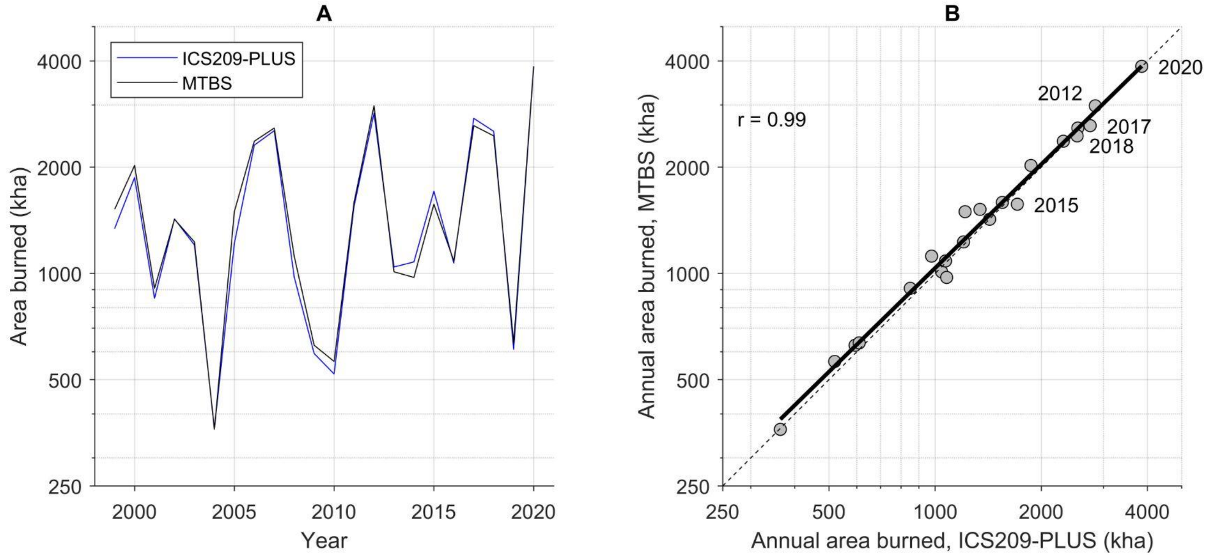

Fig. S1. Comparisons between annual area burned values in the ICS-209-PLUS dataset and Monitoring Trends in Burn Severity (MTBS). (A) Time series, and (B) regression between log(area burned) values, with 1:1 line (dashed line) and best-fit regression line ($r = 0.99$, $p < 0.001$). The MTBS time series was calculated from the total area within all fire perimeters from each year (https://mtbs.gov/).

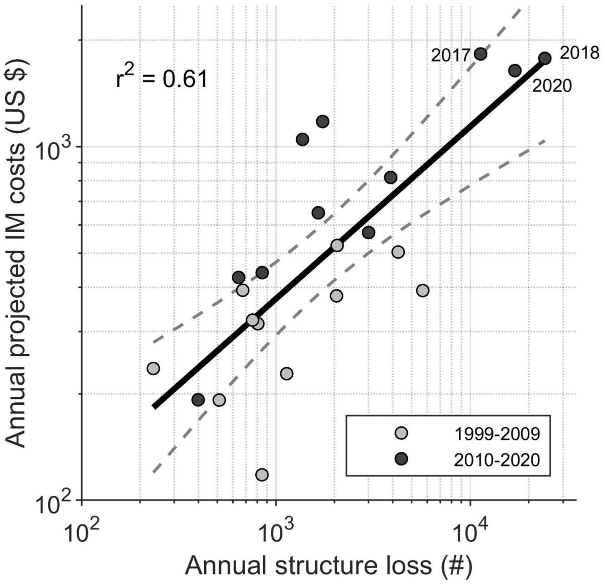

Fig. S2. Annual projected incident management costs are well explained by total annual structure loss across the western United States, 1999-2020. Projected incident management (IM) costs reflect estimates at the end of the incident management phase of a wildfire response, not the final costs of all fire responses or impacts. Variability in total annual structure loss from wildfires explained 61% of the variability in annual projected IM costs (n = 22, $r^2 = 0.61$, F = 31.4, $p < 0.001$).

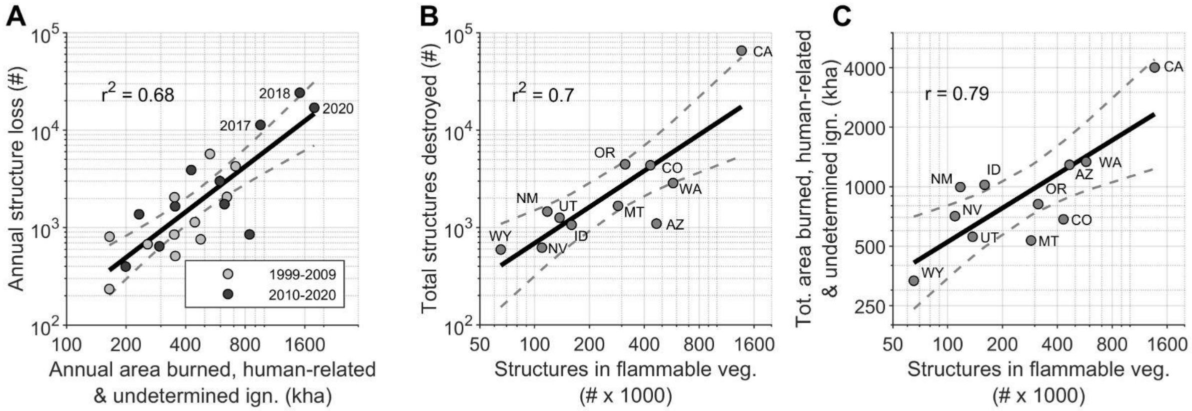

Figure S3. Predictors and correlates of annual and total structure loss from wildfires in the western United States. Same as Fig. 5 in the main text, but with relationships in (A) and (C) using area burned from human-related and undetermined ignitions combined.

Figure S4. State-level temporal patterns of area burned and structure loss from wildfires in the western United States. As in Figure 2 in the main text. Black lines are only shown if the annual total of the variable displayed in bars is correlated with VPD (A) or area burned (B-C).

BELOW

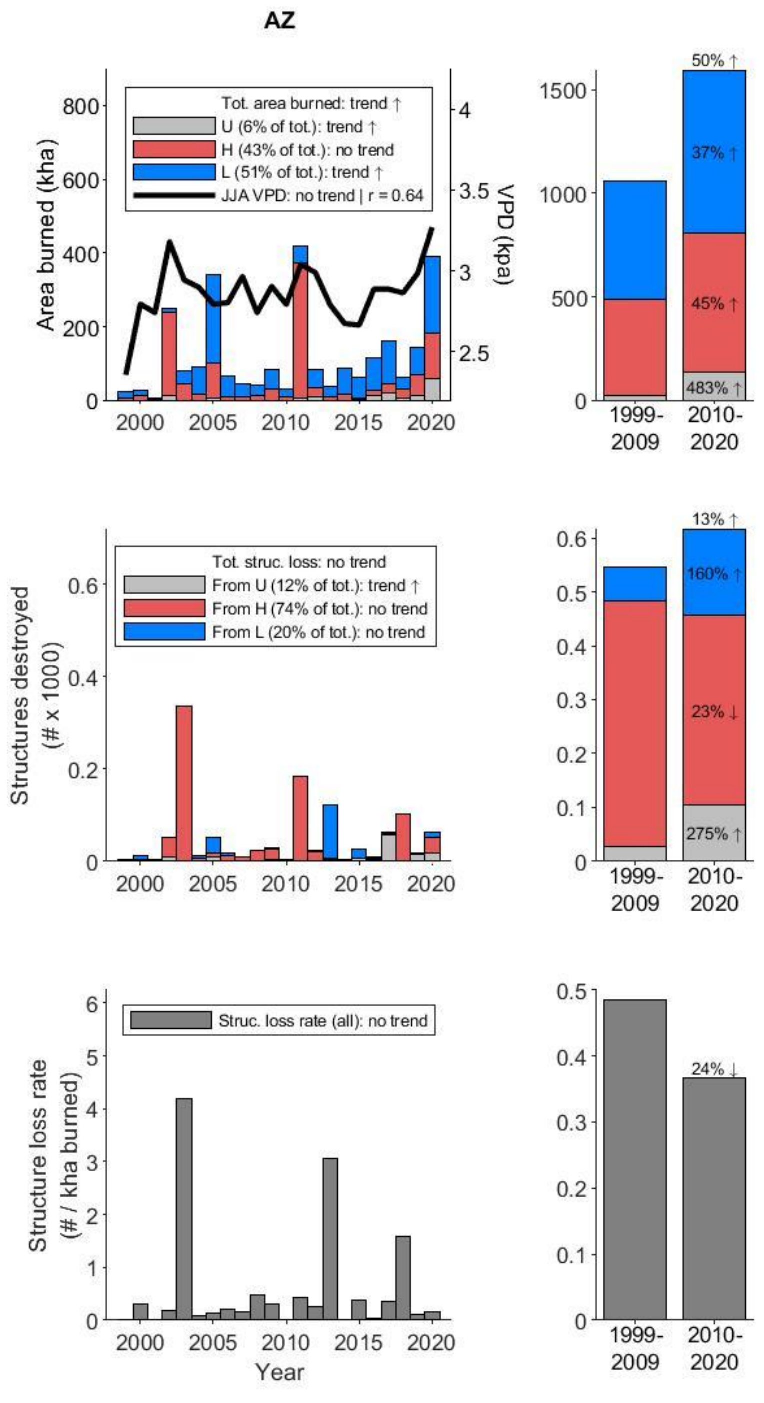
AZ
Tot. area burned: trend ↑
U (6% of tot.): trend ↑
H (43% of tot.): no trend
L (51% of tot.): trend ↑
JJA VPD: no trend | r = 0.64
Area burned (kha)
VPD (kpa)
50% ↑
37% ↑
45% ↑
483% ↑
Tot. struc. loss: no trend
From U (12% of tot.): trend ↑
From H (74% of tot.): no trend
From L (20% of tot.): no trend
Structures destroyed (# x 1000)
13% ↑
160% ↑
23% ↓
275% ↑
Struc. loss rate (all): no trend
Structure loss rate (# / kha burned)
24% ↓
Year
1999-2009
2010-2020

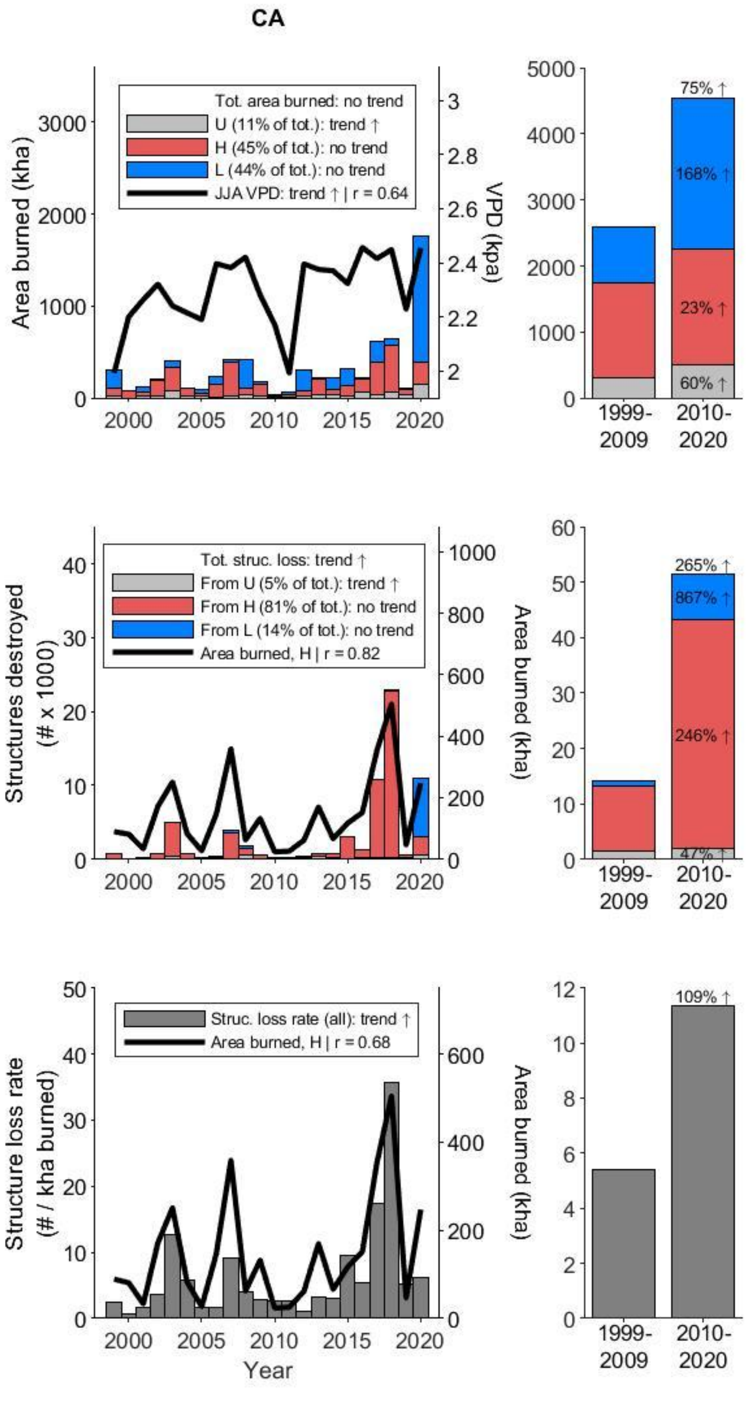
CA
Tot. area burned: no trend
U (11% of tot.): trend ↑
H (45% of tot.): no trend
L (44% of tot.): no trend
JJA VPD: trend ↑ | r = 0.64
Area burned (kha)
VPD (kpa)
75% ↑
168% ↑
23% ↑
60% ↑
1999-2009
2010-2020
Tot. struc. loss: trend ↑
From U (5% of tot.): trend ↑
From H (81% of tot.): no trend
From L (14% of tot.): no trend
Area burned, H | r = 0.82
Structures destroyed (# x 1000)
Area burned (kha)
265% ↑
867% ↑
246% ↑
47% ↑
Struc. loss rate (all): trend ↑
Area burned, H | r = 0.68
Structure loss rate (# / kha burned)
Area burned (kha)
109% ↑
Year

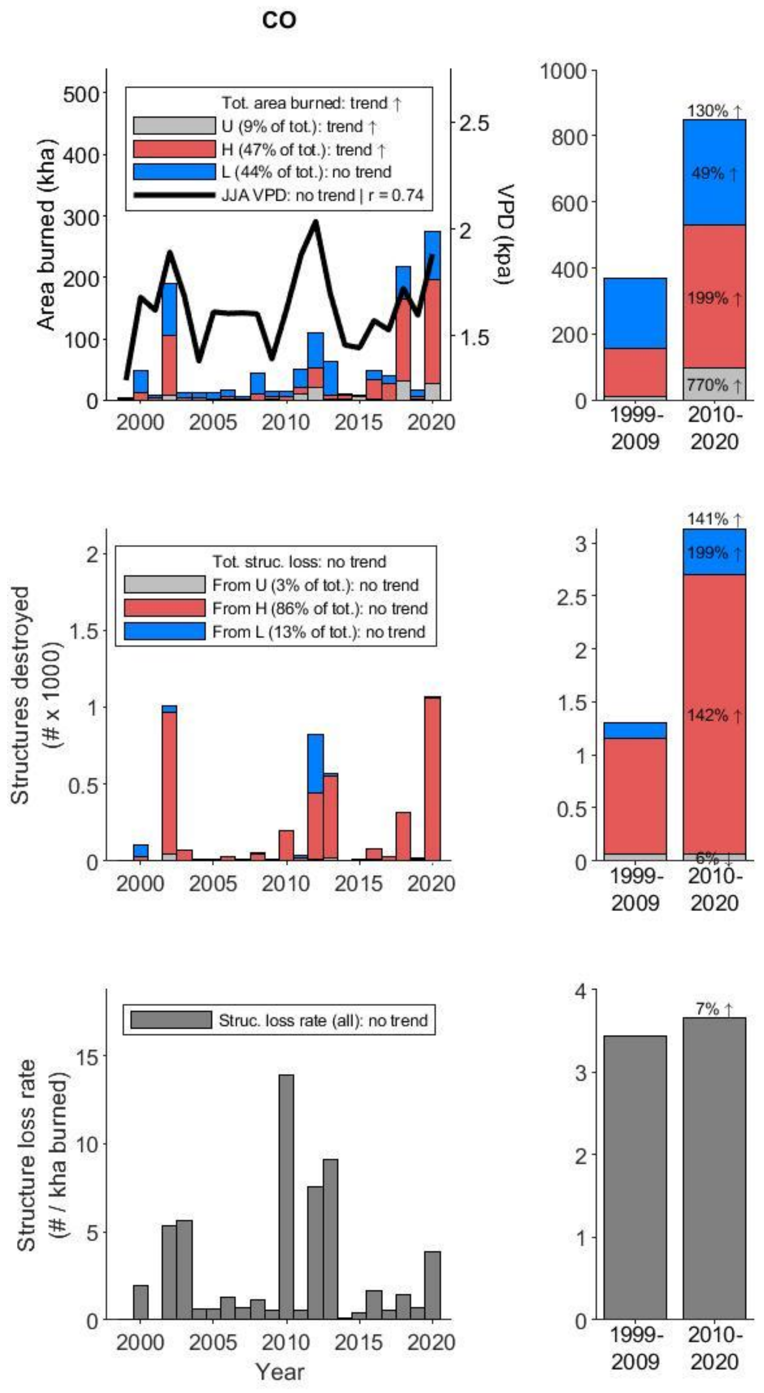
CO
Tot. area burned: trend ↑
U (9% of tot.): trend ↑
H (47% of tot.): trend ↑
L (44% of tot.): no trend
JJA VPD: no trend | r = 0.74
Area burned (kha)
VPD (kpa)
130% ↑
49% ↑
199% ↑
770% ↑
1999-2009
2010-2020
Tot. struc. loss: no trend
From U (3% of tot.): no trend
From H (86% of tot.): no trend
From L (13% of tot.): no trend
Structures destroyed (# x 1000)
141% ↑
199% ↑
142% ↑
6% ↓
Struc. loss rate (all): no trend
Structure loss rate (# / kha burned)
7% ↑
Year

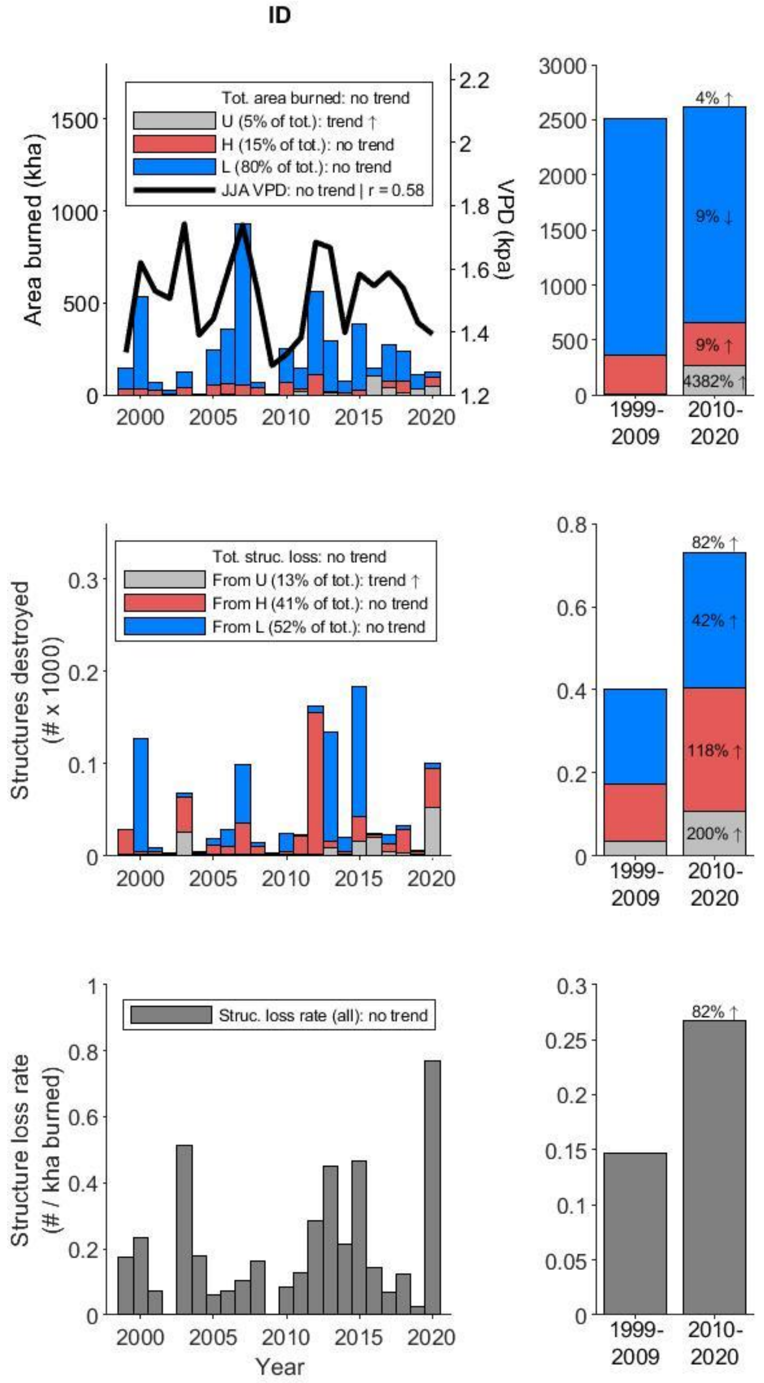
ID
Tot. area burned: no trend
U (5% of tot.): trend ↑
H (15% of tot.): no trend
L (80% of tot.): no trend
JJA VPD: no trend | r = 0.58
Area burned (kha)
VPD (kpa)
4% ↑
9% ↓
9% ↑
4382% ↑
Tot. struc. loss: no trend
From U (13% of tot.): trend ↑
From H (41% of tot.): no trend
From L (52% of tot.): no trend
Structures destroyed (# x 1000)
82% ↑
42% ↑
118% ↑
200% ↑
Struc. loss rate (all): no trend
Structure loss rate (# / kha burned)
82% ↑
Year
1999-2009
2010-2020

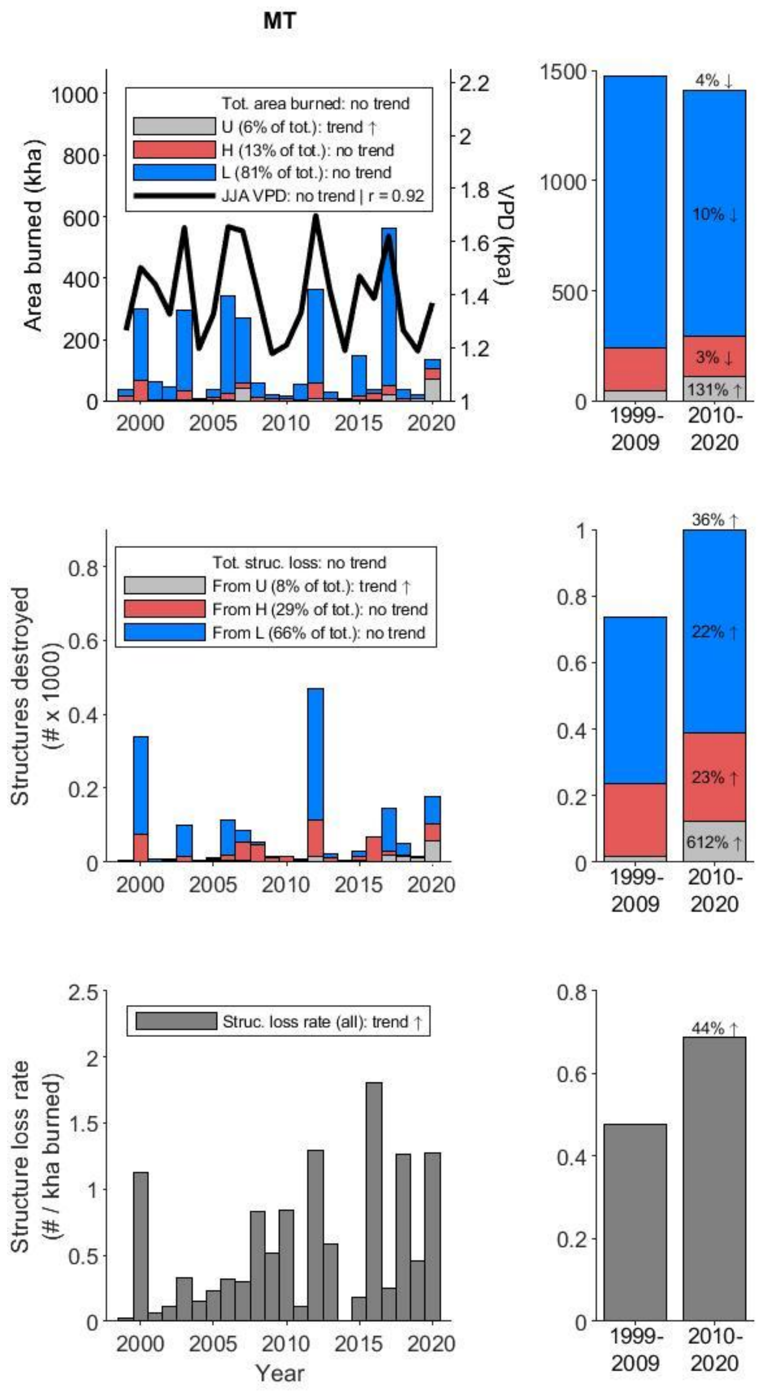
MT
Tot. area burned: no trend
U (6% of tot.): trend ↑
H (13% of tot.): no trend
L (81% of tot.): no trend
JJA VPD: no trend | r = 0.92
Area burned (kha)
VPD (kpa)
4% ↓
10% ↓
3% ↓
131% ↑
1999-2009
2010-2020
Tot. struc. loss: no trend
From U (8% of tot.): trend ↑
From H (29% of tot.): no trend
From L (66% of tot.): no trend
Structures destroyed (# x 1000)
36% ↑
22% ↑
23% ↑
612% ↑
Struc. loss rate (all): trend ↑
Structure loss rate (# / kha burned)
44% ↑
Year

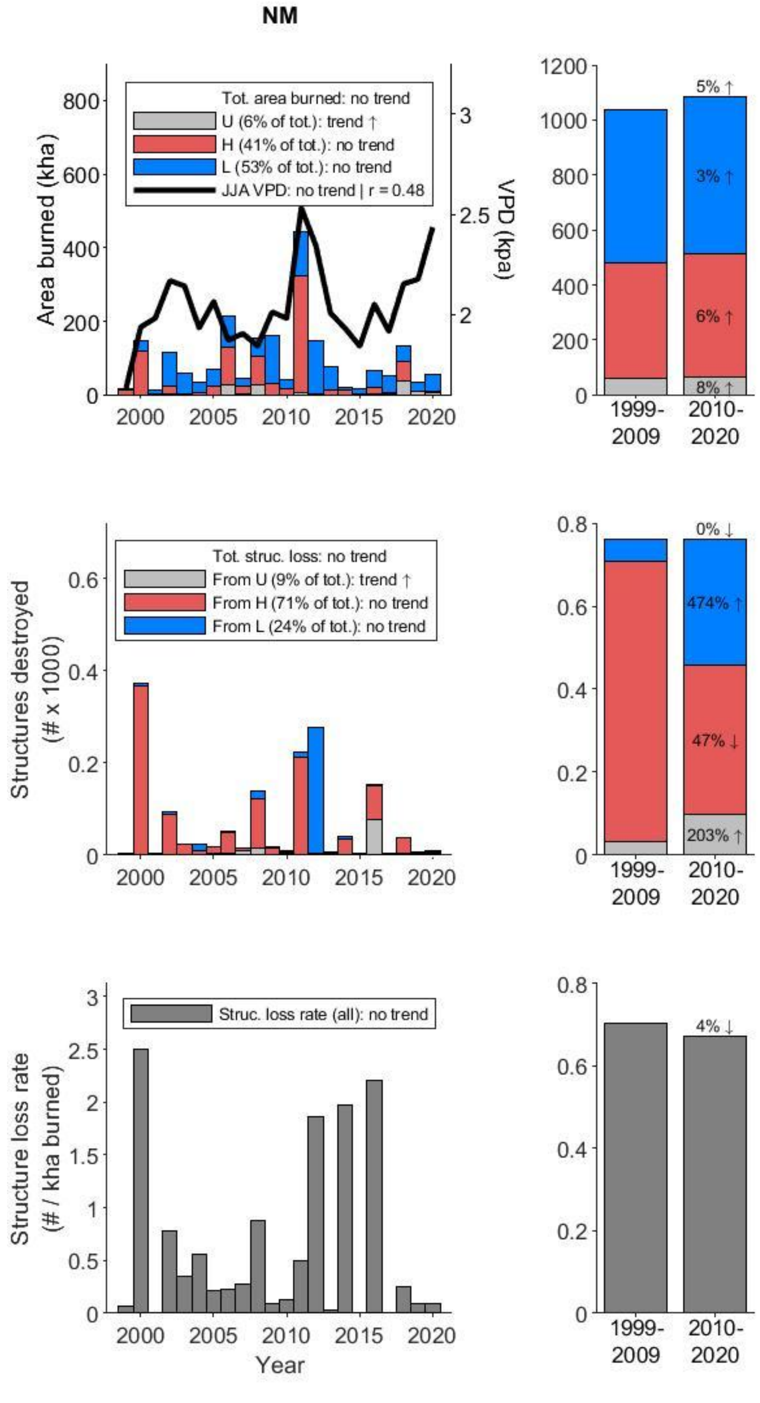
NM
Tot. area burned: no trend
U (6% of tot.): trend ↑
H (41% of tot.): no trend
L (53% of tot.): no trend
JJA VPD: no trend | r = 0.48
Area burned (kha)
VPD (kpa)
5% ↑
3% ↑
6% ↑
8% ↑
1999-2009
2010-2020
Tot. struc. loss: no trend
From U (9% of tot.): trend ↑
From H (71% of tot.): no trend
From L (24% of tot.): no trend
Structures destroyed (# x 1000)
0% ↓
474% ↑
47% ↓
203% ↑
Struc. loss rate (all): no trend
Structure loss rate (# / kha burned)
4% ↓
Year

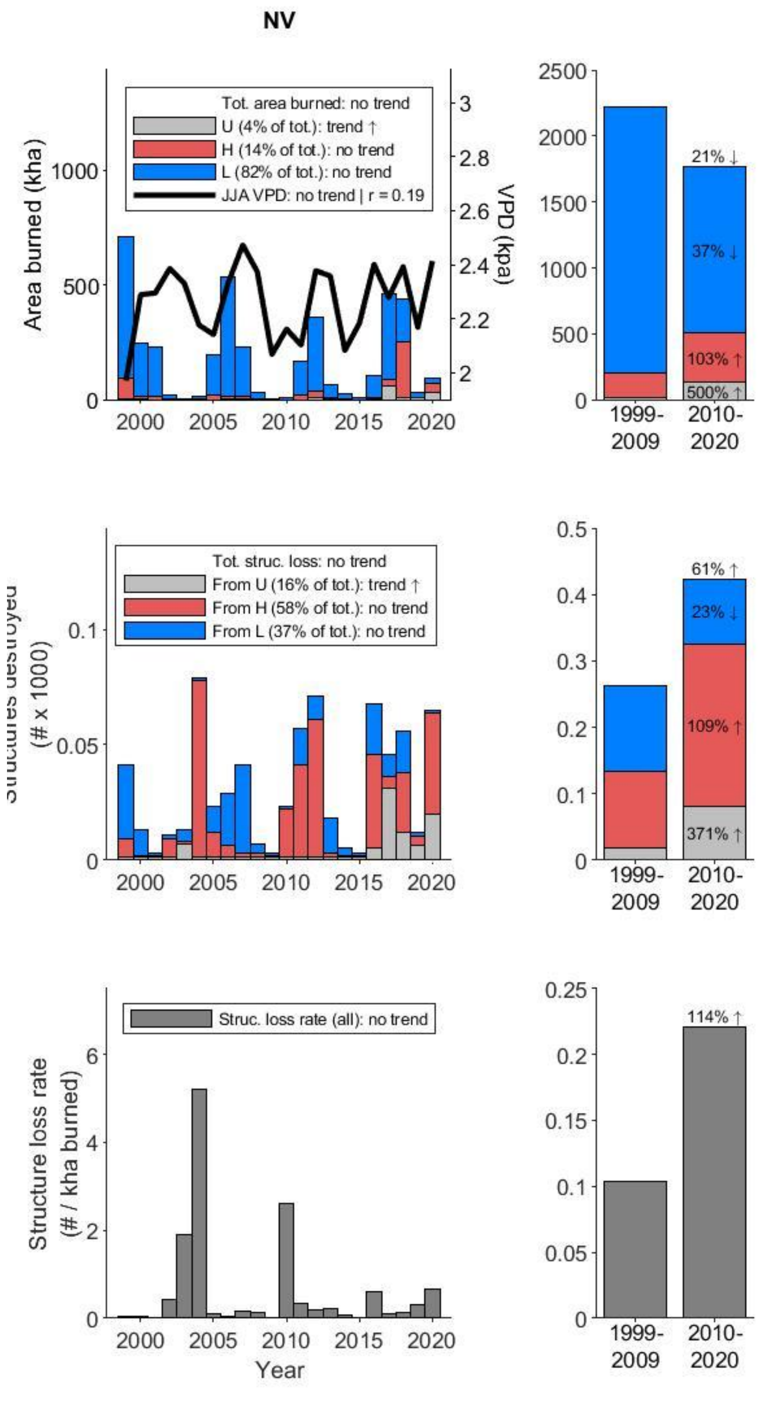
NV
Tot. area burned: no trend
U (4% of tot.): trend ↑
H (14% of tot.): no trend
L (82% of tot.): no trend
JJA VPD: no trend | r = 0.19
Area burned (kha)
VPD (kpa)
21% ↓
37% ↓
103% ↑
500% ↑
1999-2009
2010-2020
Tot. struc. loss: no trend
From U (16% of tot.): trend ↑
From H (58% of tot.): no trend
From L (37% of tot.): no trend
Structures destroyed (# x 1000)
61% ↑
23% ↓
109% ↑
371% ↑
Struc. loss rate (all): no trend
Structure loss rate (# / kha burned)
114% ↑
Year

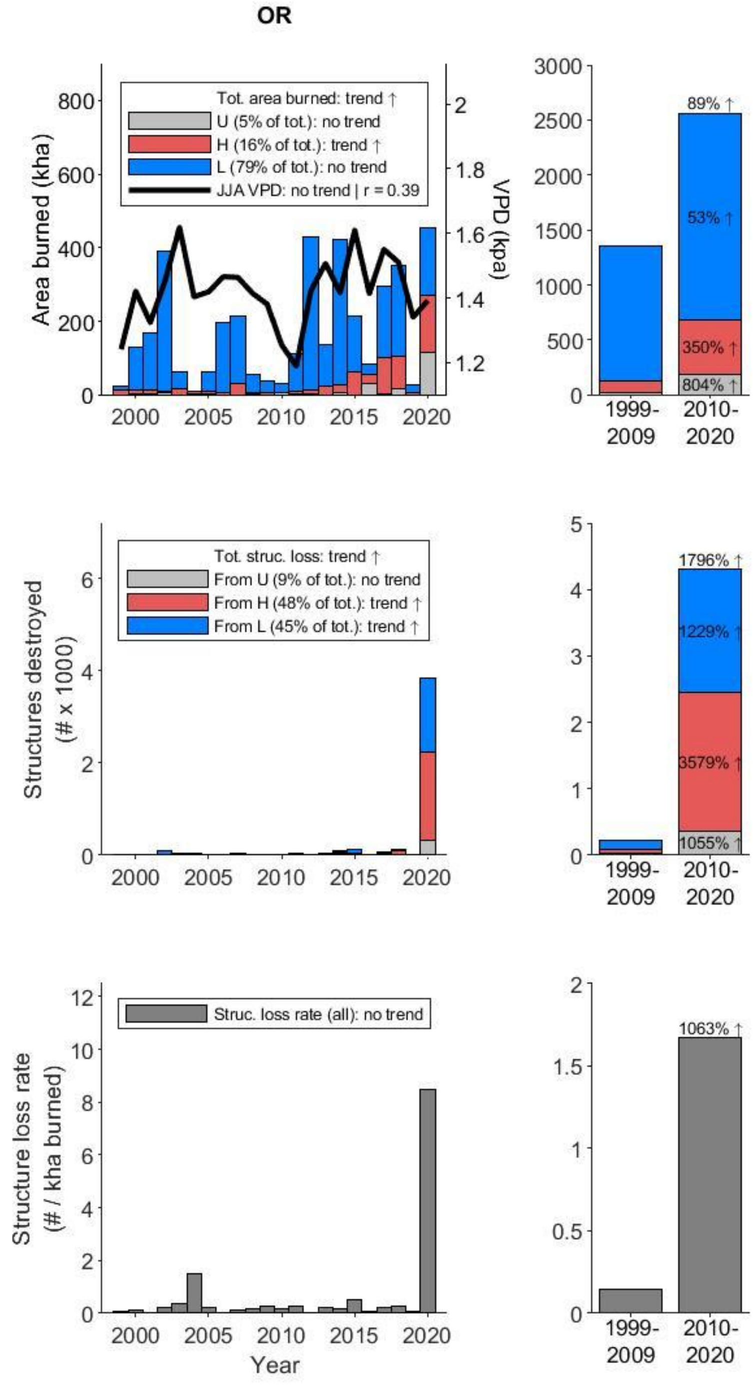
OR
Tot. area burned: trend ↑
U (5% of tot.): no trend
H (16% of tot.): trend ↑
L (79% of tot.): no trend
JJA VPD: no trend | r = 0.39
Area burned (kha)
VPD (kpa)
89% ↑
53% ↑
350% ↑
804% ↑
1999-2009
2010-2020
Tot. struc. loss: trend ↑
From U (9% of tot.): no trend
From H (48% of tot.): trend ↑
From L (45% of tot.): trend ↑
Structures destroyed (# x 1000)
1796% ↑
1229% ↑
3579% ↑
1055% ↑
Struc. loss rate (all): no trend
Structure loss rate (# / kha burned)
1063% ↑
Year

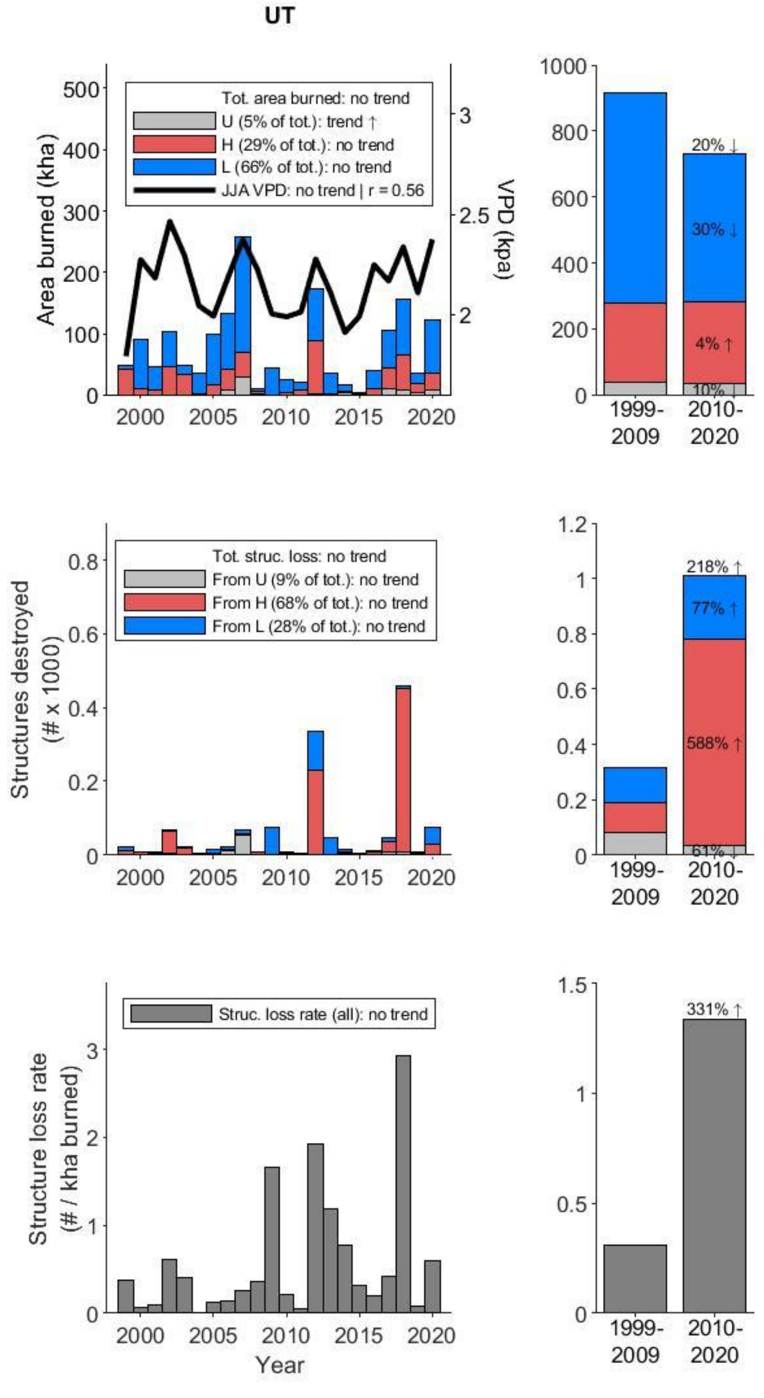
UT
Tot. area burned: no trend
U (5% of tot.): trend ↑
H (29% of tot.): no trend
L (66% of tot.): no trend
JJA VPD: no trend | r = 0.56
Area burned (kha)
VPD (kpa)
20% ↓
30% ↓
4% ↑
10% ↓
1999-2009
2010-2020
Tot. struc. loss: no trend
From U (9% of tot.): no trend
From H (68% of tot.): no trend
From L (28% of tot.): no trend
Structures destroyed (# x 1000)
218% ↑
77% ↑
588% ↑
61% ↓
Struc. loss rate (all): no trend
Structure loss rate (# / kha burned)
331% ↑
Year

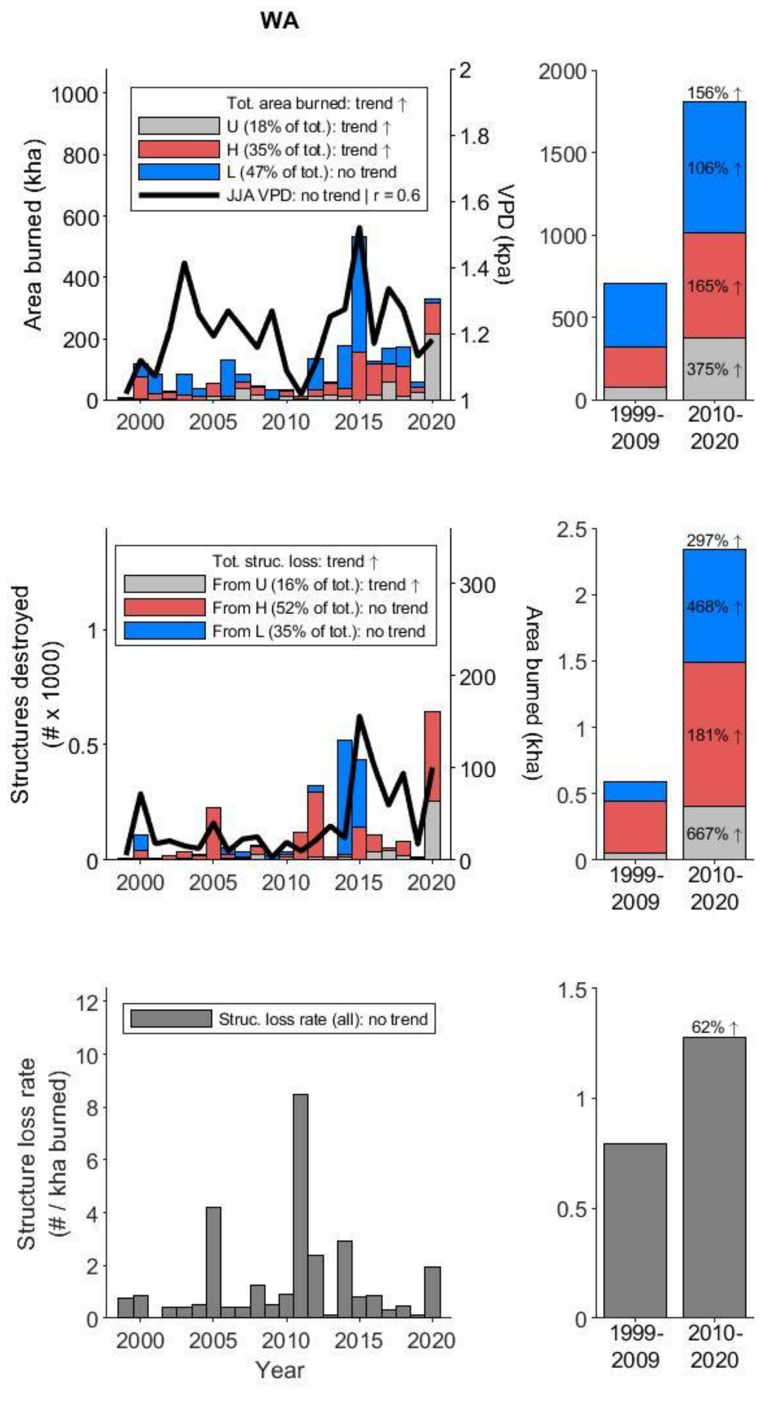
WA
Tot. area burned: trend ↑
U (18% of tot.): trend ↑
H (35% of tot.): trend ↑
L (47% of tot.): no trend
JJA VPD: no trend | r = 0.6
Area burned (kha)
VPD (kpa)
156% ↑
106% ↑
165% ↑
375% ↑
1999-2009
2010-2020
Tot. struc. loss: trend ↑
From U (16% of tot.): trend ↑
From H (52% of tot.): no trend
From L (35% of tot.): no trend
Structures destroyed (# x 1000)
Area burned (kha)
297% ↑
468% ↑
181% ↑
667% ↑
Struc. loss rate (all): no trend
Structure loss rate (# / kha burned)
62% ↑
Year

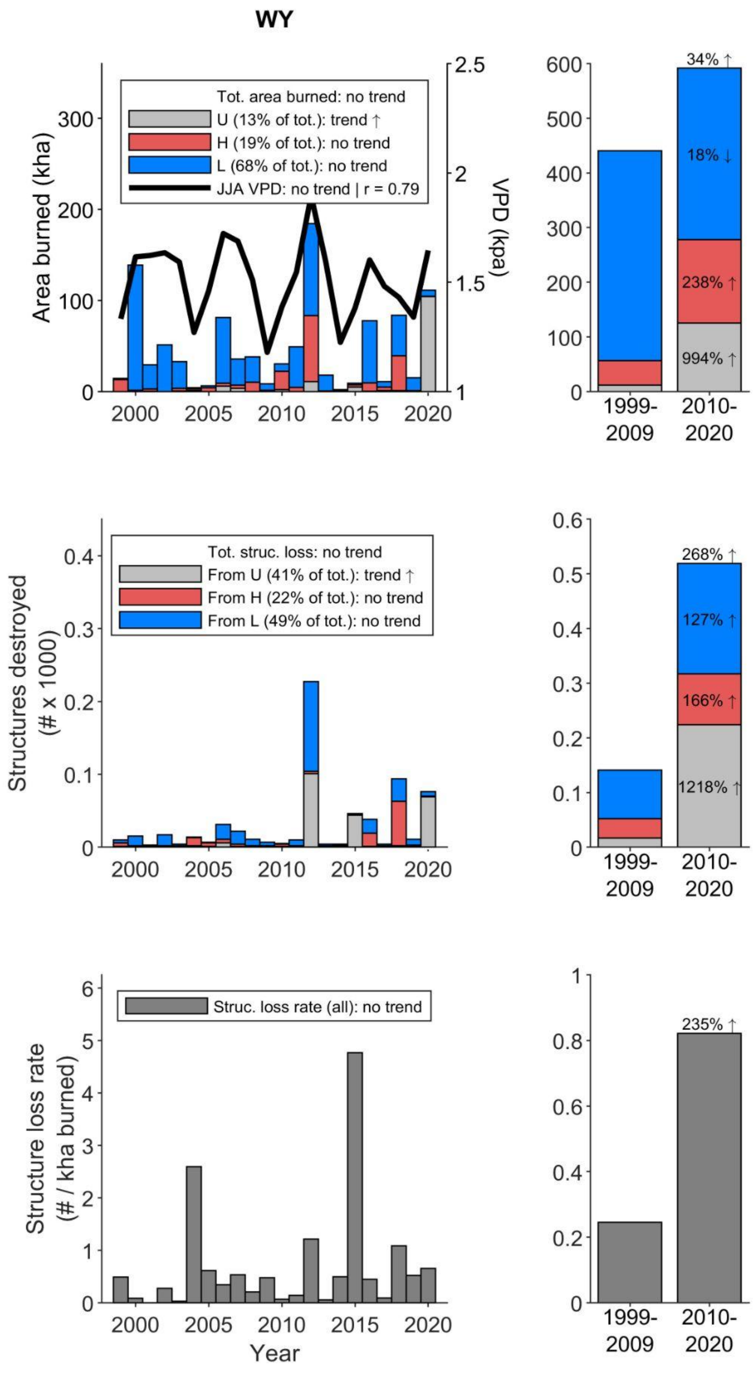
WY
Tot. area burned: no trend
U (13% of tot.): trend ↑
H (19% of tot.): no trend
L (68% of tot.): no trend
JJA VPD: no trend | r = 0.79
Area burned (kha)
VPD (kpa)
34% ↑
18% ↓
238% ↑
994% ↑
Tot. struc. loss: no trend
From U (41% of tot.): trend ↑
From H (22% of tot.): no trend
From L (49% of tot.): no trend
Structures destroyed (# x 1000)
268% ↑
127% ↑
166% ↑
1218% ↑
Struc. loss rate (all): no trend
Structure loss rate (# / kha burned)
235% ↑
Year
1999-2009
2010-2020

Figure S5. State-level fire regime attributes by ignition source for the western United States. As in Figure 3 in the main text. Non-significant between-median values are labeled with "Medians not sig. diff." if the Wilcoxon rank-sum tests yielded a p-value > 0.10. In main-text Fig. 3, the legend across the bottom row, not included here, is identical.

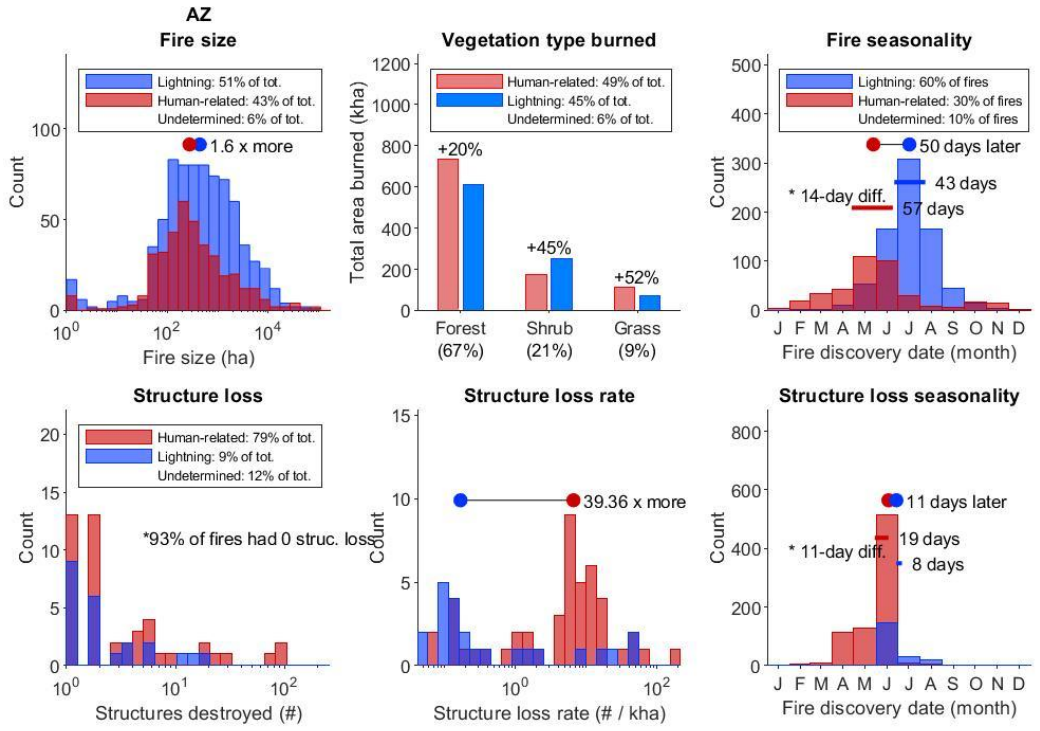

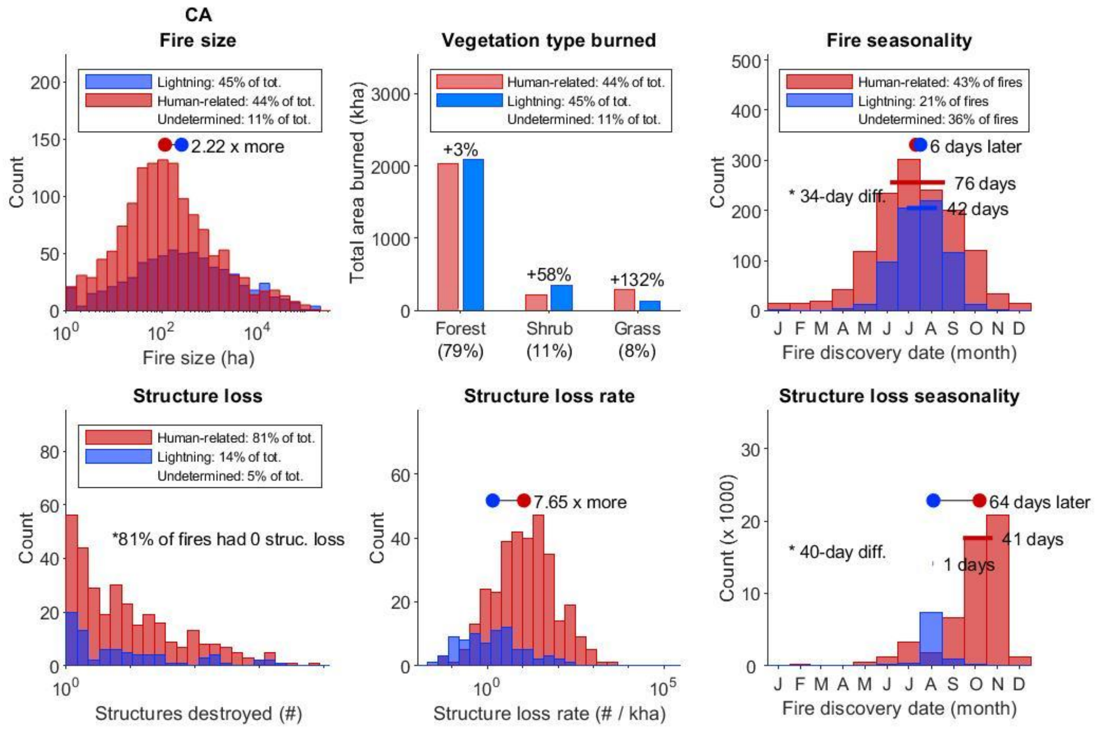
CA
Fire size
Lightning: 45% of tot.
Human-related: 44% of tot.
Undetermined: 11% of tot.
2.22 x more
Count
Fire size (ha)
Vegetation type burned
Human-related: 44% of tot.
Lightning: 45% of tot.
Undetermined: 11% of tot.
+3%
+58%
+132%
Total area burned (kha)
Forest (79%)
Shrub (11%)
Grass (8%)
Fire seasonality
Human-related: 43% of fires
Lightning: 21% of fires
Undetermined: 36% of fires
6 days later
76 days
42 days
* 34-day diff.
Count
J F M A M J J A S O N D
Fire discovery date (month)
Structure loss
Human-related: 81% of tot.
Lightning: 14% of tot.
Undetermined: 5% of tot.
*81% of fires had 0 struc. loss
Count
Structures destroyed (#)
Structure loss rate
7.65 x more
Count
Structure loss rate (# / kha)
Structure loss seasonality
64 days later
41 days
1 days
* 40-day diff.
Count (x 1000)
J F M A M J J A S O N D
Fire discovery date (month)

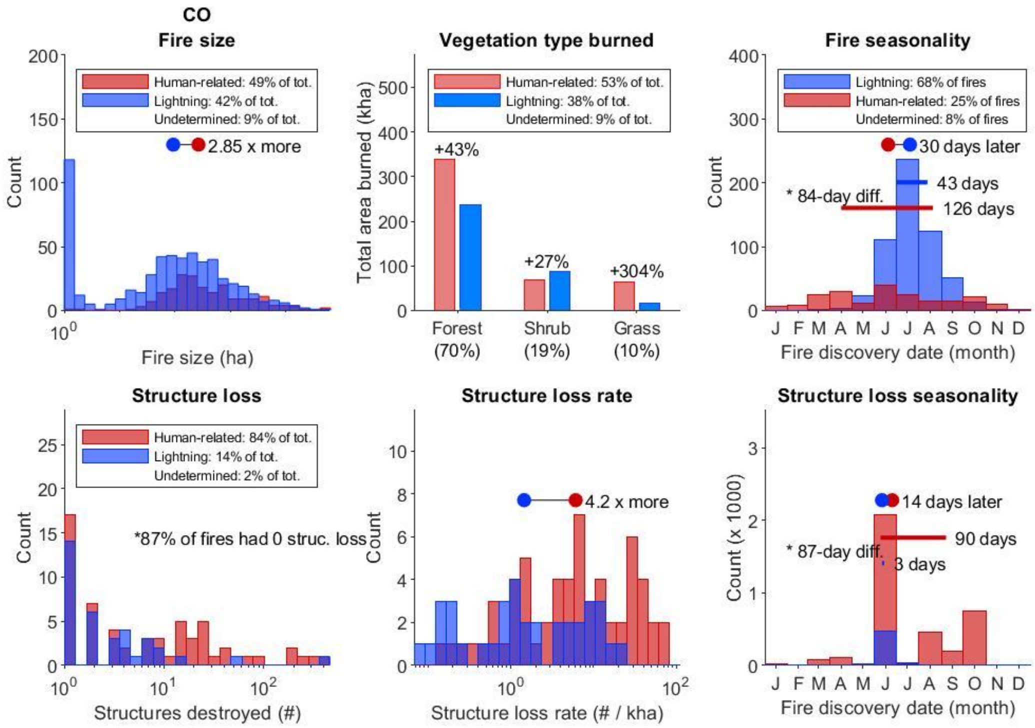
CO
Fire size
Human-related: 49% of tot.
Lightning: 42% of tot.
Undetermined: 9% of tot.
2.85 x more
Count
Fire size (ha)
Vegetation type burned
Human-related: 53% of tot.
Lightning: 38% of tot.
Undetermined: 9% of tot.
+43%
+27%
+304%
Total area burned (kha)
Forest (70%)
Shrub (19%)
Grass (10%)
Fire seasonality
Lightning: 68% of fires
Human-related: 25% of fires
Undetermined: 8% of fires
30 days later
43 days
* 84-day diff.
126 days
Count
J F M A M J J A S O N D
Fire discovery date (month)
Structure loss
Human-related: 84% of tot.
Lightning: 14% of tot.
Undetermined: 2% of tot.
*87% of fires had 0 struc. loss
Count
Structures destroyed (#)
Structure loss rate
4.2 x more
Count
Structure loss rate (# / kha)
Structure loss seasonality
14 days later
90 days
* 87-day diff.
3 days
Count (x 1000)
J F M A M J J A S O N D
Fire discovery date (month)

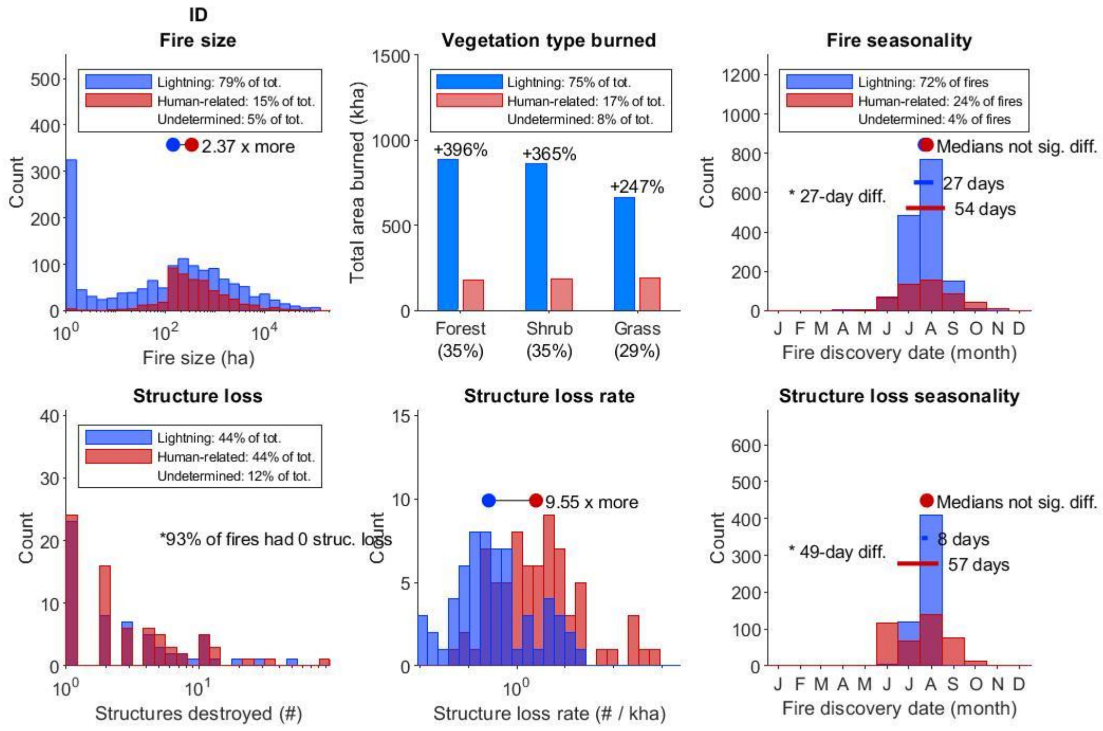

ID
Fire size
Lightning: 79% of tot.
Human-related: 15% of tot.
Undetermined: 5% of tot.
2.37 x more
Count
Fire size (ha)
Vegetation type burned
Lightning: 75% of tot.
Human-related: 17% of tot.
Undetermined: 8% of tot.
+396%
+365%
+247%
Total area burned (kha)
Forest (35%)
Shrub (35%)
Grass (29%)
Fire seasonality
Lightning: 72% of fires
Human-related: 24% of fires
Undetermined: 4% of fires
Medians not sig. diff.
* 27-day diff.
27 days
54 days
Fire discovery date (month)
Structure loss
Lightning: 44% of tot.
Human-related: 44% of tot.
Undetermined: 12% of tot.
*93% of fires had 0 struc. loss
Structures destroyed (#)
Structure loss rate
9.55 x more
Structure loss rate (# / kha)
Structure loss seasonality
Medians not sig. diff.
* 49-day diff.
8 days
57 days
Fire discovery date (month)

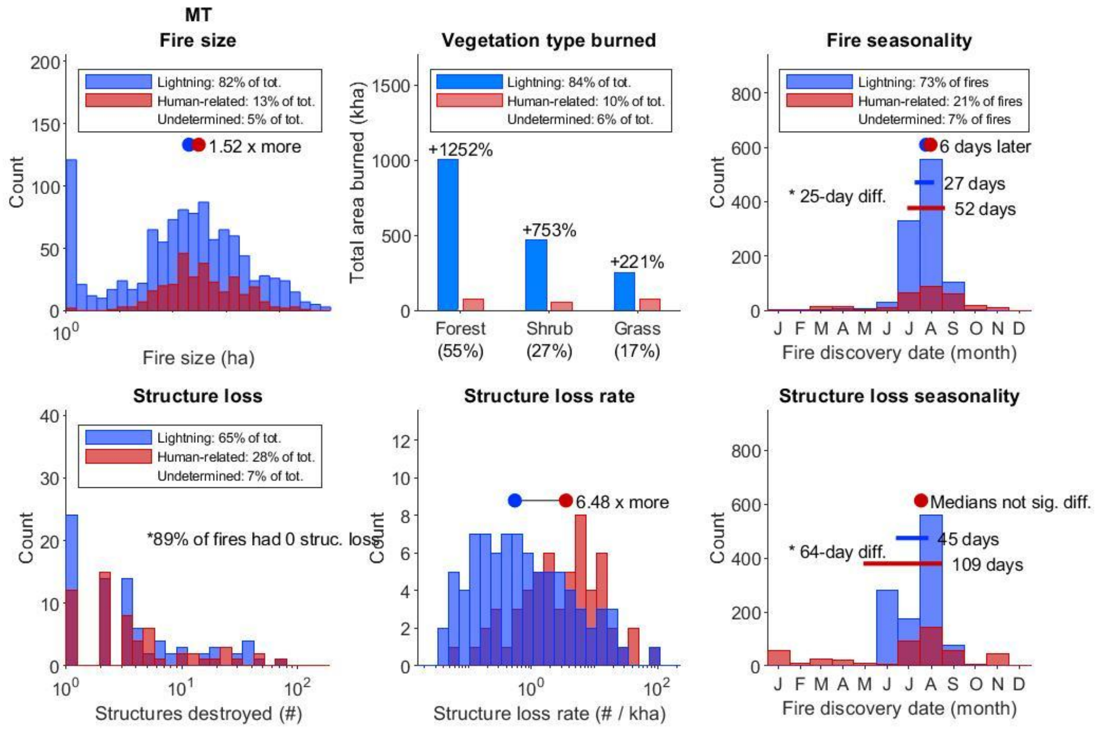
MT
Fire size
Lightning: 82% of tot.
Human-related: 13% of tot.
Undetermined: 5% of tot.
1.52 x more
Count
Fire size (ha)
Vegetation type burned
Lightning: 84% of tot.
Human-related: 10% of tot.
Undetermined: 6% of tot.
Total area burned (kha)
+1252%
+753%
+221%
Forest (55%)
Shrub (27%)
Grass (17%)
Fire seasonality
Lightning: 73% of fires
Human-related: 21% of fires
Undetermined: 7% of fires
6 days later
27 days
52 days
* 25-day diff.
Fire discovery date (month)
Structure loss
Lightning: 65% of tot.
Human-related: 28% of tot.
Undetermined: 7% of tot.
*89% of fires had 0 struc. loss
Structures destroyed (#)
Structure loss rate
6.48 x more
Structure loss rate (# / kha)
Structure loss seasonality
Medians not sig. diff.
45 days
109 days
* 64-day diff.
Fire discovery date (month)

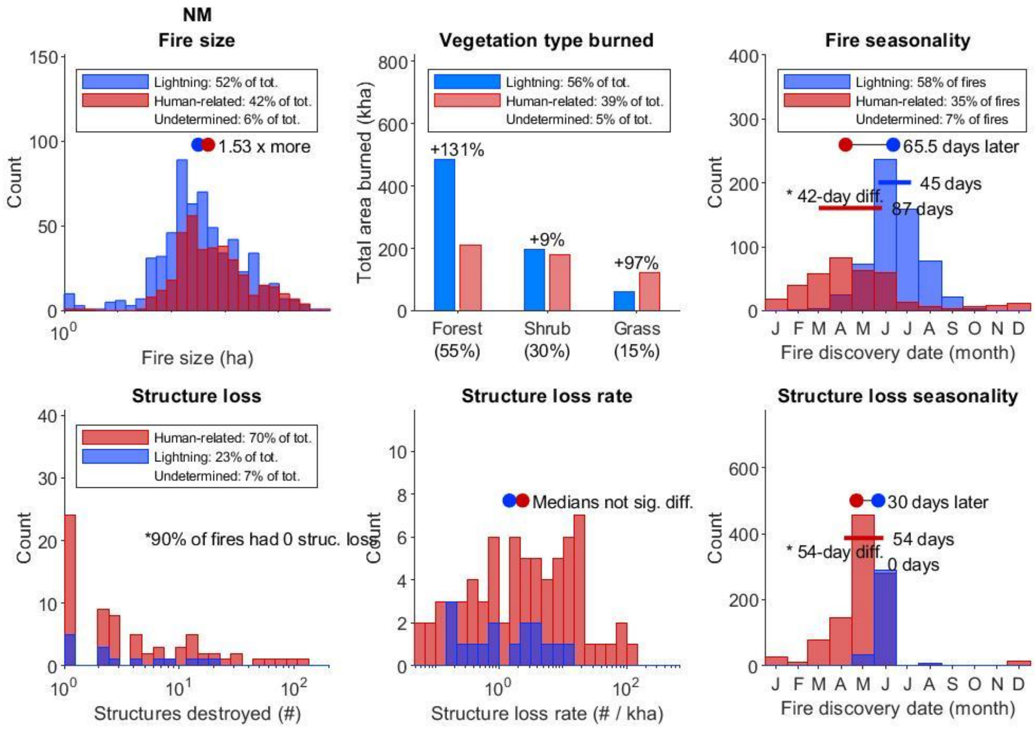
NM
Fire size
Lightning: 52% of tot.
Human-related: 42% of tot.
Undetermined: 6% of tot.
1.53 x more
Count
Fire size (ha)
Vegetation type burned
Lightning: 56% of tot.
Human-related: 39% of tot.
Undetermined: 5% of tot.
Total area burned (kha)
+131%
+9%
+97%
Forest (55%)
Shrub (30%)
Grass (15%)
Fire seasonality
Lightning: 58% of fires
Human-related: 35% of fires
Undetermined: 7% of fires
65.5 days later
45 days
* 42-day diff.
87 days
Fire discovery date (month)
Structure loss
Human-related: 70% of tot.
Lightning: 23% of tot.
Undetermined: 7% of tot.
*90% of fires had 0 struc. loss
Structures destroyed (#)
Structure loss rate
Medians not sig. diff.
Structure loss rate (# / kha)
Structure loss seasonality
30 days later
54 days
* 54-day diff.
0 days
Fire discovery date (month)

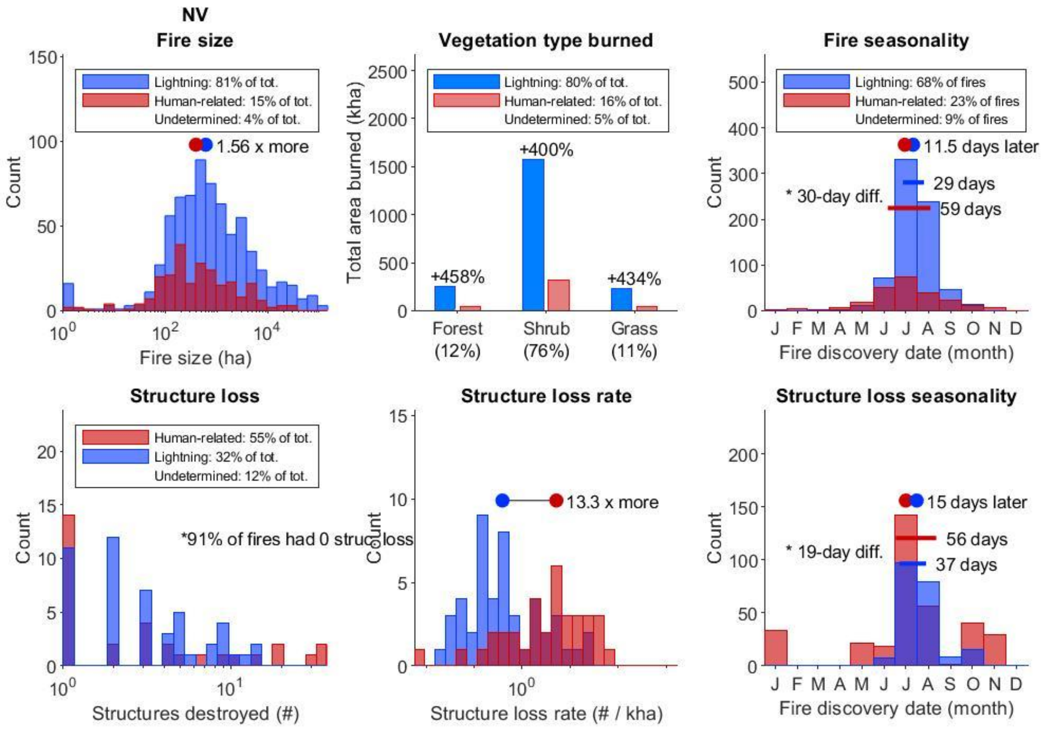
NV
Fire size
Lightning: 81% of tot.
Human-related: 15% of tot.
Undetermined: 4% of tot.
1.56 x more
Count
Fire size (ha)
Vegetation type burned
Lightning: 80% of tot.
Human-related: 16% of tot.
Undetermined: 5% of tot.
Total area burned (kha)
+458%
+400%
+434%
Forest (12%)
Shrub (76%)
Grass (11%)
Fire seasonality
Lightning: 68% of fires
Human-related: 23% of fires
Undetermined: 9% of fires
11.5 days later
29 days
59 days
* 30-day diff.
Fire discovery date (month)
Structure loss
Human-related: 55% of tot.
Lightning: 32% of tot.
Undetermined: 12% of tot.
*91% of fires had 0 struc. loss
Structures destroyed (#)
Structure loss rate
13.3 x more
Structure loss rate (# / kha)
Structure loss seasonality
15 days later
56 days
37 days
* 19-day diff.
Fire discovery date (month)

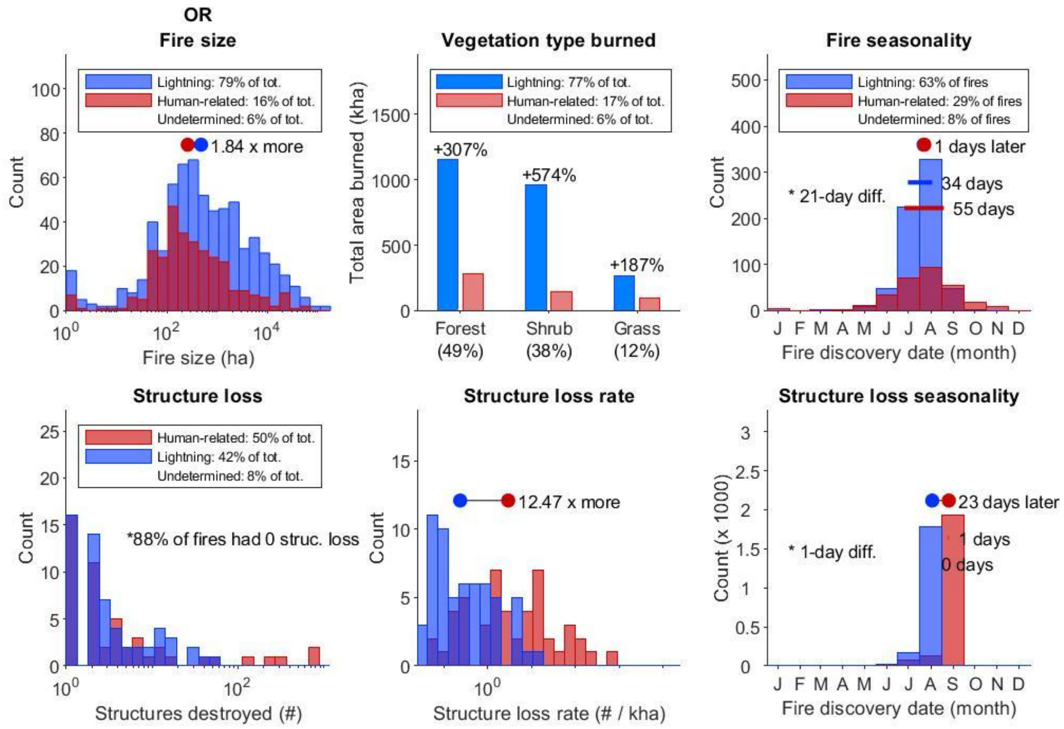
OR
Fire size
Lightning: 79% of tot.
Human-related: 16% of tot.
Undetermined: 6% of tot.
1.84 x more
Count
Fire size (ha)
Vegetation type burned
Lightning: 77% of tot.
Human-related: 17% of tot.
Undetermined: 6% of tot.
+307%
+574%
+187%
Total area burned (kha)
Forest (49%)
Shrub (38%)
Grass (12%)
Fire seasonality
Lightning: 63% of fires
Human-related: 29% of fires
Undetermined: 8% of fires
1 days later
34 days
55 days
* 21-day diff.
Count
Fire discovery date (month)
Structure loss
Human-related: 50% of tot.
Lightning: 42% of tot.
Undetermined: 8% of tot.
*88% of fires had 0 struc. loss
Count
Structures destroyed (#)
Structure loss rate
12.47 x more
Count
Structure loss rate (# / kha)
Structure loss seasonality
23 days later
1 days
0 days
* 1-day diff.
Count (x 1000)
Fire discovery date (month)

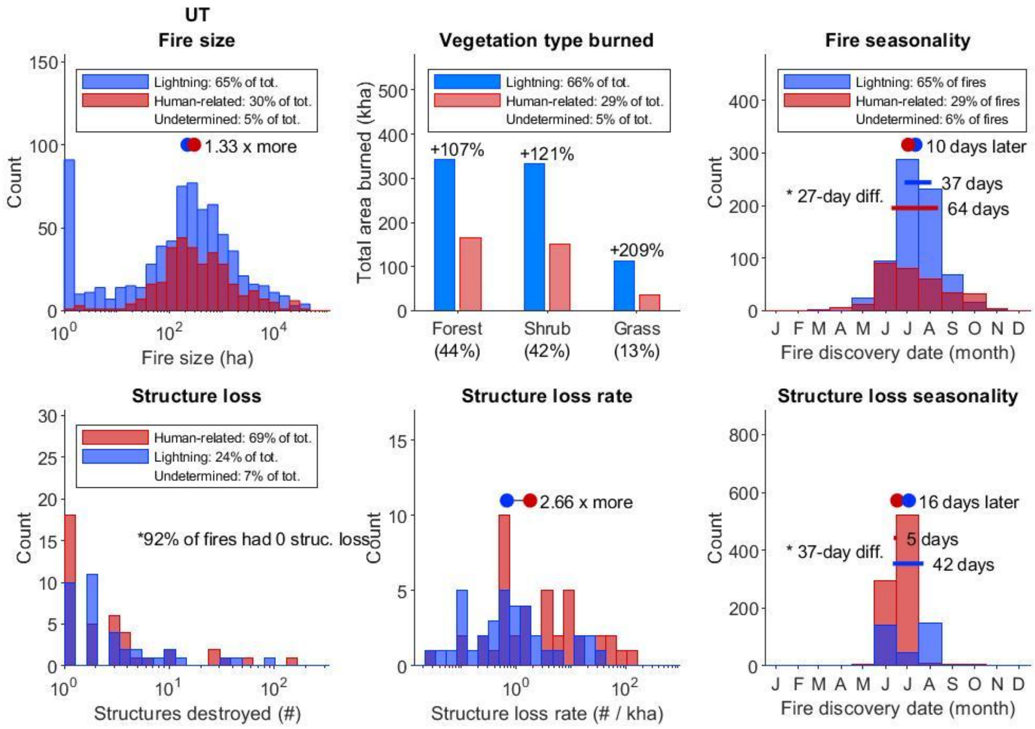
UT
Fire size
Lightning: 65% of tot.
Human-related: 30% of tot.
Undetermined: 5% of tot.
1.33 x more
Count
Fire size (ha)
Vegetation type burned
Lightning: 66% of tot.
Human-related: 29% of tot.
Undetermined: 5% of tot.
+107%
+121%
+209%
Total area burned (kha)
Forest (44%)
Shrub (42%)
Grass (13%)
Fire seasonality
Lightning: 65% of fires
Human-related: 29% of fires
Undetermined: 6% of fires
10 days later
37 days
64 days
* 27-day diff.
Fire discovery date (month)
Structure loss
Human-related: 69% of tot.
Lightning: 24% of tot.
Undetermined: 7% of tot.
*92% of fires had 0 struc. loss
Structures destroyed (#)
Structure loss rate
2.66 x more
Structure loss rate (# / kha)
Structure loss seasonality
16 days later
5 days
42 days
* 37-day diff.
Fire discovery date (month)

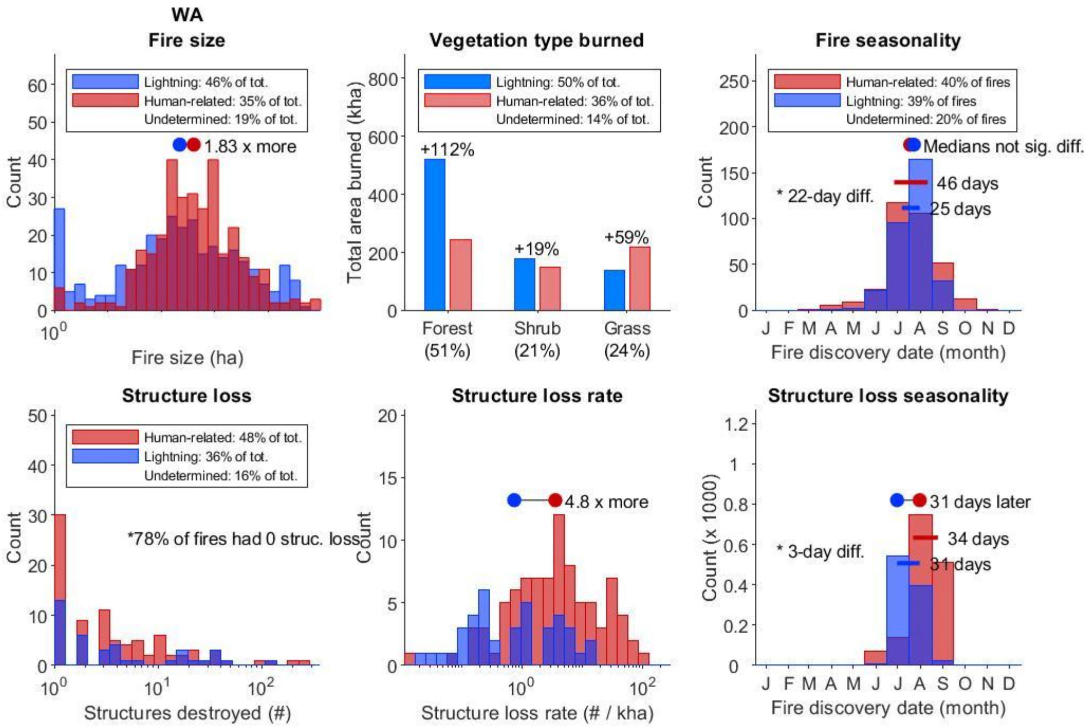
WA
Fire size
Lightning: 46% of tot.
Human-related: 35% of tot.
Undetermined: 19% of tot.
1.83 x more
Count
Fire size (ha)
Vegetation type burned
Lightning: 50% of tot.
Human-related: 36% of tot.
Undetermined: 14% of tot.
+112%
+19%
+59%
Total area burned (kha)
Forest (51%)
Shrub (21%)
Grass (24%)
Fire seasonality
Human-related: 40% of fires
Lightning: 39% of fires
Undetermined: 20% of fires
Medians not sig. diff.
* 22-day diff.
46 days
25 days
Fire discovery date (month)
Structure loss
Human-related: 48% of tot.
Lightning: 36% of tot.
Undetermined: 16% of tot.
*78% of fires had 0 struc. loss
Structures destroyed (#)
Structure loss rate
4.8 x more
Structure loss rate (# / kha)
Structure loss seasonality
31 days later
* 3-day diff.
34 days
31 days
Count (x 1000)
Fire discovery date (month)

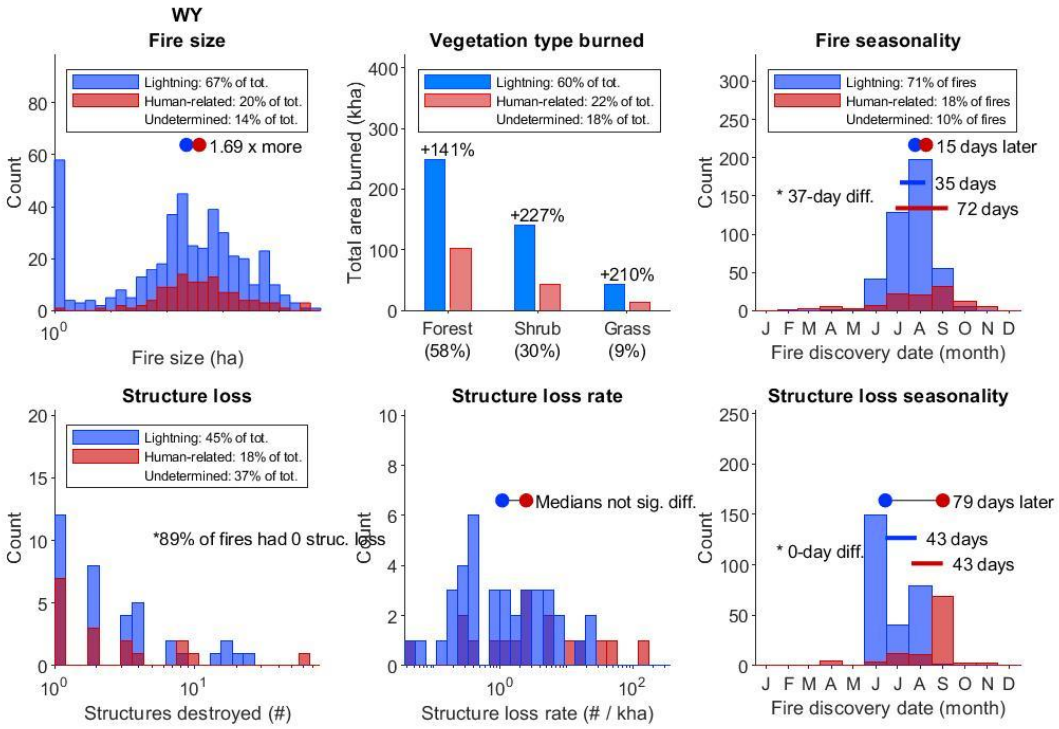
WY
Fire size
Lightning: 67% of tot.
Human-related: 20% of tot.
Undetermined: 14% of tot.
1.69 x more
Count
Fire size (ha)
Vegetation type burned
Lightning: 60% of tot.
Human-related: 22% of tot.
Undetermined: 18% of tot.
+141%
+227%
+210%
Total area burned (kha)
Forest (58%)
Shrub (30%)
Grass (9%)
Fire seasonality
Lightning: 71% of fires
Human-related: 18% of fires
Undetermined: 10% of fires
15 days later
35 days
72 days
* 37-day diff.
Fire discovery date (month)
J F M A M J J A S O N D
Structure loss
Lightning: 45% of tot.
Human-related: 18% of tot.
Undetermined: 37% of tot.
*89% of fires had 0 struc. loss
Structures destroyed (#)
Structure loss rate
Medians not sig. diff.
Structure loss rate (# / kha)
Structure loss seasonality
79 days later
43 days
43 days
* 0-day diff.
Fire discovery date (month)

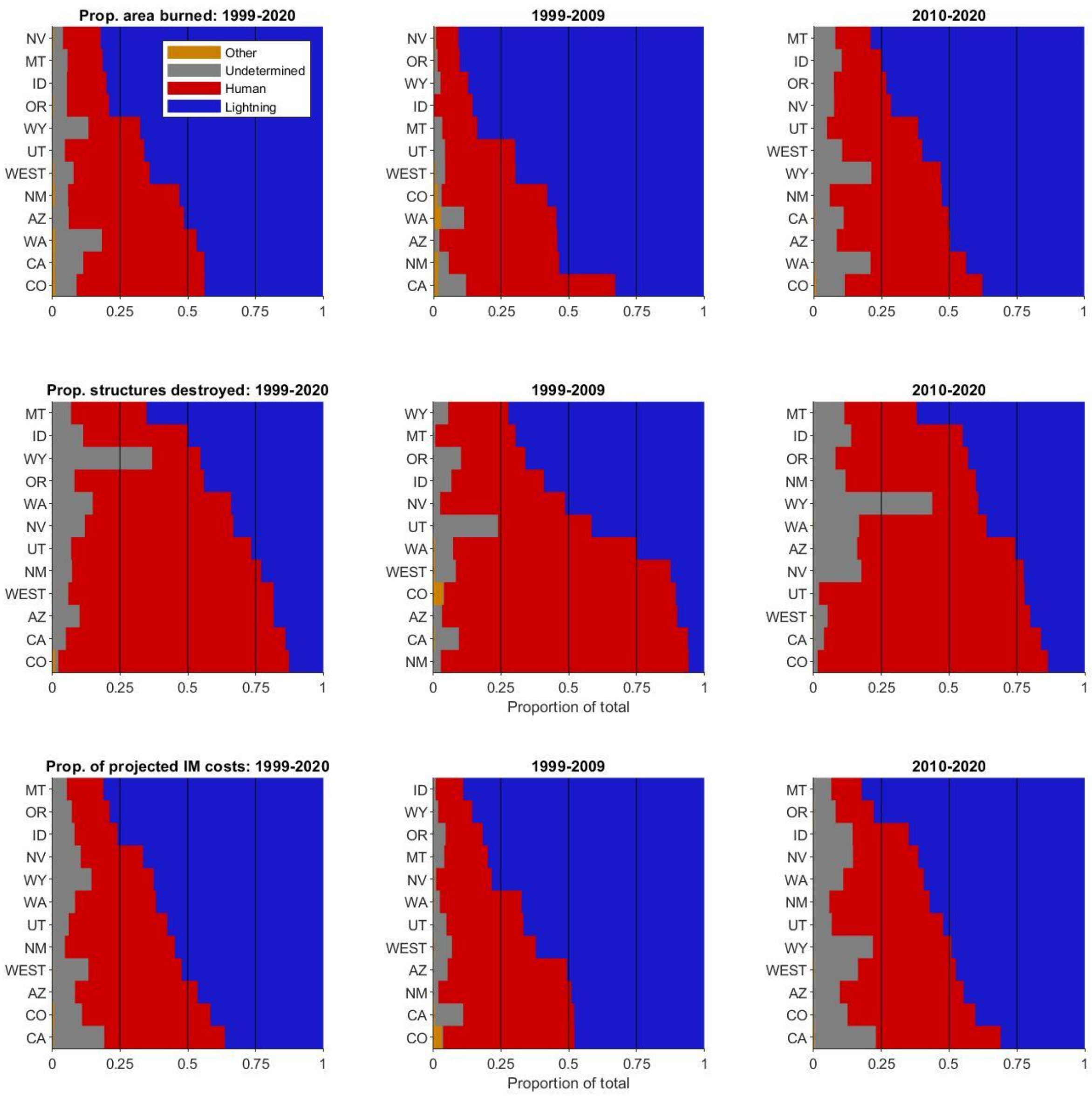

Fig. S6. State-level proportion of total area burned, structure loss, and incident-management costs by ignition classification in the raw dataset, by time period. Within each panel, states are ordered from highest to lowest based on the contribution from lightning-ignited fires, including the West-wide value ("WEST"). Projected incident-management (IM) costs do not reflect final costs of each fire event, but are rather the estimated costs at the closing of the incident management phase.

Figure S7. Ecoregion-scale (Level II) temporal patterns of area burned and structure loss from wildfires in the western United States. Black lines are only shown if the annual total of the variable displayed in bars is correlated with VPD (A) or area burned (B-C). Level II ecoregions are visible at https://www.epa.gov/eco-research/ecoregions-north-america. NOTE: only areas within the 11 Western states included in the main analysis are included in these summaries.

BELOW

COLD DESERTS

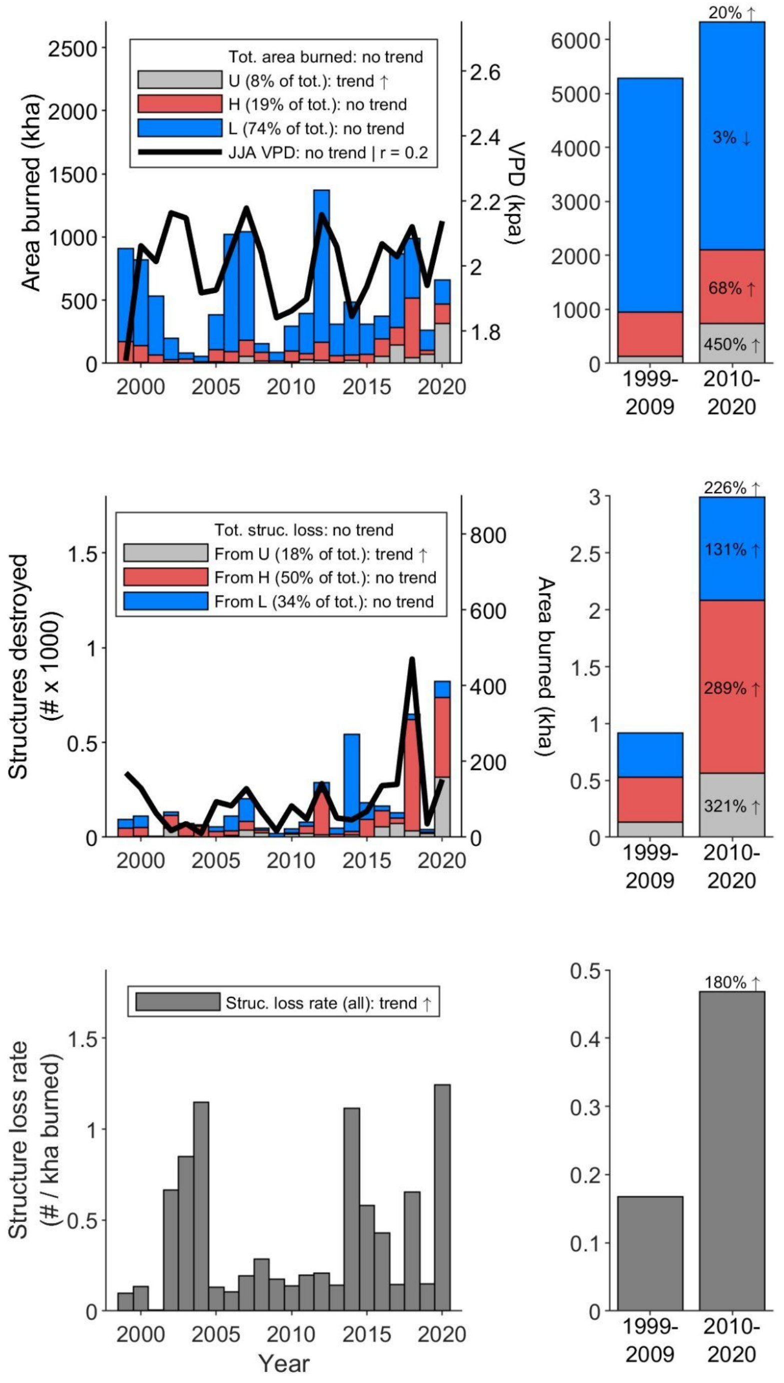

MARINE WEST COAST FOREST

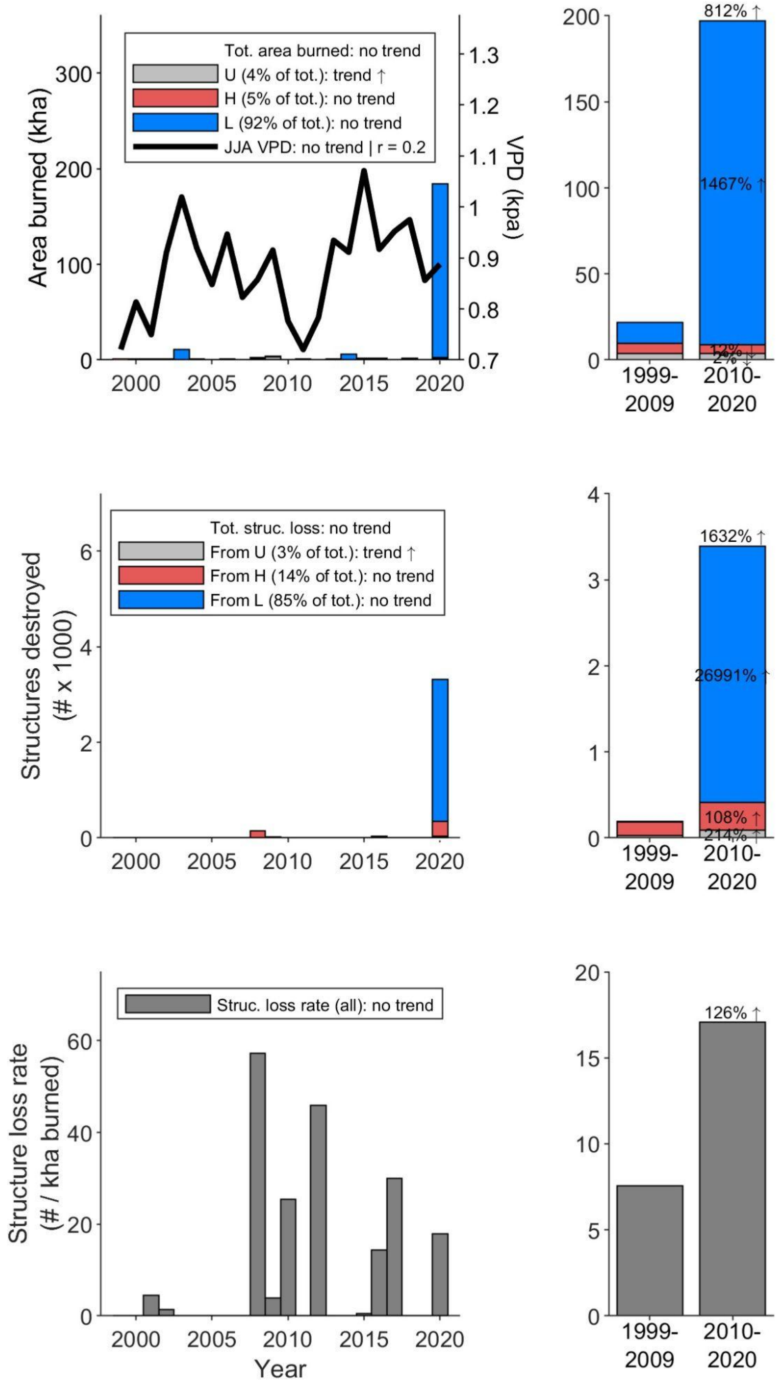

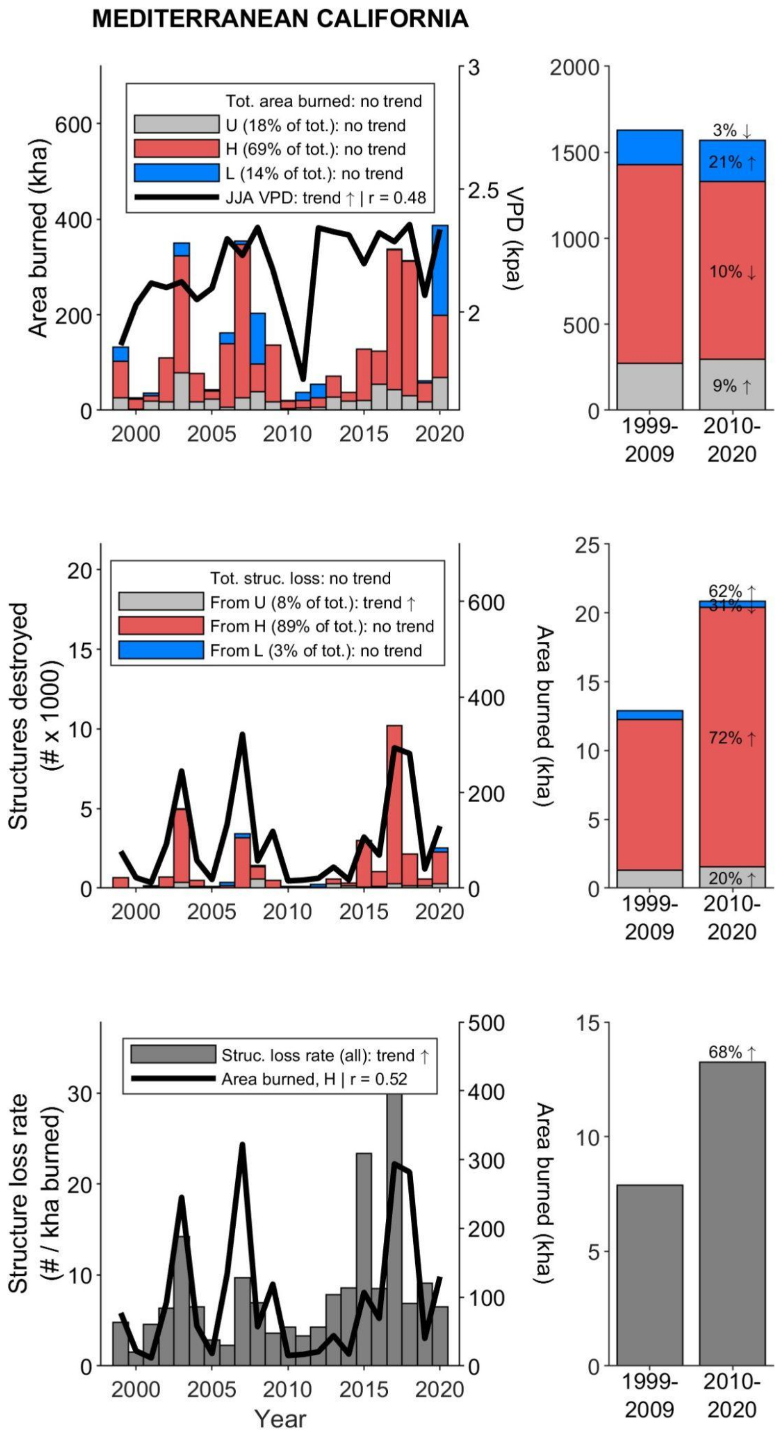

MEDITERRANEAN CALIFORNIA
Tot. area burned: no trend
U (18% of tot.): no trend
H (69% of tot.): no trend
L (14% of tot.): no trend
JJA VPD: trend ↑ | r = 0.48
Area burned (kha)
VPD (kpa)
3% ↓
21% ↑
10% ↓
9% ↑
1999-2009
2010-2020
Tot. struc. loss: no trend
From U (8% of tot.): trend ↑
From H (89% of tot.): no trend
From L (3% of tot.): no trend
Structures destroyed (# x 1000)
Area burned (kha)
62% ↑
31% ↓
72% ↑
20% ↑
Struc. loss rate (all): trend ↑
Area burned, H | r = 0.52
Structure loss rate (# / kha burned)
Area burned (kha)
68% ↑
Year

SOUTH CENTRAL SEMIARID PRAIRIES

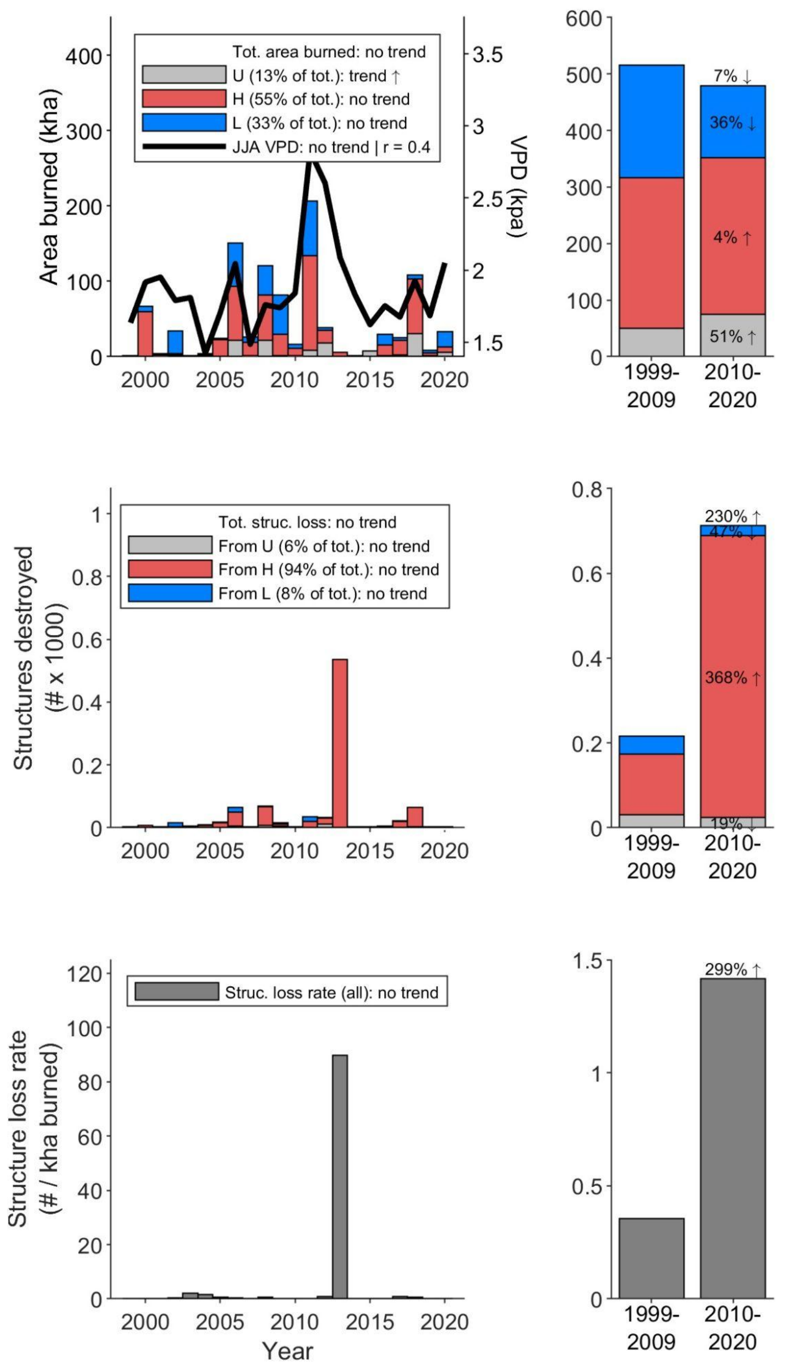

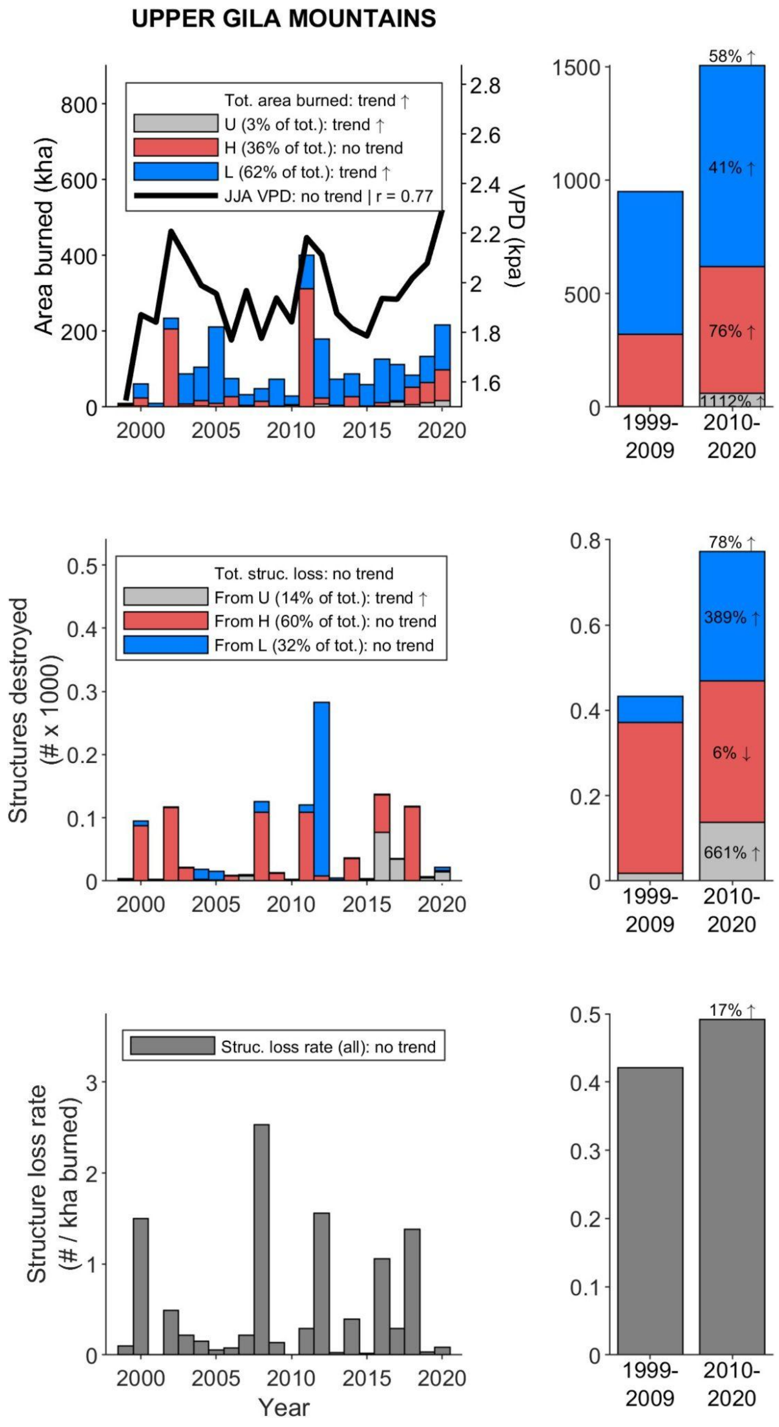
UPPER GILA MOUNTAINS
Tot. area burned: trend ↑
U (3% of tot.): trend ↑
H (36% of tot.): no trend
L (62% of tot.): trend ↑
JJA VPD: no trend | r = 0.77
Area burned (kha)
VPD (kpa)
58% ↑
41% ↑
76% ↑
1112% ↑
1999-2009
2010-2020
Tot. struc. loss: no trend
From U (14% of tot.): trend ↑
From H (60% of tot.): no trend
From L (32% of tot.): no trend
Structures destroyed (# x 1000)
78% ↑
389% ↑
6% ↓
661% ↑
Struc. loss rate (all): no trend
Structure loss rate (# / kha burned)
17% ↑
Year

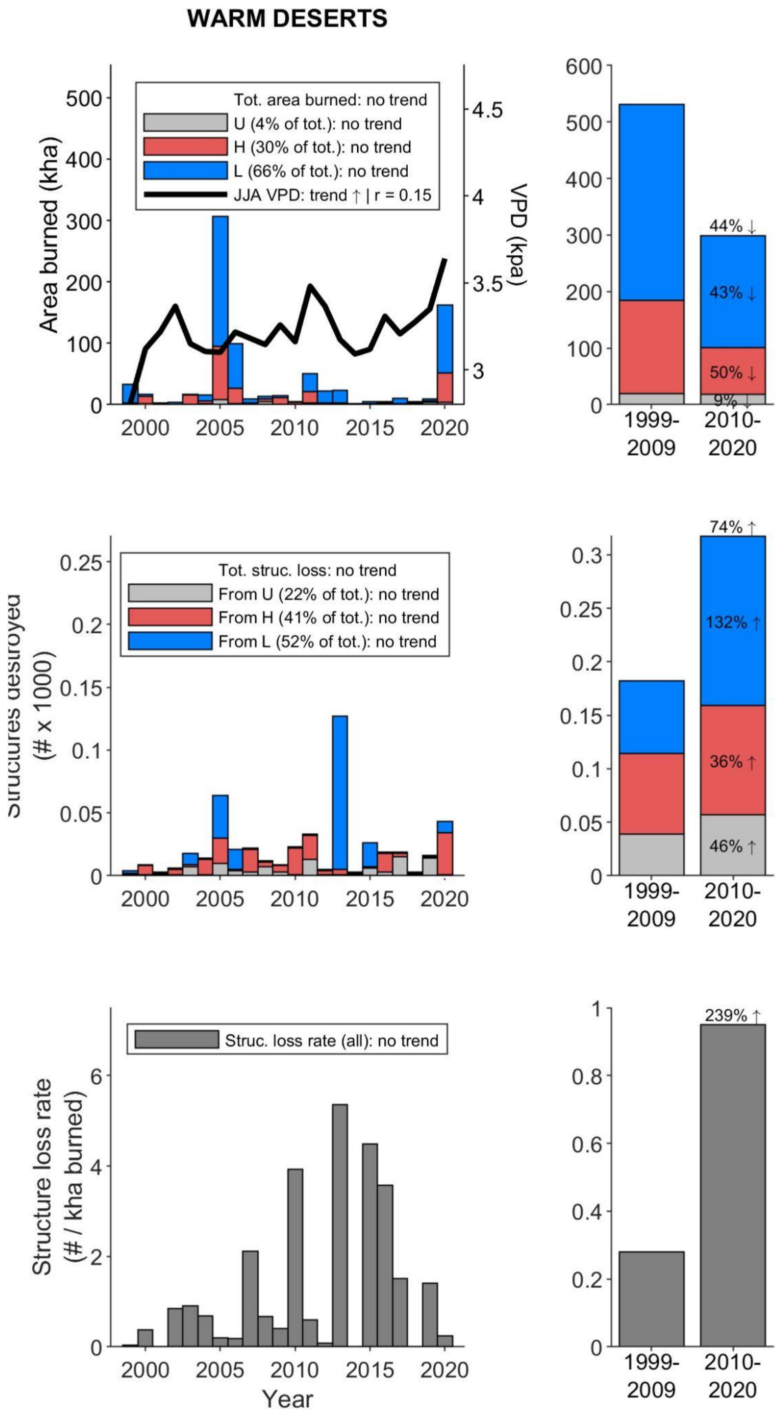
WARM DESERTS
Tot. area burned: no trend
U (4% of tot.): no trend
H (30% of tot.): no trend
L (66% of tot.): no trend
JJA VPD: trend ↑ | r = 0.15
Area burned (kha)
VPD (kpa)
44% ↓
43% ↓
50% ↓
9% ↓
1999-2009
2010-2020
Tot. struc. loss: no trend
From U (22% of tot.): no trend
From H (41% of tot.): no trend
From L (52% of tot.): no trend
Structures destroyed (# x 1000)
74% ↑
132% ↑
36% ↑
46% ↑
Struc. loss rate (all): no trend
Structure loss rate (# / kha burned)
239% ↑
Year

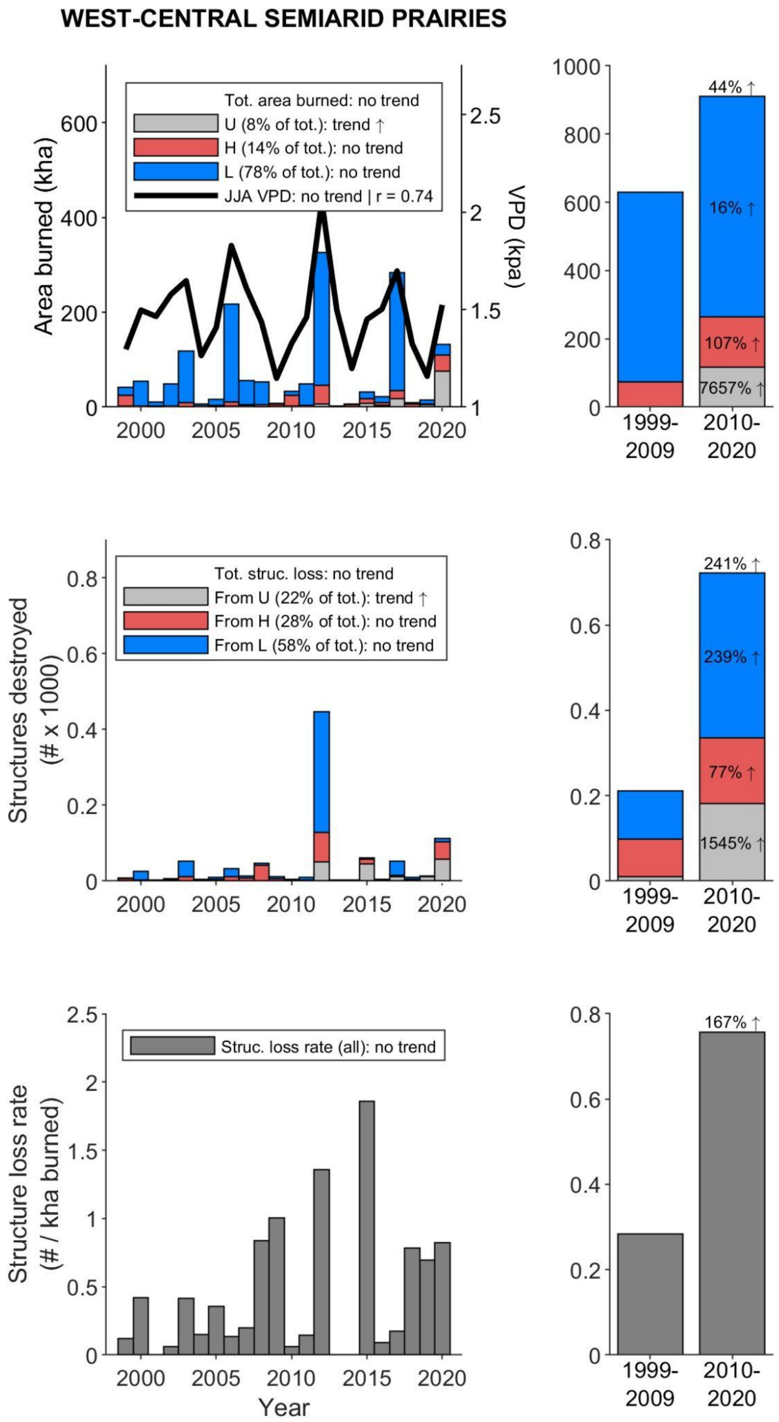
WEST-CENTRAL SEMIARID PRAIRIES
Tot. area burned: no trend
U (8% of tot.): trend ↑
H (14% of tot.): no trend
L (78% of tot.): no trend
JJA VPD: no trend | r = 0.74
Area burned (kha)
VPD (kpa)
44% ↑
16% ↑
107% ↑
7657% ↑
1999-2009
2010-2020
Tot. struc. loss: no trend
From U (22% of tot.): trend ↑
From H (28% of tot.): no trend
From L (58% of tot.): no trend
Structures destroyed (# x 1000)
241% ↑
239% ↑
77% ↑
1545% ↑
Struc. loss rate (all): no trend
Structure loss rate (# / kha burned)
Year
167% ↑

WESTERN CORDILLERA

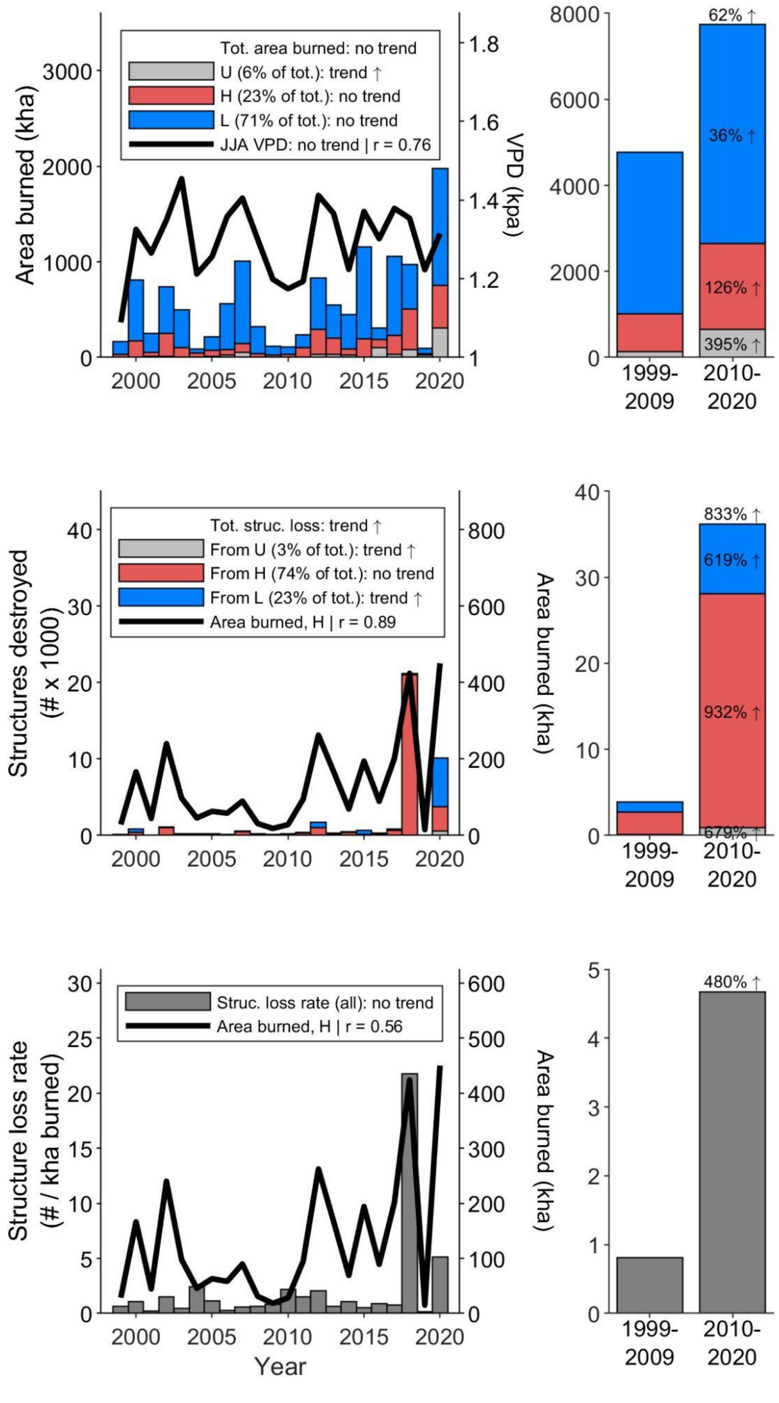

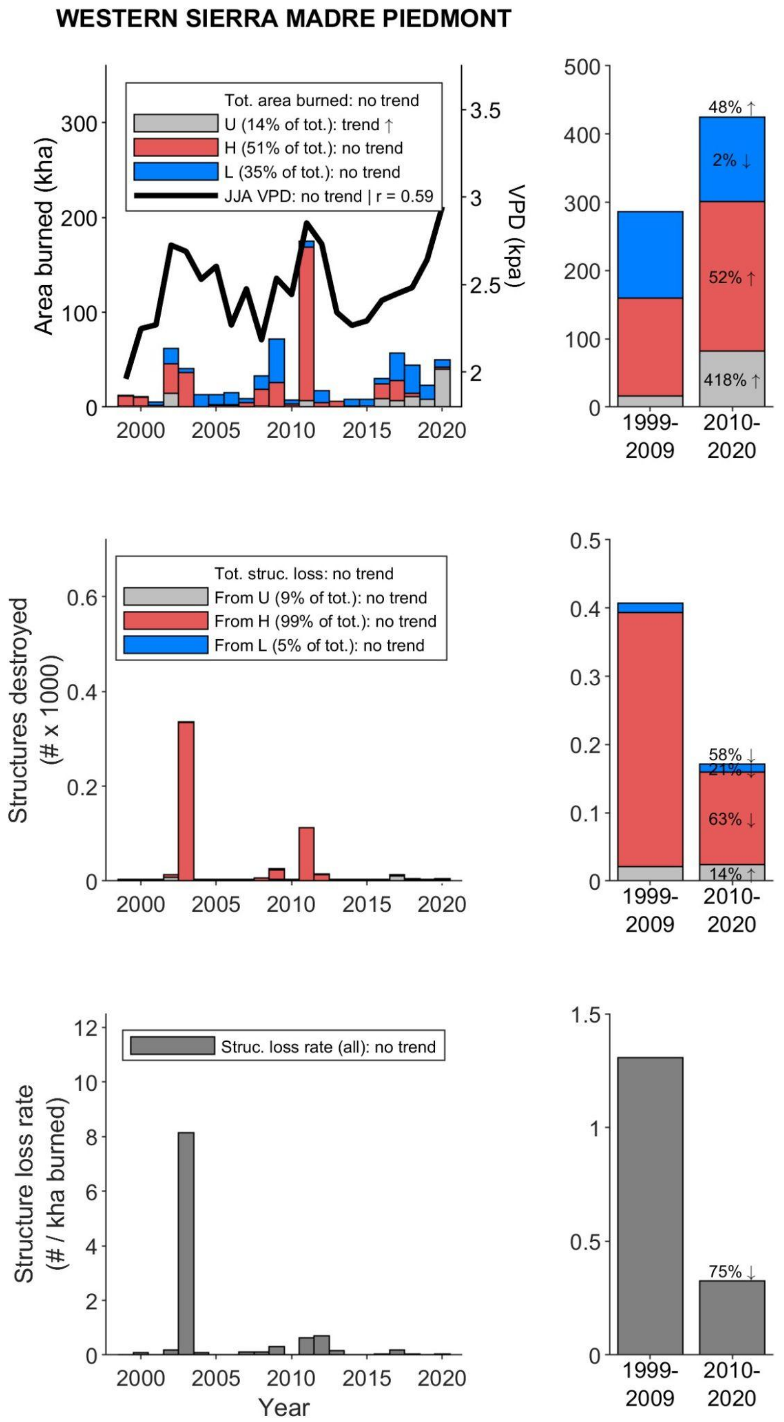
WESTERN SIERRA MADRE PIEDMONT
Tot. area burned: no trend
U (14% of tot.): trend ↑
H (51% of tot.): no trend
L (35% of tot.): no trend
JJA VPD: no trend | r = 0.59
Area burned (kha)
VPD (kpa)
48% ↑
2% ↓
52% ↑
418% ↑
1999-2009
2010-2020
Tot. struc. loss: no trend
From U (9% of tot.): no trend
From H (99% of tot.): no trend
From L (5% of tot.): no trend
Structures destroyed (# x 1000)
58% ↓
21% ↓
63% ↓
14% ↑
Struc. loss rate (all): no trend
Structure loss rate (# / kha burned)
75% ↓
Year

Figure S8. Ecoregion-level fire regime attributes by ignition source for the western United States. As in Figure 3 in the main text; legend is the same for the entire bottom row. Non-significant between-median values are labeled with "Medians not sig. diff." if the Wilcoxon rank-sum tests yielded a p-value > 0.10. Level II ecoregions are visible at https://www.epa.gov/eco-research/ecoregions-north-america. NOTE: only areas within the 11 Western states included in the main analysis are included in these summaries.

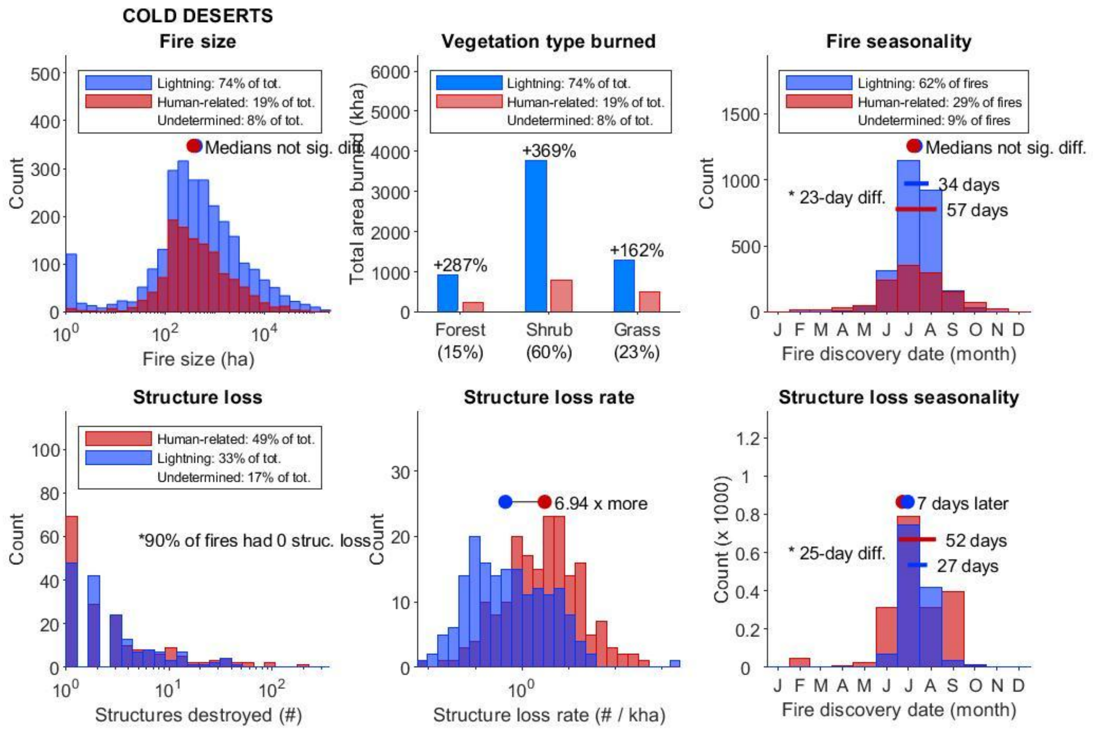

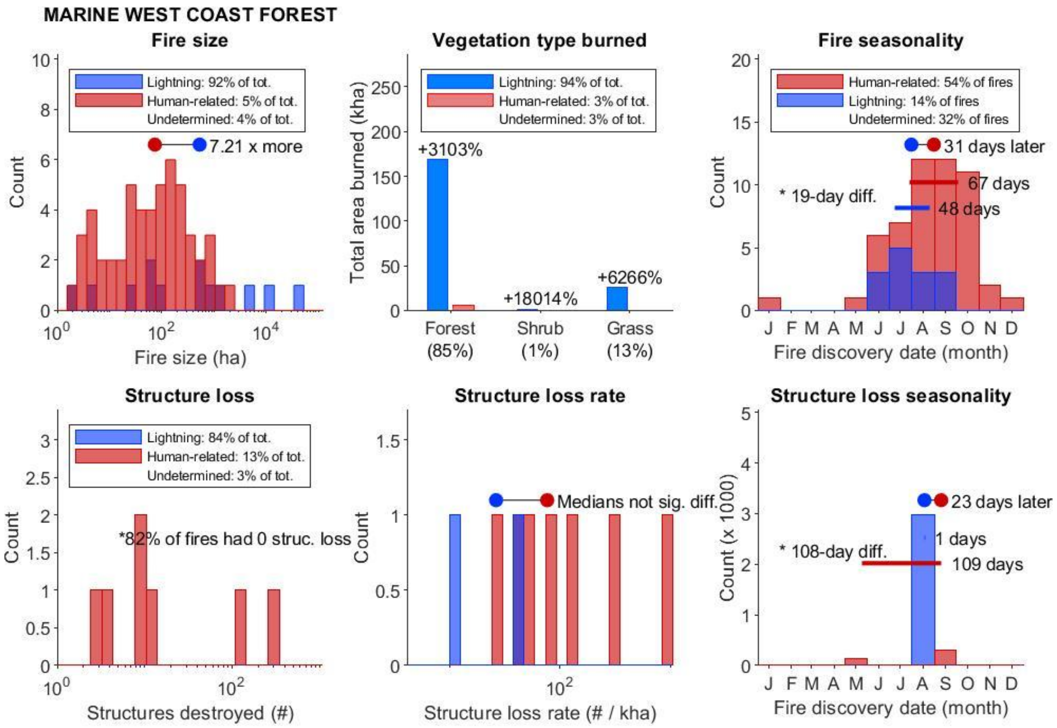
MARINE WEST COAST FOREST
Fire size
Lightning: 92% of tot.
Human-related: 5% of tot.
Undetermined: 4% of tot.
7.21 x more
Count
Fire size (ha)
Vegetation type burned
Lightning: 94% of tot.
Human-related: 3% of tot.
Undetermined: 3% of tot.
+3103%
+18014%
+6266%
Total area burned (kha)
Forest (85%)
Shrub (1%)
Grass (13%)
Fire seasonality
Human-related: 54% of fires
Lightning: 14% of fires
Undetermined: 32% of fires
31 days later
67 days
48 days
* 19-day diff.
Fire discovery date (month)
Structure loss
Lightning: 84% of tot.
Human-related: 13% of tot.
Undetermined: 3% of tot.
*82% of fires had 0 struc. loss
Structures destroyed (#)
Structure loss rate
Medians not sig. diff.
Structure loss rate (# / kha)
Structure loss seasonality
23 days later
1 days
109 days
* 108-day diff.
Count (x 1000)
Fire discovery date (month)

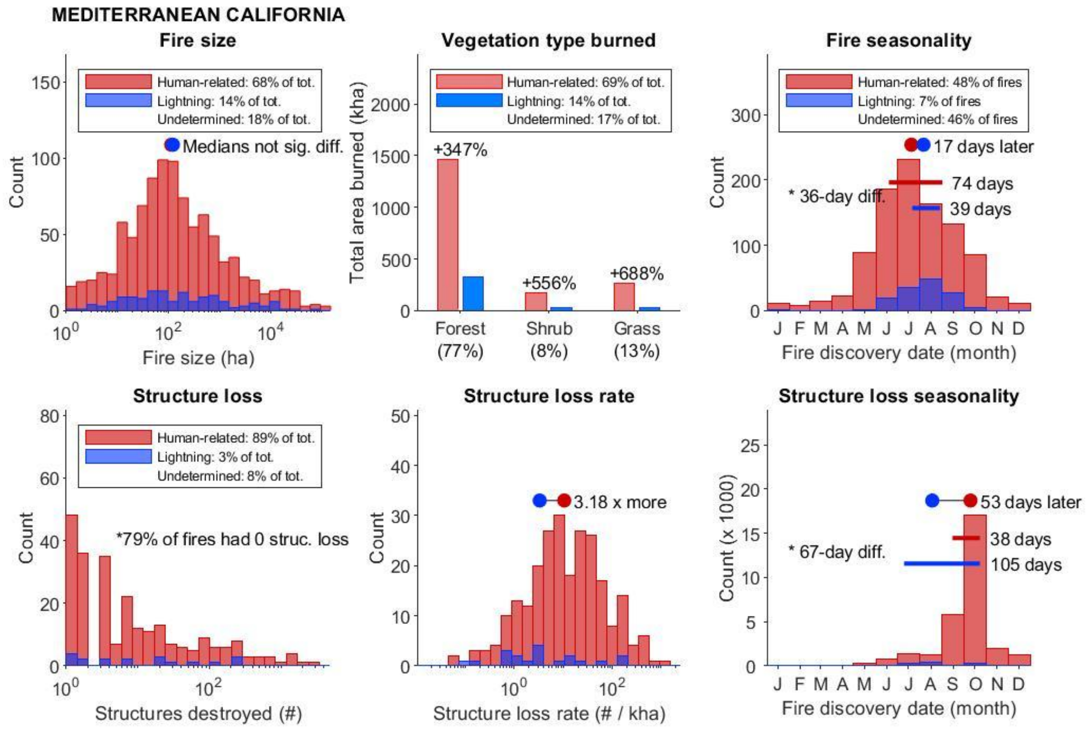
MEDITERRANEAN CALIFORNIA
Fire size
Human-related: 68% of tot.
Lightning: 14% of tot.
Undetermined: 18% of tot.
Medians not sig. diff.
Count
Fire size (ha)
Vegetation type burned
Human-related: 69% of tot.
Lightning: 14% of tot.
Undetermined: 17% of tot.
+347%
+556%
+688%
Total area burned (kha)
Forest (77%)
Shrub (8%)
Grass (13%)
Fire seasonality
Human-related: 48% of fires
Lightning: 7% of fires
Undetermined: 46% of fires
17 days later
74 days
39 days
* 36-day diff.
Fire discovery date (month)
Structure loss
Human-related: 89% of tot.
Lightning: 3% of tot.
Undetermined: 8% of tot.
*79% of fires had 0 struc. loss
Structures destroyed (#)
Structure loss rate
3.18 x more
Structure loss rate (# / kha)
Structure loss seasonality
53 days later
38 days
105 days
* 67-day diff.
Count (x 1000)
Fire discovery date (month)

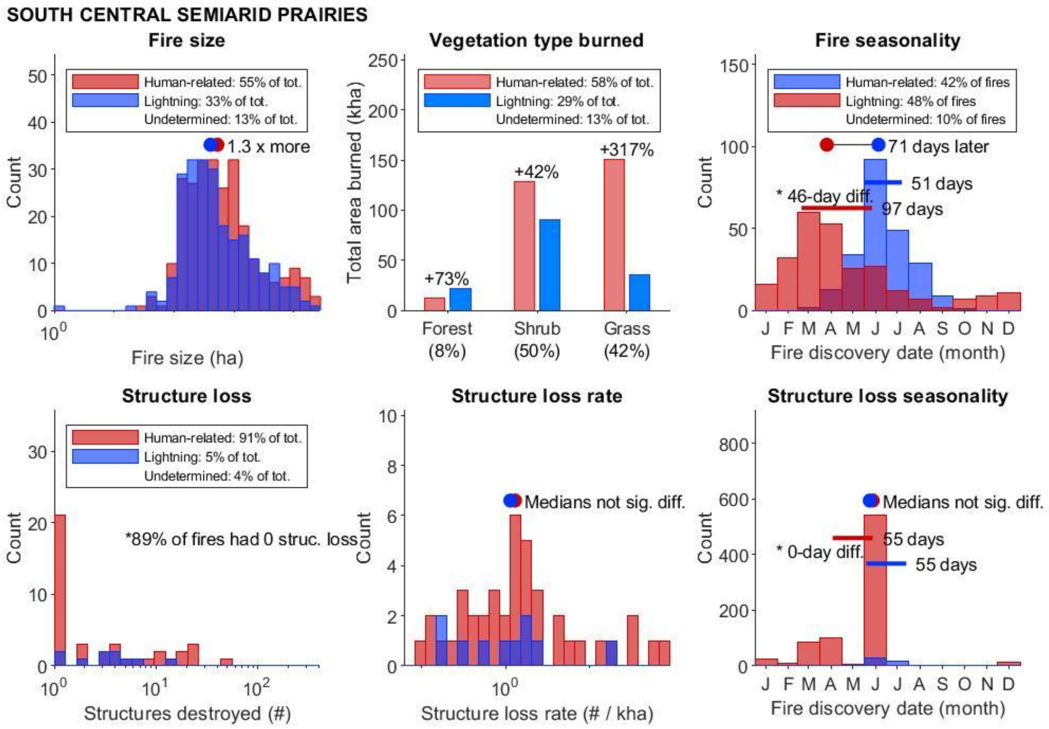
SOUTH CENTRAL SEMIARID PRAIRIES
Fire size
Human-related: 55% of tot.
Lightning: 33% of tot.
Undetermined: 13% of tot.
1.3 x more
Count
Fire size (ha)
Vegetation type burned
Human-related: 58% of tot.
Lightning: 29% of tot.
Undetermined: 13% of tot.
+73%
+42%
+317%
Total area burned (kha)
Forest (8%)
Shrub (50%)
Grass (42%)
Fire seasonality
Human-related: 42% of fires
Lightning: 48% of fires
Undetermined: 10% of fires
71 days later
51 days
* 46-day diff.
97 days
Fire discovery date (month)
Structure loss
Human-related: 91% of tot.
Lightning: 5% of tot.
Undetermined: 4% of tot.
*89% of fires had 0 struc. loss
Structures destroyed (#)
Structure loss rate
Medians not sig. diff.
Structure loss rate (# / kha)
Structure loss seasonality
Medians not sig. diff.
55 days
* 0-day diff.
55 days
Fire discovery date (month)

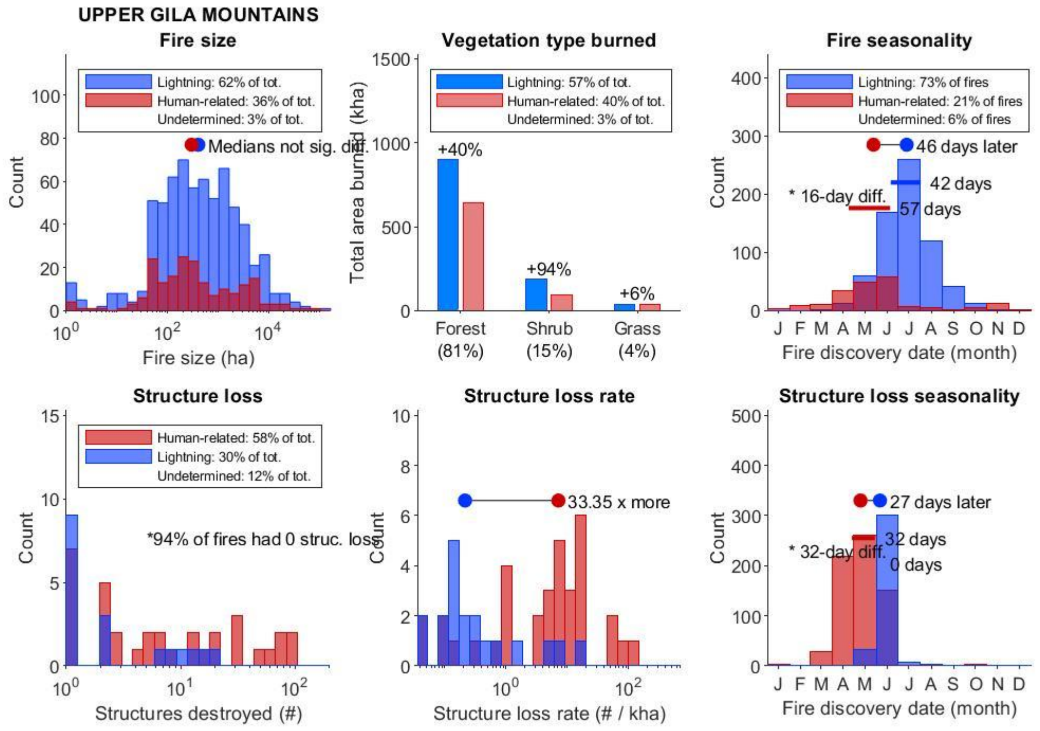
UPPER GILA MOUNTAINS
Fire size
Lightning: 62% of tot.
Human-related: 36% of tot.
Undetermined: 3% of tot.
Medians not sig. diff.
Count
Fire size (ha)
Vegetation type burned
Lightning: 57% of tot.
Human-related: 40% of tot.
Undetermined: 3% of tot.
Total area burned (kha)
+40%
+94%
+6%
Forest (81%)
Shrub (15%)
Grass (4%)
Fire seasonality
Lightning: 73% of fires
Human-related: 21% of fires
Undetermined: 6% of fires
46 days later
42 days
* 16-day diff.
57 days
Fire discovery date (month)
Structure loss
Human-related: 58% of tot.
Lightning: 30% of tot.
Undetermined: 12% of tot.
*94% of fires had 0 struc. loss
Structures destroyed (#)
Structure loss rate
33.35 x more
Structure loss rate (# / kha)
Structure loss seasonality
27 days later
* 32-day diff.
32 days
0 days
Fire discovery date (month)

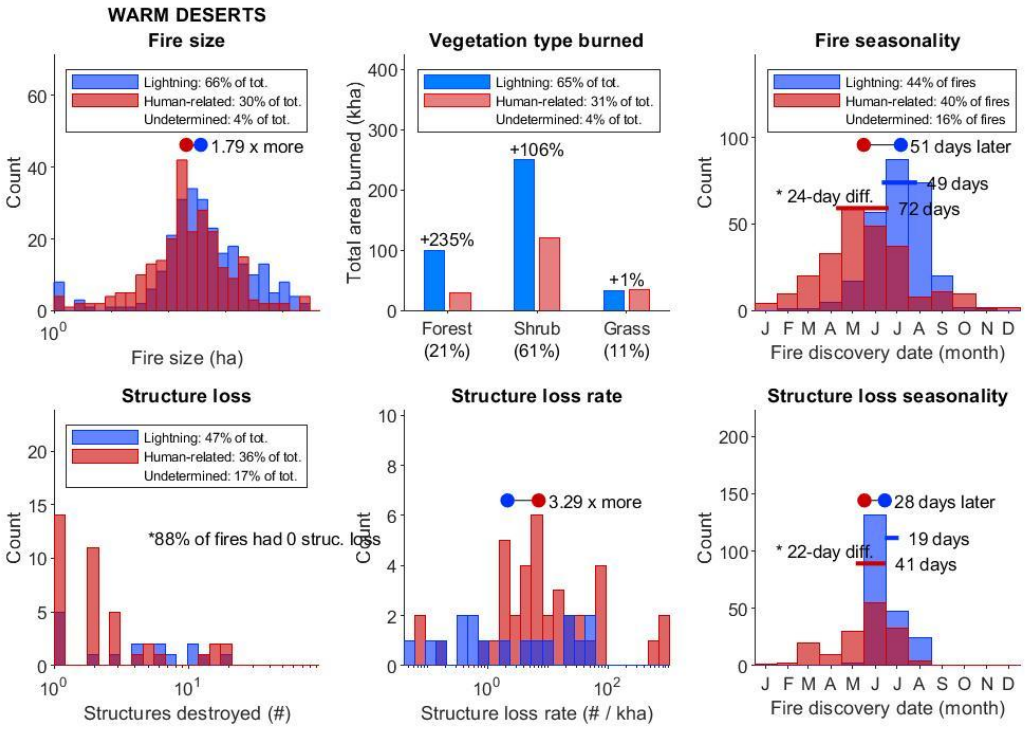
WARM DESERTS
Fire size
Lightning: 66% of tot.
Human-related: 30% of tot.
Undetermined: 4% of tot.
1.79 x more
Count
Fire size (ha)
Vegetation type burned
Lightning: 65% of tot.
Human-related: 31% of tot.
Undetermined: 4% of tot.
Total area burned (kha)
+235%
+106%
+1%
Forest (21%)
Shrub (61%)
Grass (11%)
Fire seasonality
Lightning: 44% of fires
Human-related: 40% of fires
Undetermined: 16% of fires
51 days later
49 days
* 24-day diff.
72 days
Fire discovery date (month)
Structure loss
Lightning: 47% of tot.
Human-related: 36% of tot.
Undetermined: 17% of tot.
*88% of fires had 0 struc. loss
Structures destroyed (#)
Structure loss rate
3.29 x more
Structure loss rate (# / kha)
Structure loss seasonality
28 days later
19 days
* 22-day diff.
41 days
Fire discovery date (month)

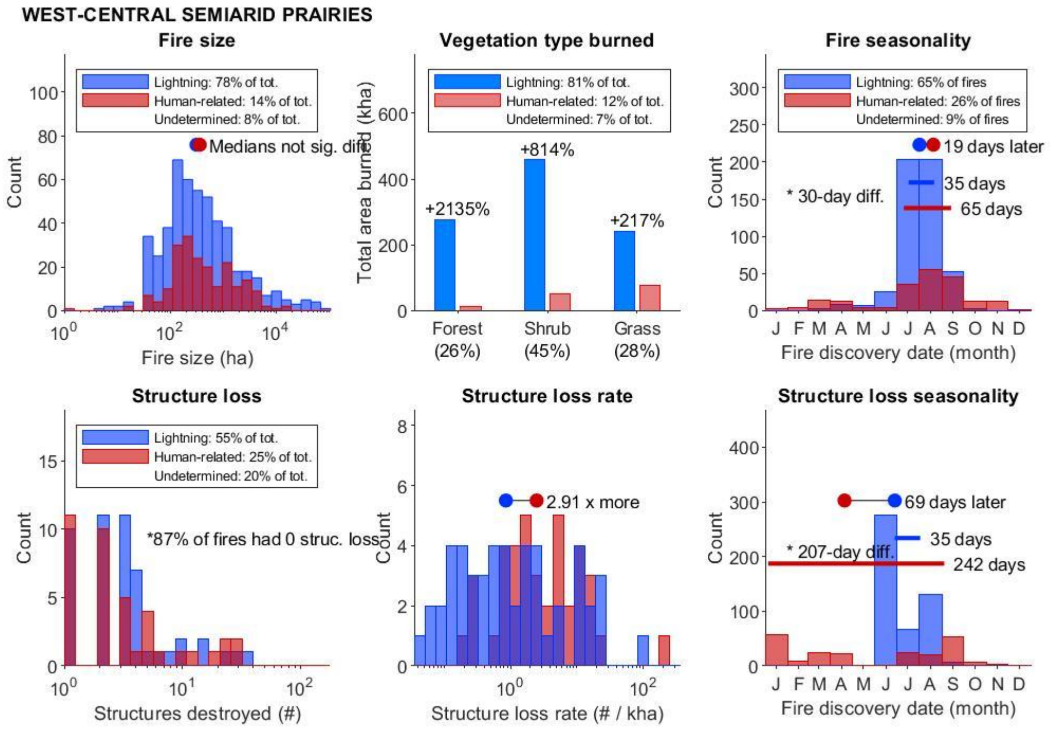
WEST-CENTRAL SEMIARID PRAIRIES
Fire size
Lightning: 78% of tot.
Human-related: 14% of tot.
Undetermined: 8% of tot.
Medians not sig. diff.
Count
Fire size (ha)
Vegetation type burned
Lightning: 81% of tot.
Human-related: 12% of tot.
Undetermined: 7% of tot.
Total area burned (kha)
+2135%
+814%
+217%
Forest (26%)
Shrub (45%)
Grass (28%)
Fire seasonality
Lightning: 65% of fires
Human-related: 26% of fires
Undetermined: 9% of fires
19 days later
35 days
65 days
* 30-day diff.
Fire discovery date (month)
J F M A M J J A S O N D
Structure loss
Lightning: 55% of tot.
Human-related: 25% of tot.
Undetermined: 20% of tot.
*87% of fires had 0 struc. loss
Structures destroyed (#)
Structure loss rate
2.91 x more
Structure loss rate (# / kha)
Structure loss seasonality
69 days later
35 days
* 207-day diff.
242 days
Fire discovery date (month)

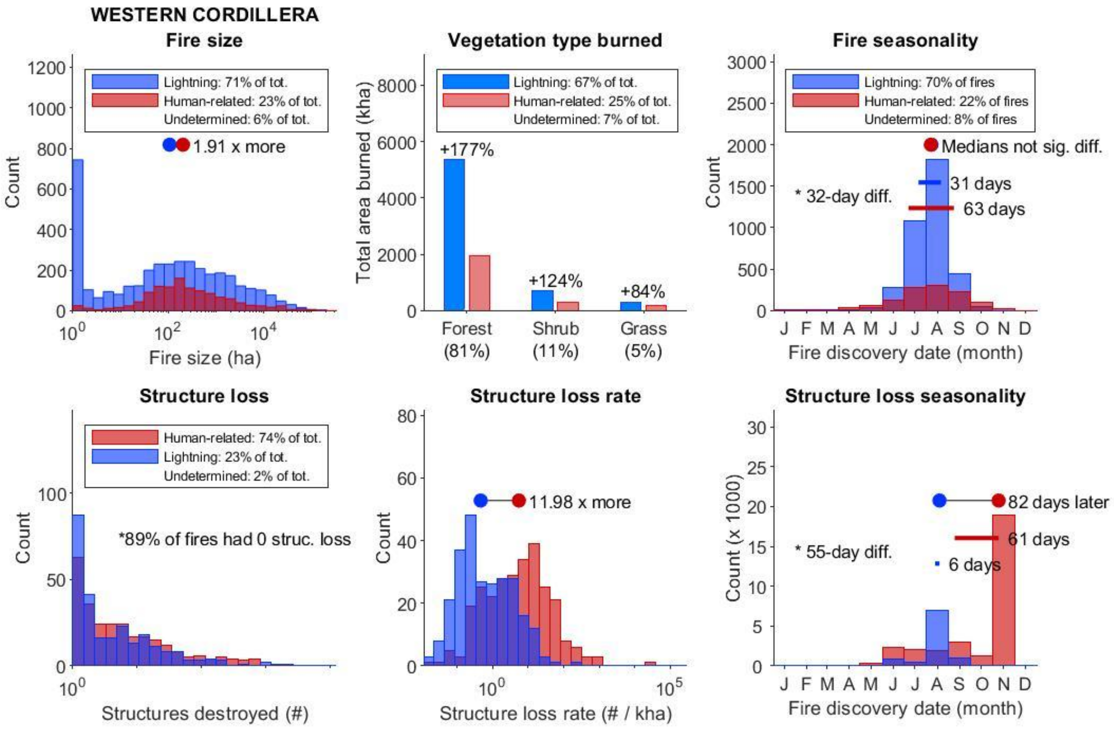
WESTERN CORDILLERA
Fire size
Lightning: 71% of tot.
Human-related: 23% of tot.
Undetermined: 6% of tot.
1.91 x more
Count
Fire size (ha)
Vegetation type burned
Lightning: 67% of tot.
Human-related: 25% of tot.
Undetermined: 7% of tot.
+177%
+124%
+84%
Total area burned (kha)
Forest (81%)
Shrub (11%)
Grass (5%)
Fire seasonality
Lightning: 70% of fires
Human-related: 22% of fires
Undetermined: 8% of fires
Medians not sig. diff.
* 32-day diff.
31 days
63 days
Count
J F M A M J J A S O N D
Fire discovery date (month)
Structure loss
Human-related: 74% of tot.
Lightning: 23% of tot.
Undetermined: 2% of tot.
*89% of fires had 0 struc. loss
Count
Structures destroyed (#)
Structure loss rate
11.98 x more
Count
Structure loss rate (# / kha)
Structure loss seasonality
82 days later
* 55-day diff.
61 days
6 days
Count (x 1000)
J F M A M J J A S O N D
Fire discovery date (month)

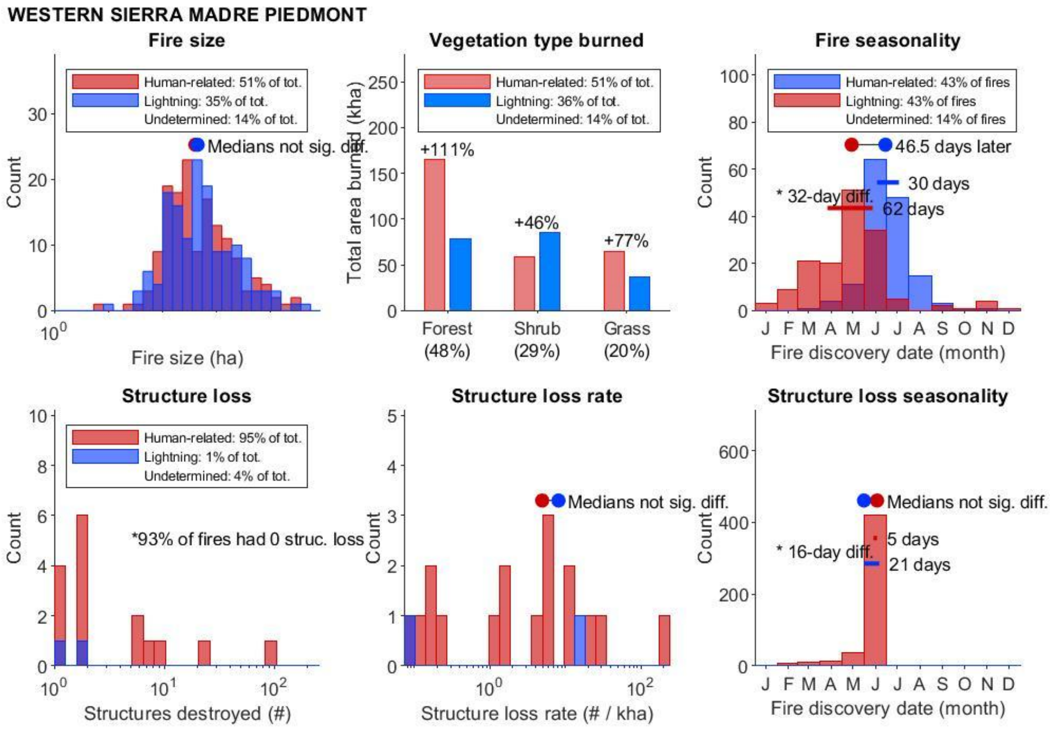
WESTERN SIERRA MADRE PIEDMONT
Fire size
Human-related: 51% of tot.
Lightning: 35% of tot.
Undetermined: 14% of tot.
Medians not sig. diff.
Count
Fire size (ha)
Vegetation type burned
Human-related: 51% of tot.
Lightning: 36% of tot.
Undetermined: 14% of tot.
Total area burned (kha)
+111%
+46%
+77%
Forest (48%)
Shrub (29%)
Grass (20%)
Fire seasonality
Human-related: 43% of fires
Lightning: 43% of fires
Undetermined: 14% of fires
46.5 days later
30 days
* 32-day diff.
62 days
Fire discovery date (month)
Structure loss
Human-related: 95% of tot.
Lightning: 1% of tot.
Undetermined: 4% of tot.
*93% of fires had 0 struc. loss
Structures destroyed (#)
Structure loss rate
Medians not sig. diff.
Structure loss rate (# / kha)
Structure loss seasonality
Medians not sig. diff.
5 days
* 16-day diff.
21 days
Fire discovery date (month)

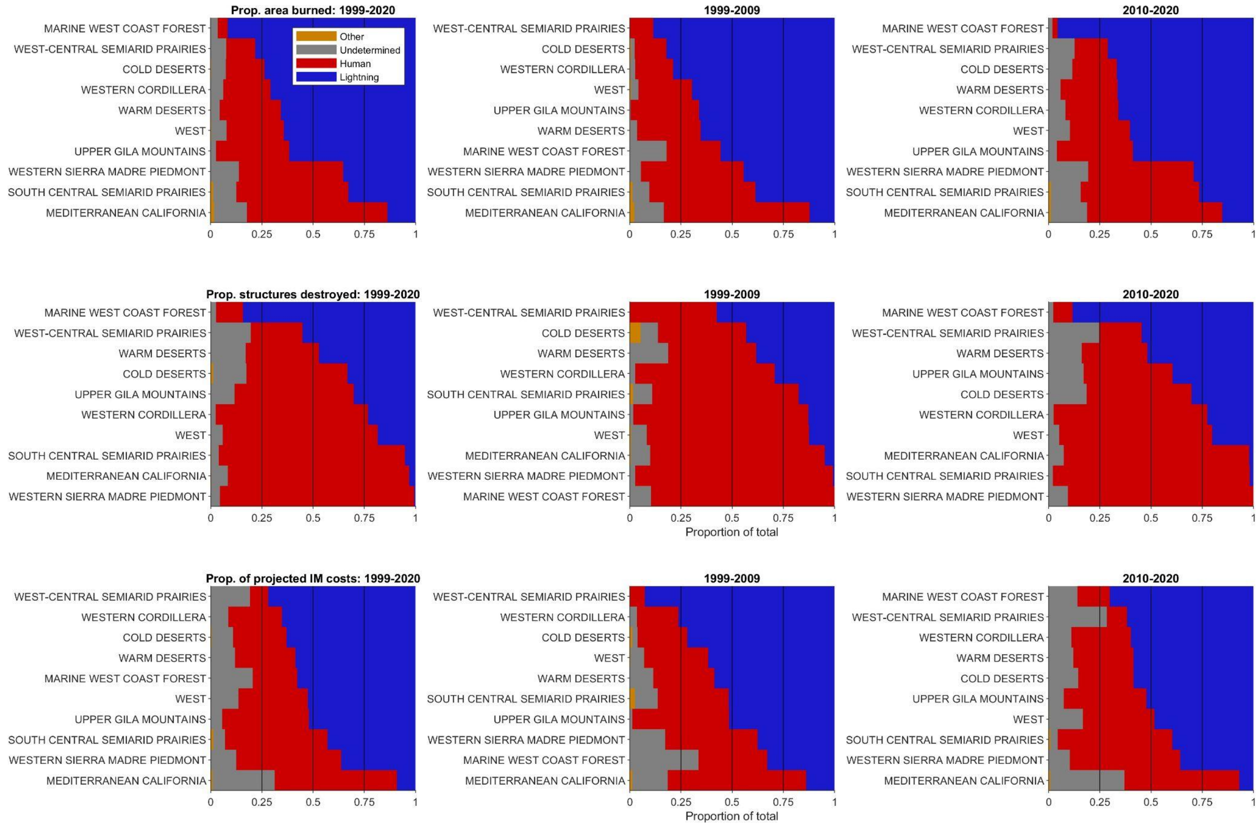

Fig. S9. Ecoregion-level proportion of total area burned, structure loss, and incident-management costs by ignition classification in the raw dataset, by time period. Within each panel, states are ordered from highest to lowest based on the contribution from lightning-ignited fires, including the West-wide value ("WEST"). Projected incident-management (IM) costs do not reflect final costs of each fire event, but are rather the estimated costs at the closing of the incident management phase.

Figure S10. GACC-scale temporal patterns of area burned and structure loss from wildfires in the western United States. Black lines are only shown if the annual total of the variable displayed in bars is correlated with VPD (A) or area burned (B-C). Geographic area coordination center (GACC) delineations are visible at https://gacc.nifc.gov/. NOTE: Areas outside of Montana, Wyoming, and Colorado are NOT included in the Northern Rockies and Rocky Mountain GACC summaries.

BELOW

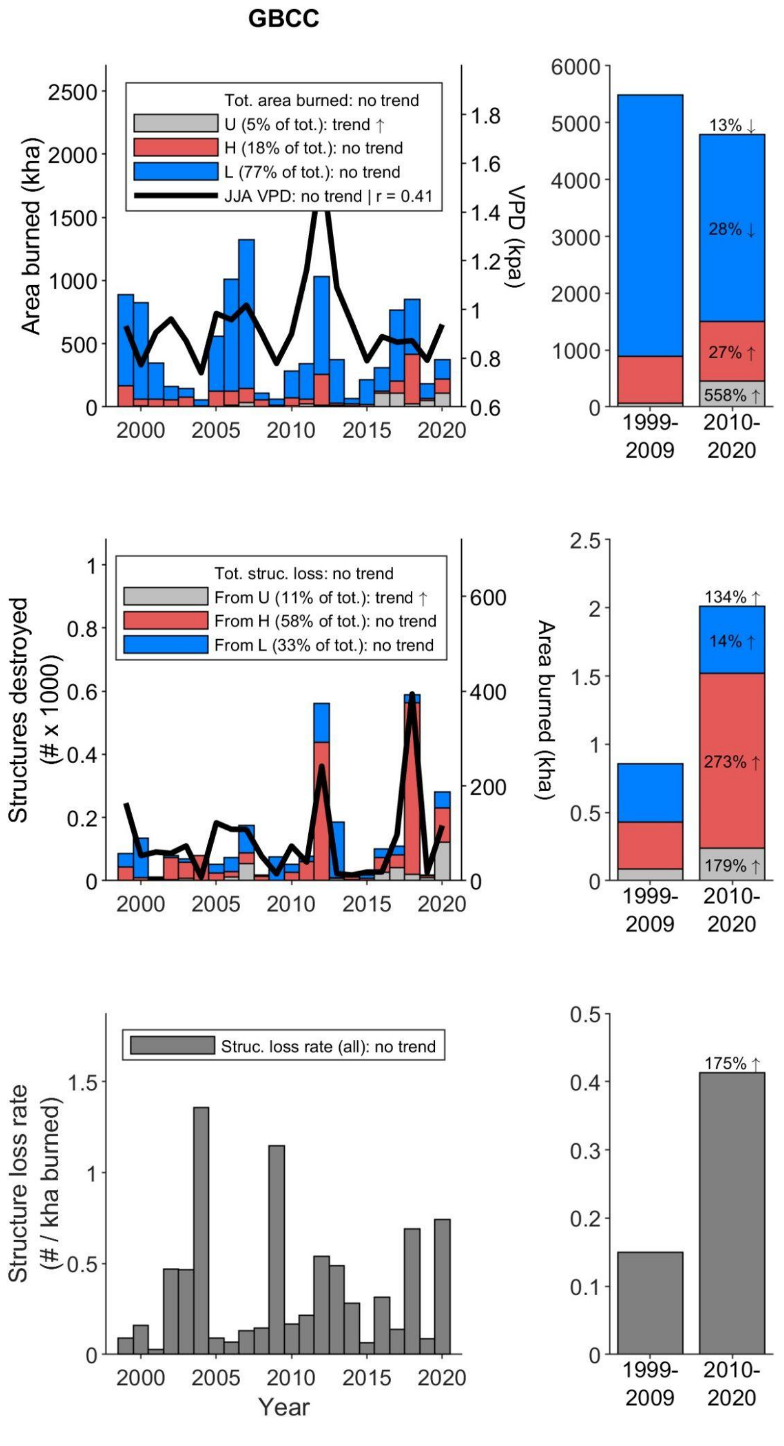
GBCC
Area burned (kha)
Tot. area burned: no trend
U (5% of tot.): trend ↑
H (18% of tot.): no trend
L (77% of tot.): no trend
JJA VPD: no trend | r = 0.41
VPD (kpa)
13% ↓
28% ↓
27% ↑
558% ↑
1999-2009
2010-2020
Structures destroyed (# x 1000)
Tot. struc. loss: no trend
From U (11% of tot.): trend ↑
From H (58% of tot.): no trend
From L (33% of tot.): no trend
Area burned (kha)
134% ↑
14% ↑
273% ↑
179% ↑
Structure loss rate (# / kha burned)
Struc. loss rate (all): no trend
175% ↑
Year

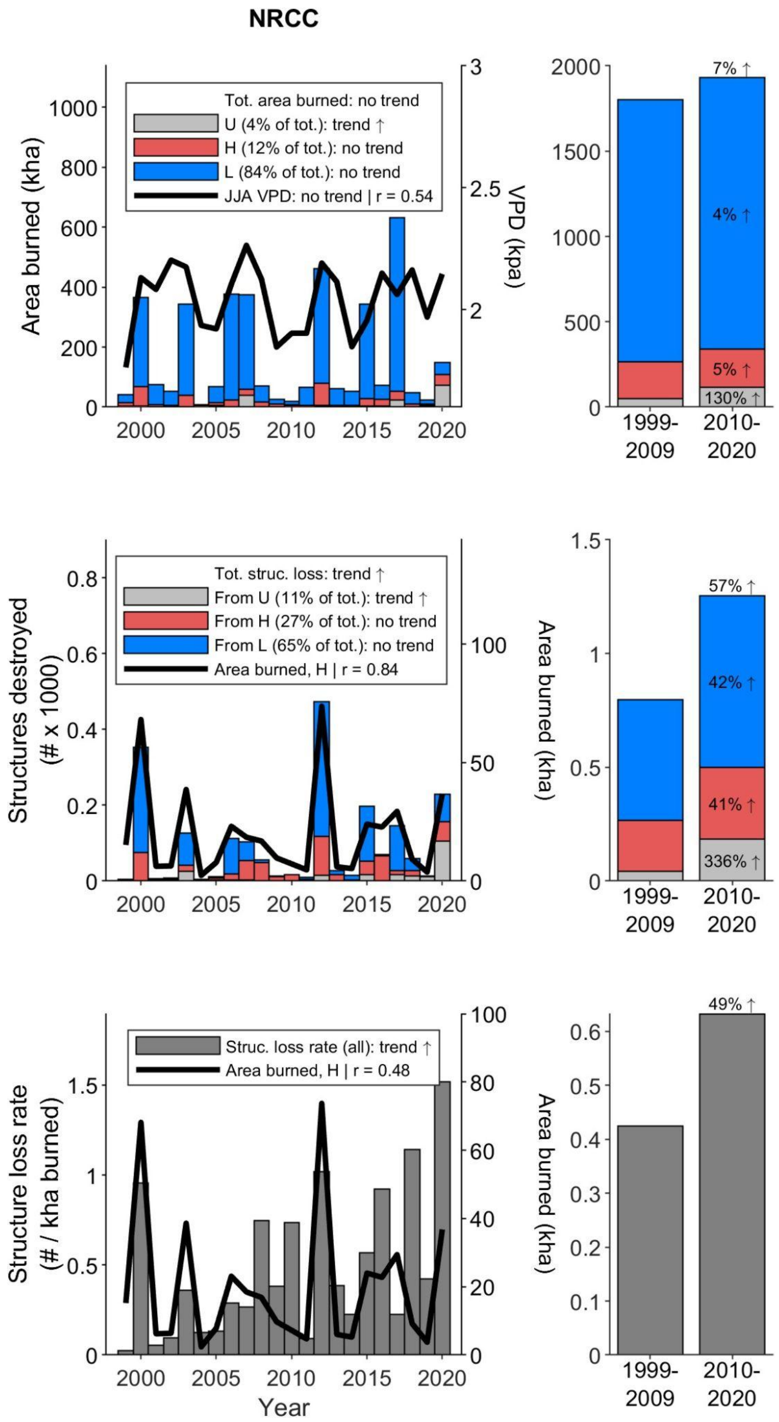
NRCC
Tot. area burned: no trend
U (4% of tot.): trend ↑
H (12% of tot.): no trend
L (84% of tot.): no trend
JJA VPD: no trend | r = 0.54
Area burned (kha)
VPD (kpa)
7% ↑
4% ↑
5% ↑
130% ↑
1999-2009
2010-2020
Tot. struc. loss: trend ↑
From U (11% of tot.): trend ↑
From H (27% of tot.): no trend
From L (65% of tot.): no trend
Area burned, H | r = 0.84
Structures destroyed (# x 1000)
Area burned (kha)
57% ↑
42% ↑
41% ↑
336% ↑
Struc. loss rate (all): trend ↑
Area burned, H | r = 0.48
Structure loss rate (# / kha burned)
Area burned (kha)
Year
49% ↑

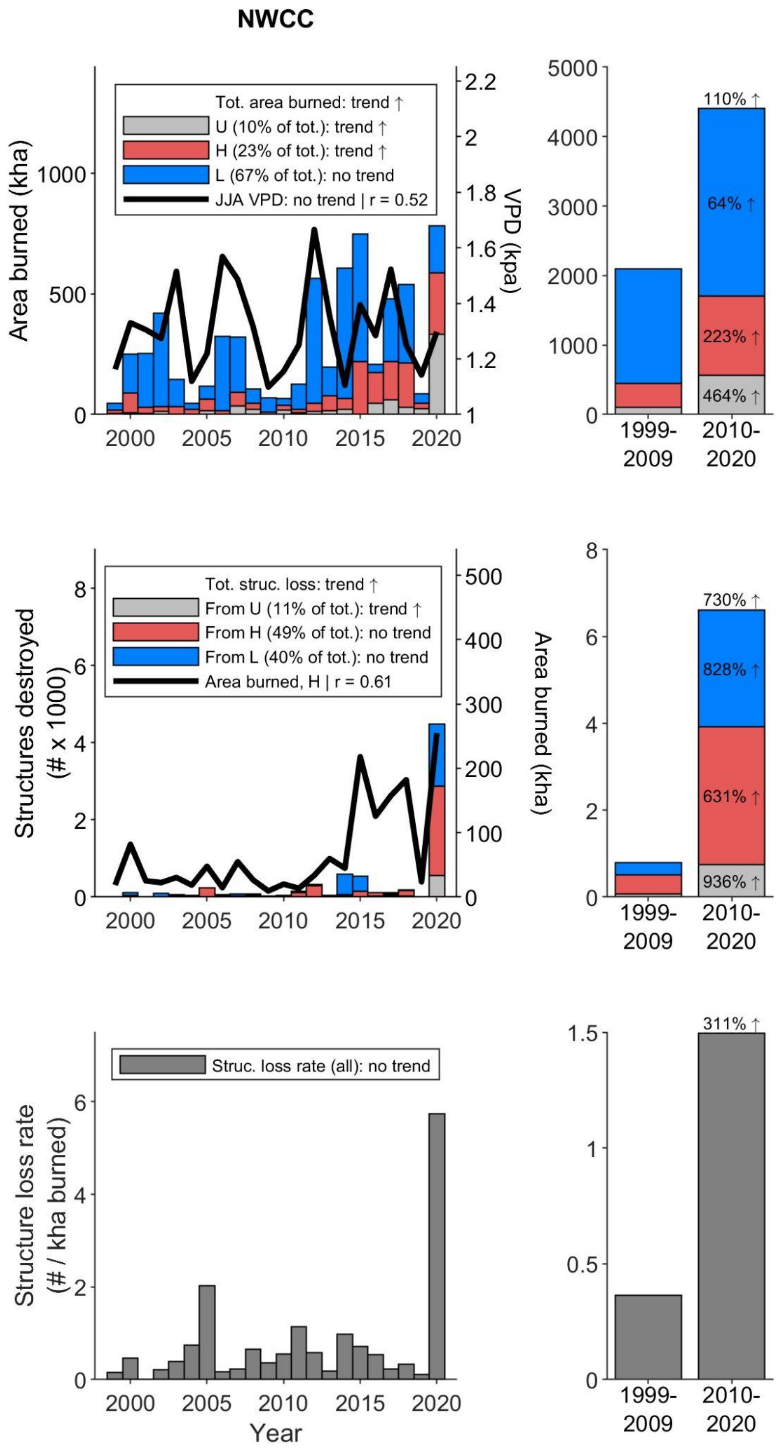
NWCC
Tot. area burned: trend ↑
U (10% of tot.): trend ↑
H (23% of tot.): trend ↑
L (67% of tot.): no trend
JJA VPD: no trend | r = 0.52
Area burned (kha)
VPD (kpa)
110% ↑
64% ↑
223% ↑
464% ↑
Tot. struc. loss: trend ↑
From U (11% of tot.): trend ↑
From H (49% of tot.): no trend
From L (40% of tot.): no trend
Area burned, H | r = 0.61
Structures destroyed (# x 1000)
Area burned (kha)
730% ↑
828% ↑
631% ↑
936% ↑
Struc. loss rate (all): no trend
Structure loss rate (# / kha burned)
311% ↑
Year
1999-2009
2010-2020

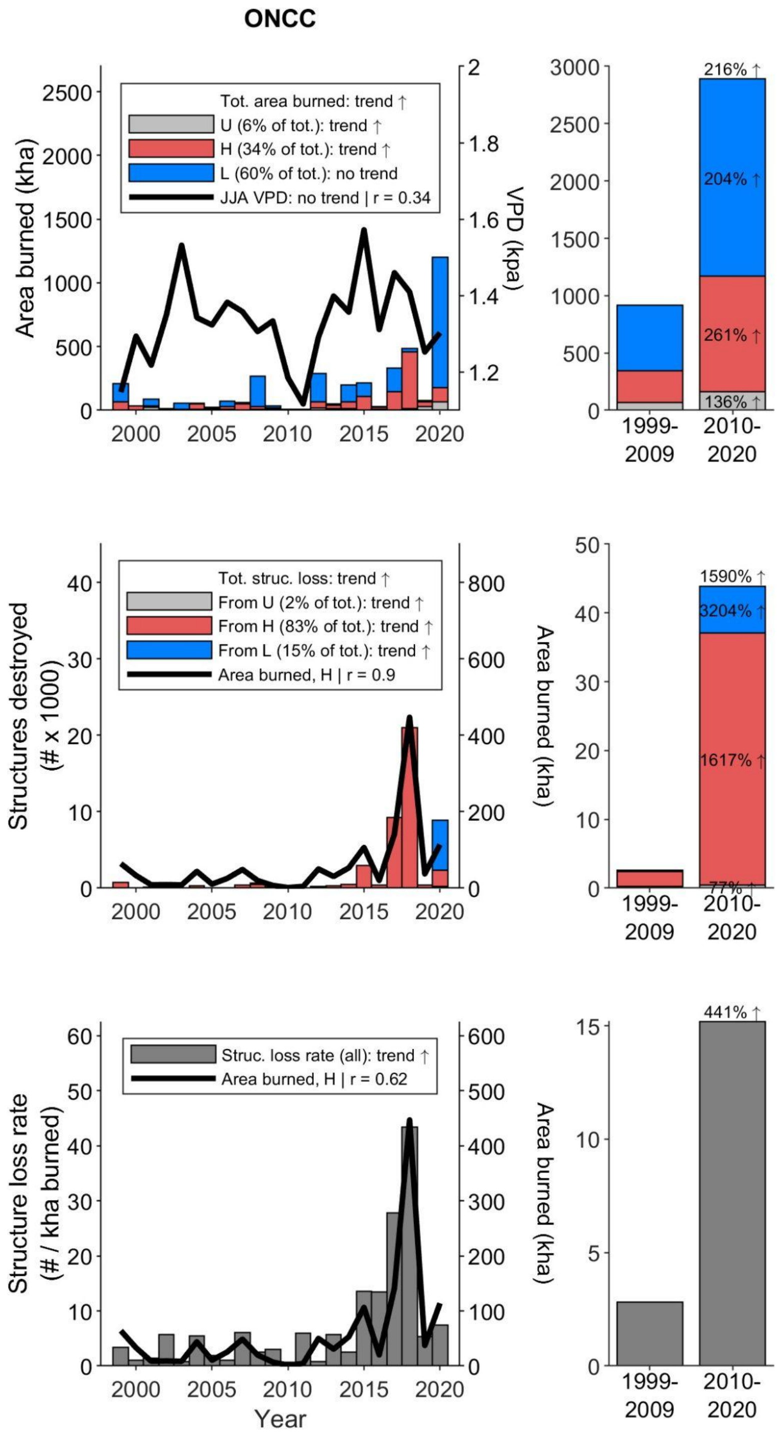
ONCC
Tot. area burned: trend ↑
U (6% of tot.): trend ↑
H (34% of tot.): trend ↑
L (60% of tot.): no trend
JJA VPD: no trend | r = 0.34
Area burned (kha)
VPD (kpa)
216% ↑
204% ↑
261% ↑
136% ↑
Tot. struc. loss: trend ↑
From U (2% of tot.): trend ↑
From H (83% of tot.): trend ↑
From L (15% of tot.): trend ↑
Area burned, H | r = 0.9
Structures destroyed (# x 1000)
Area burned (kha)
1590% ↑
3204% ↑
1617% ↑
77% ↑
Struc. loss rate (all): trend ↑
Area burned, H | r = 0.62
Structure loss rate (# / kha burned)
Area burned (kha)
441% ↑
Year
1999-2009
2010-2020

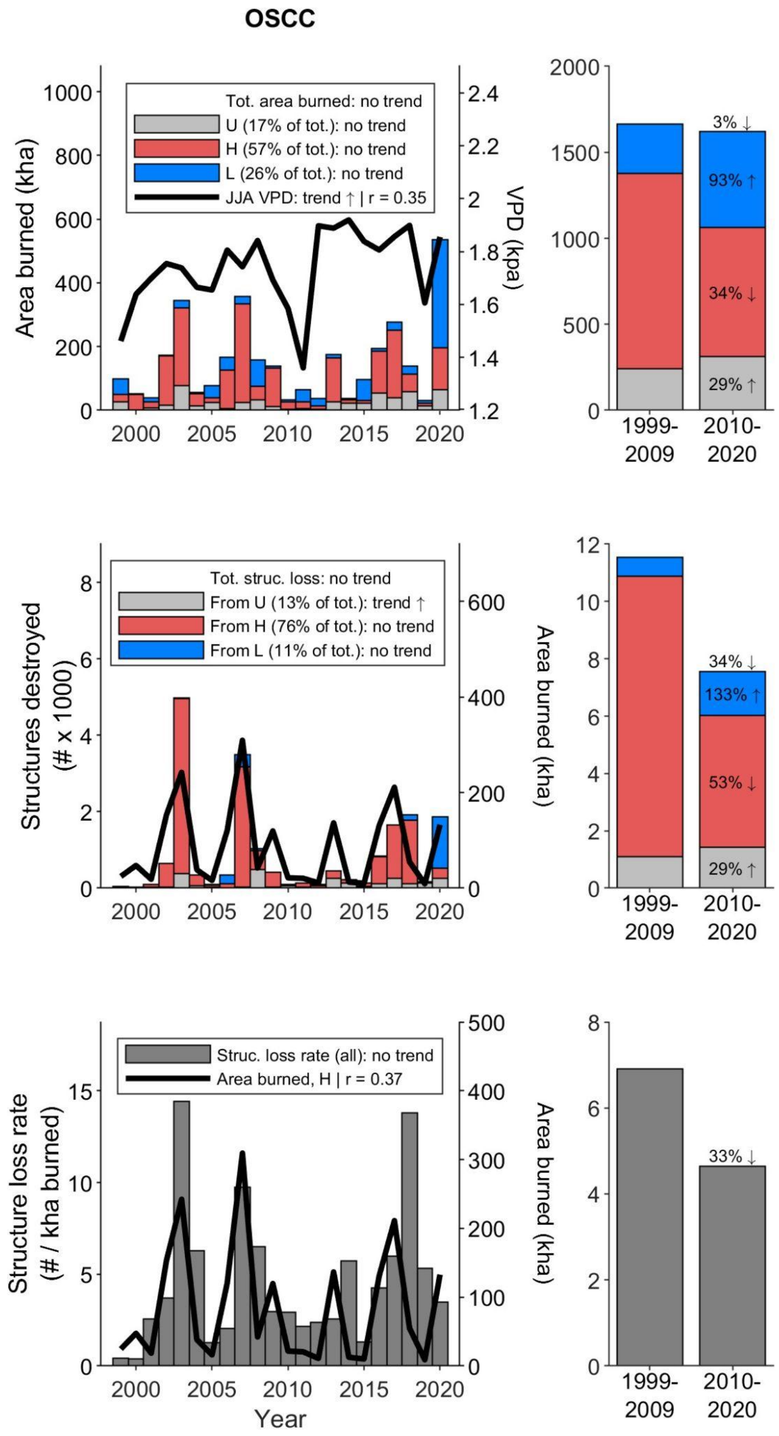
OSCC
Tot. area burned: no trend
U (17% of tot.): no trend
H (57% of tot.): no trend
L (26% of tot.): no trend
JJA VPD: trend ↑ | r = 0.35
Area burned (kha)
VPD (kpa)
3% ↓
93% ↑
34% ↓
29% ↑
1999-2009
2010-2020
Tot. struc. loss: no trend
From U (13% of tot.): trend ↑
From H (76% of tot.): no trend
From L (11% of tot.): no trend
Structures destroyed (# x 1000)
Area burned (kha)
34% ↓
133% ↑
53% ↓
29% ↑
Struc. loss rate (all): no trend
Area burned, H | r = 0.37
Structure loss rate (# / kha burned)
Area burned (kha)
33% ↓
Year

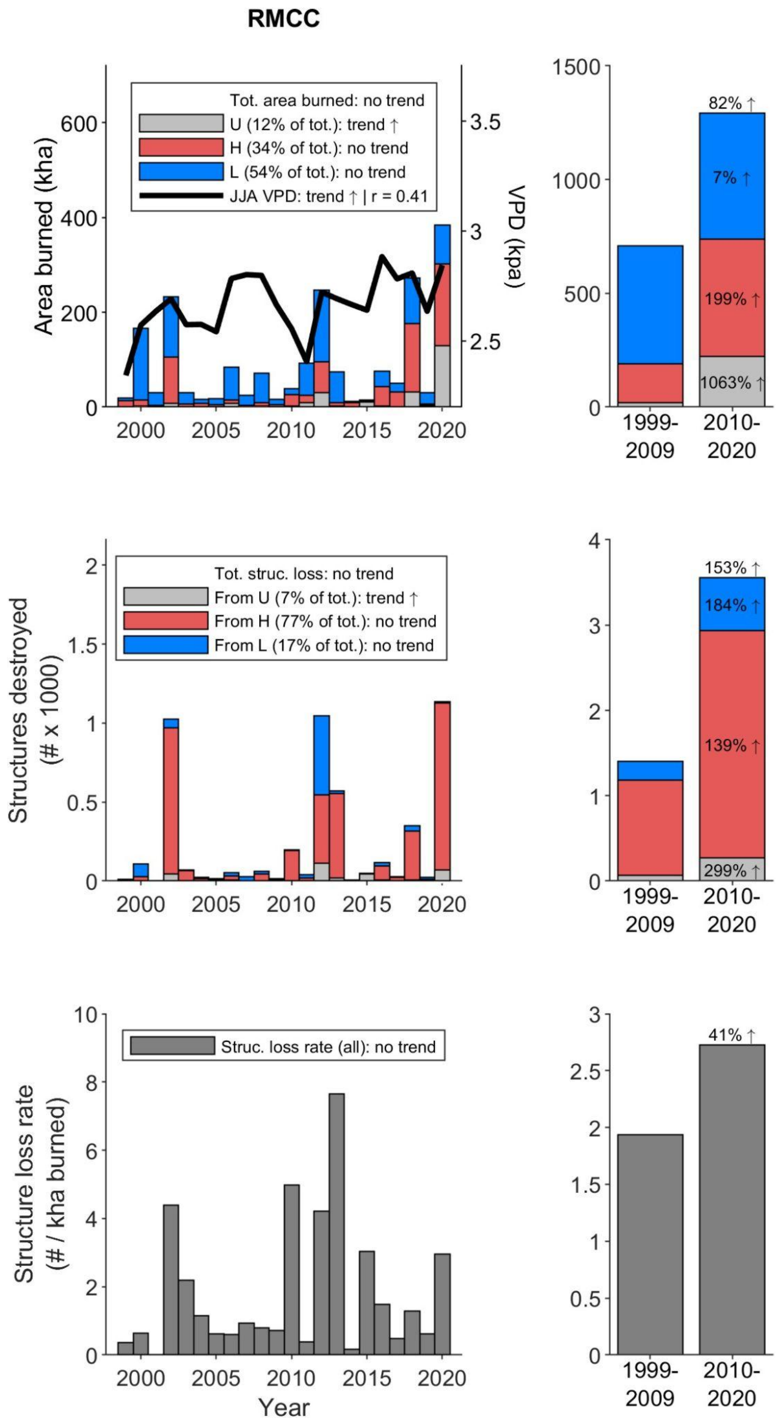
RMCC
Tot. area burned: no trend
U (12% of tot.): trend ↑
H (34% of tot.): no trend
L (54% of tot.): no trend
JJA VPD: trend ↑ | r = 0.41
Area burned (kha)
VPD (kpa)
82% ↑
7% ↑
199% ↑
1063% ↑
1999-2009
2010-2020
Tot. struc. loss: no trend
From U (7% of tot.): trend ↑
From H (77% of tot.): no trend
From L (17% of tot.): no trend
Structures destroyed (# x 1000)
153% ↑
184% ↑
139% ↑
299% ↑
Struc. loss rate (all): no trend
Structure loss rate (# / kha burned)
41% ↑
Year

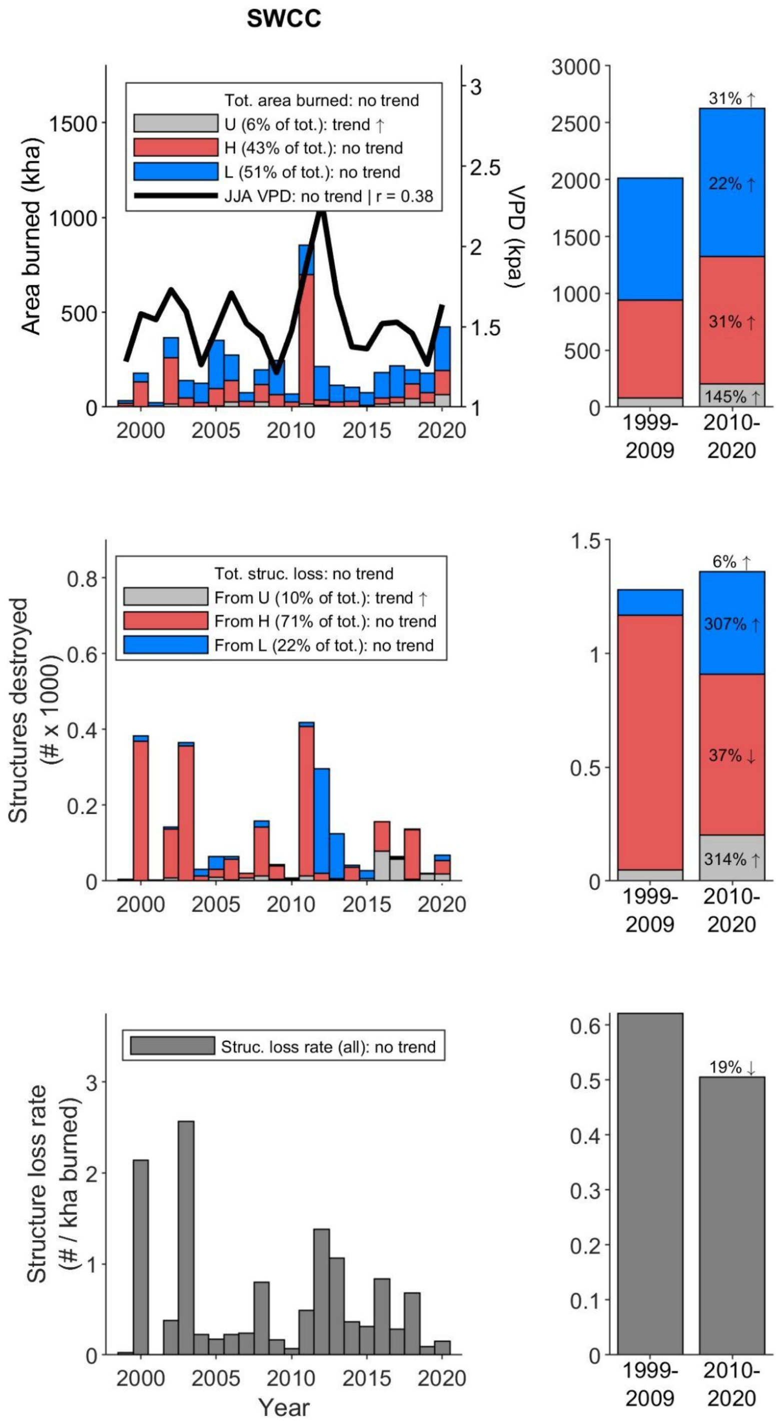
SWCC
Tot. area burned: no trend
U (6% of tot.): trend ↑
H (43% of tot.): no trend
L (51% of tot.): no trend
JJA VPD: no trend | r = 0.38
Area burned (kha)
VPD (kpa)
31% ↑
22% ↑
31% ↑
145% ↑
1999-2009
2010-2020
Tot. struc. loss: no trend
From U (10% of tot.): trend ↑
From H (71% of tot.): no trend
From L (22% of tot.): no trend
Structures destroyed (# x 1000)
6% ↑
307% ↑
37% ↓
314% ↑
Struc. loss rate (all): no trend
Structure loss rate (# / kha burned)
19% ↓
Year

Figure S11. GACC-level fire regime attributes by ignition source for the western United States. As in Figure 3 in the main text; legend is the same for the entire bottom row. Non-significant between-median values are labeled with "Medians not sig. diff." if the Wilcoxon rank-sum tests yielded a p-value > 0.10. Geographic area coordination center (GACC) delineations are visible at https://gacc.nifc.gov/. NOTE: Areas outside of Montana, Wyoming, and Colorado are NOT included in the Northern Rockies and Rocky Mountain GACC summaries.

BELOW

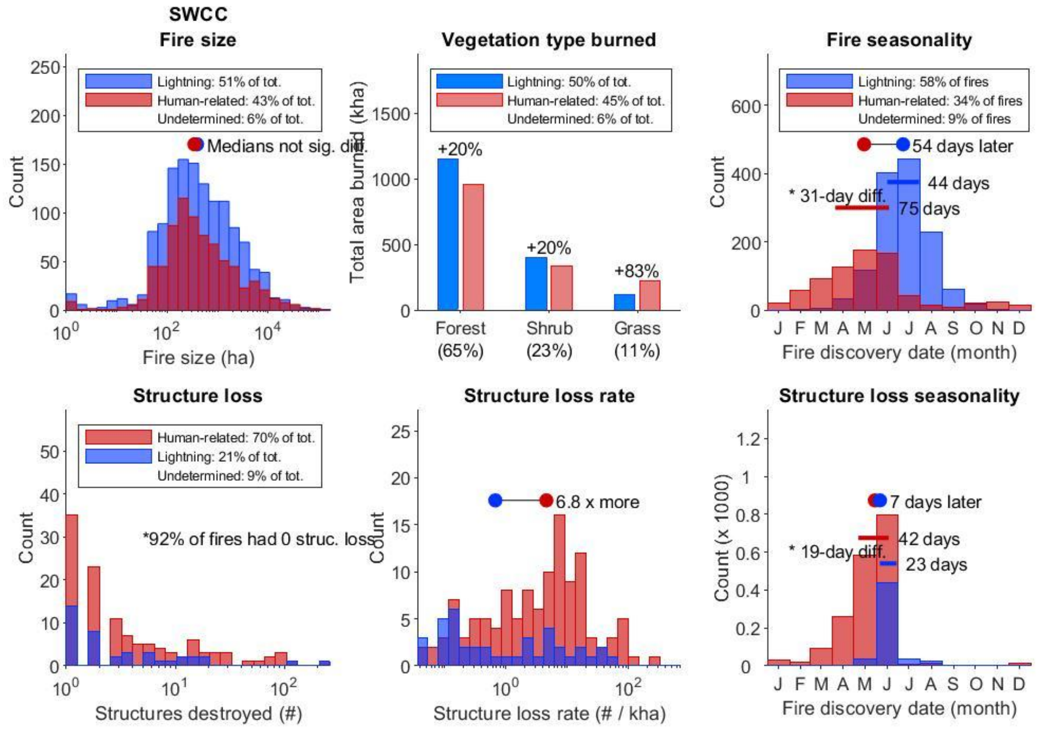

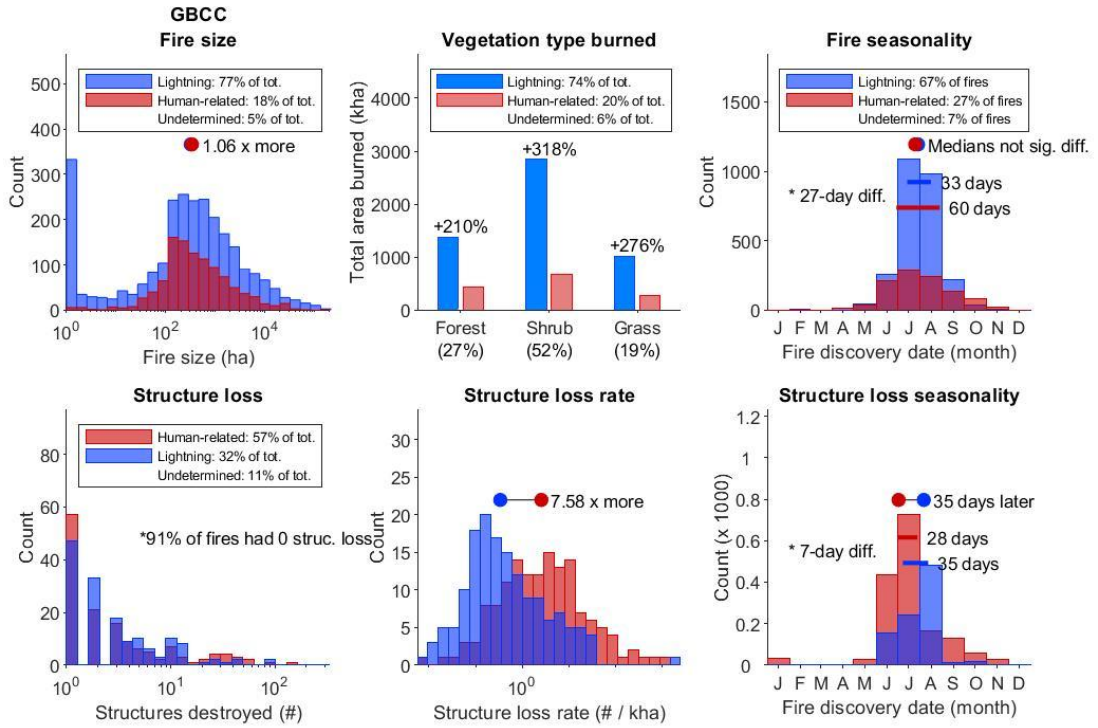
GBCC
Fire size
Lightning: 77% of tot.
Human-related: 18% of tot.
Undetermined: 5% of tot.
1.06 x more
Count
Fire size (ha)
Vegetation type burned
Lightning: 74% of tot.
Human-related: 20% of tot.
Undetermined: 6% of tot.
Total area burned (kha)
+210%
+318%
+276%
Forest (27%)
Shrub (52%)
Grass (19%)
Fire seasonality
Lightning: 67% of fires
Human-related: 27% of fires
Undetermined: 7% of fires
Medians not sig. diff.
* 27-day diff.
33 days
60 days
Fire discovery date (month)
Structure loss
Human-related: 57% of tot.
Lightning: 32% of tot.
Undetermined: 11% of tot.
*91% of fires had 0 struc. loss
Structures destroyed (#)
Structure loss rate
7.58 x more
Structure loss rate (# / kha)
Structure loss seasonality
35 days later
* 7-day diff.
28 days
35 days
Count (x 1000)
Fire discovery date (month)

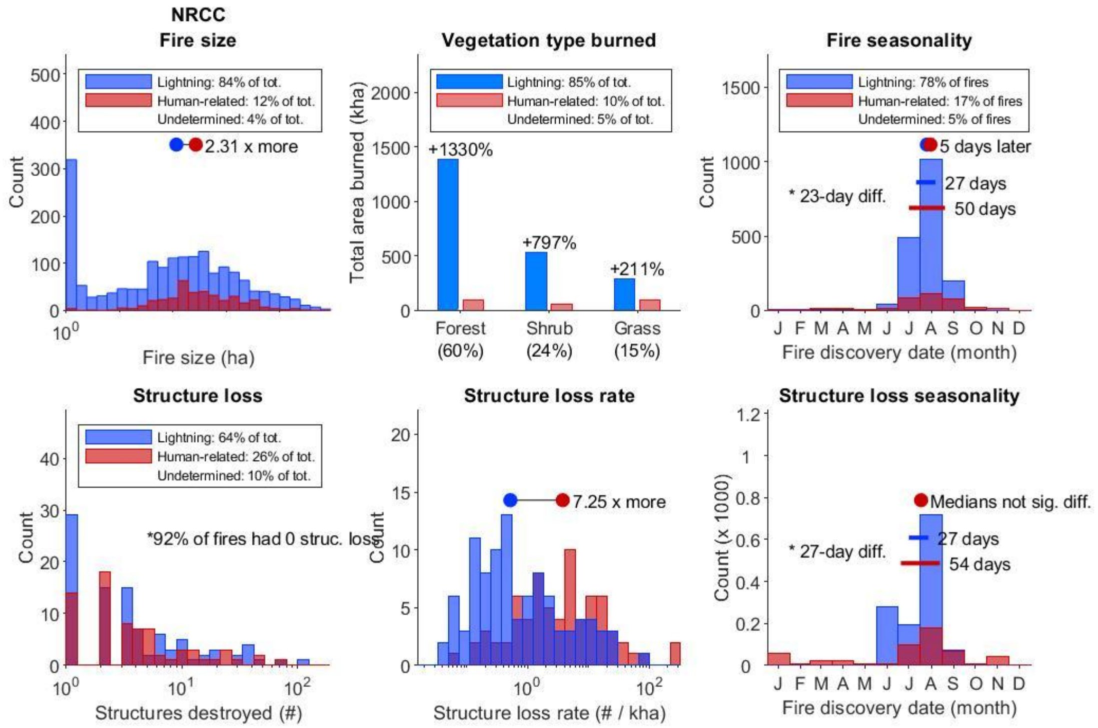
NRCC
Fire size
Lightning: 84% of tot.
Human-related: 12% of tot.
Undetermined: 4% of tot.
2.31 x more
Count
Fire size (ha)
Vegetation type burned
Lightning: 85% of tot.
Human-related: 10% of tot.
Undetermined: 5% of tot.
+1330%
+797%
+211%
Total area burned (kha)
Forest (60%)
Shrub (24%)
Grass (15%)
Fire seasonality
Lightning: 78% of fires
Human-related: 17% of fires
Undetermined: 5% of fires
5 days later
27 days
50 days
* 23-day diff.
Count
J F M A M J J A S O N D
Fire discovery date (month)
Structure loss
Lightning: 64% of tot.
Human-related: 26% of tot.
Undetermined: 10% of tot.
*92% of fires had 0 struc. loss
Count
Structures destroyed (#)
Structure loss rate
7.25 x more
Count
Structure loss rate (# / kha)
Structure loss seasonality
Medians not sig. diff.
27 days
54 days
* 27-day diff.
Count (x 1000)
J F M A M J J A S O N D
Fire discovery date (month)

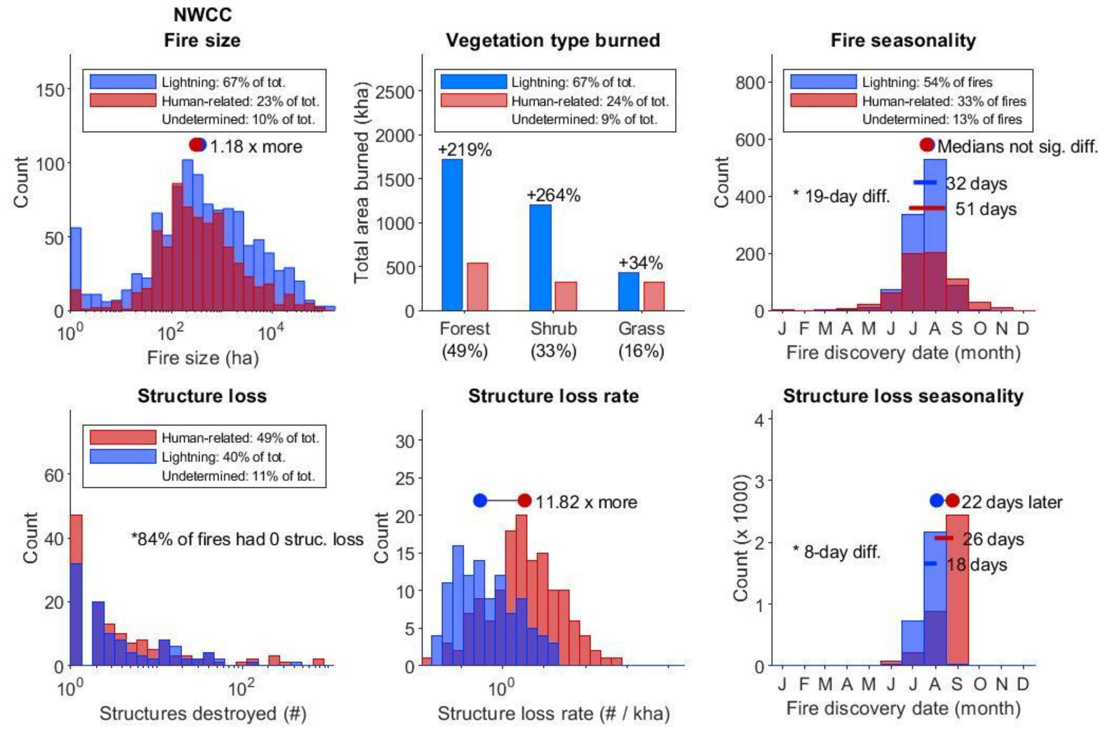
NWCC
Fire size
Lightning: 67% of tot.
Human-related: 23% of tot.
Undetermined: 10% of tot.
1.18 x more
Count
Fire size (ha)
Vegetation type burned
Lightning: 67% of tot.
Human-related: 24% of tot.
Undetermined: 9% of tot.
+219%
+264%
+34%
Total area burned (kha)
Forest (49%)
Shrub (33%)
Grass (16%)
Fire seasonality
Lightning: 54% of fires
Human-related: 33% of fires
Undetermined: 13% of fires
Medians not sig. diff.
* 19-day diff.
32 days
51 days
Count
J F M A M J J A S O N D
Fire discovery date (month)
Structure loss
Human-related: 49% of tot.
Lightning: 40% of tot.
Undetermined: 11% of tot.
*84% of fires had 0 struc. loss
Count
Structures destroyed (#)
Structure loss rate
11.82 x more
Count
Structure loss rate (# / kha)
Structure loss seasonality
22 days later
* 8-day diff.
26 days
18 days
Count (x 1000)
J F M A M J J A S O N D
Fire discovery date (month)

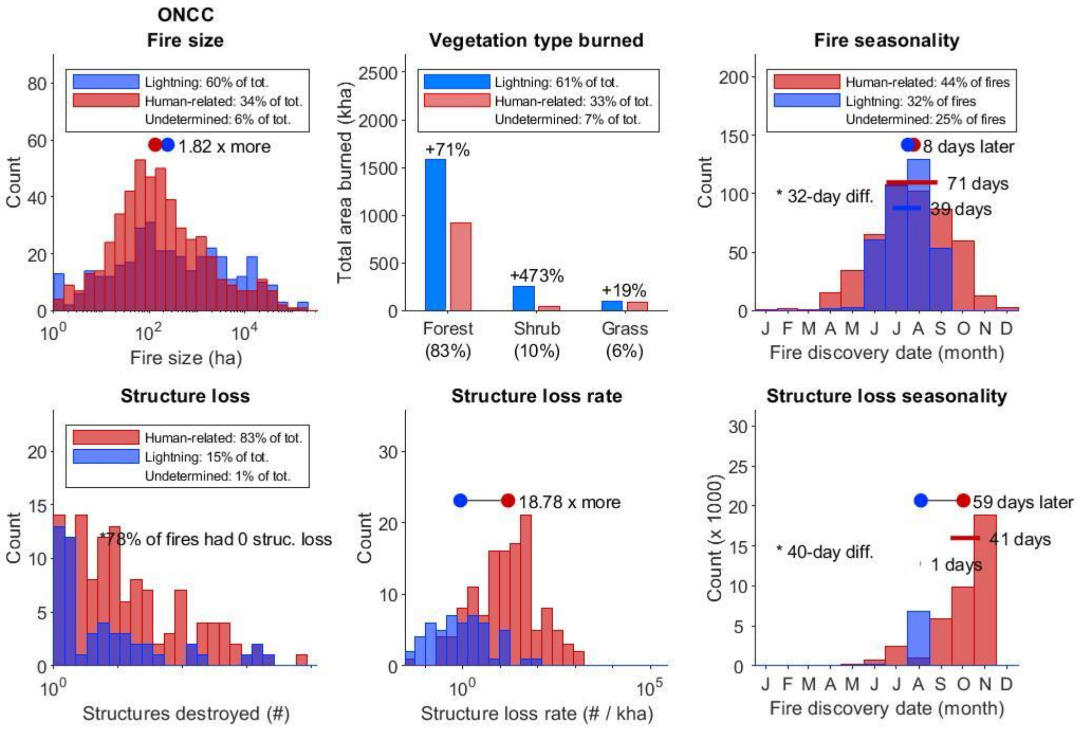
ONCC
Fire size
Lightning: 60% of tot.
Human-related: 34% of tot.
Undetermined: 6% of tot.
1.82 x more
Count
Fire size (ha)
Vegetation type burned
Lightning: 61% of tot.
Human-related: 33% of tot.
Undetermined: 7% of tot.
+71%
+473%
+19%
Total area burned (kha)
Forest (83%)
Shrub (10%)
Grass (6%)
Fire seasonality
Human-related: 44% of fires
Lightning: 32% of fires
Undetermined: 25% of fires
8 days later
71 days
39 days
* 32-day diff.
Count
Fire discovery date (month)
Structure loss
Human-related: 83% of tot.
Lightning: 15% of tot.
Undetermined: 1% of tot.
*78% of fires had 0 struc. loss
Count
Structures destroyed (#)
Structure loss rate
18.78 x more
Count
Structure loss rate (# / kha)
Structure loss seasonality
59 days later
41 days
1 days
* 40-day diff.
Count (x 1000)
Fire discovery date (month)

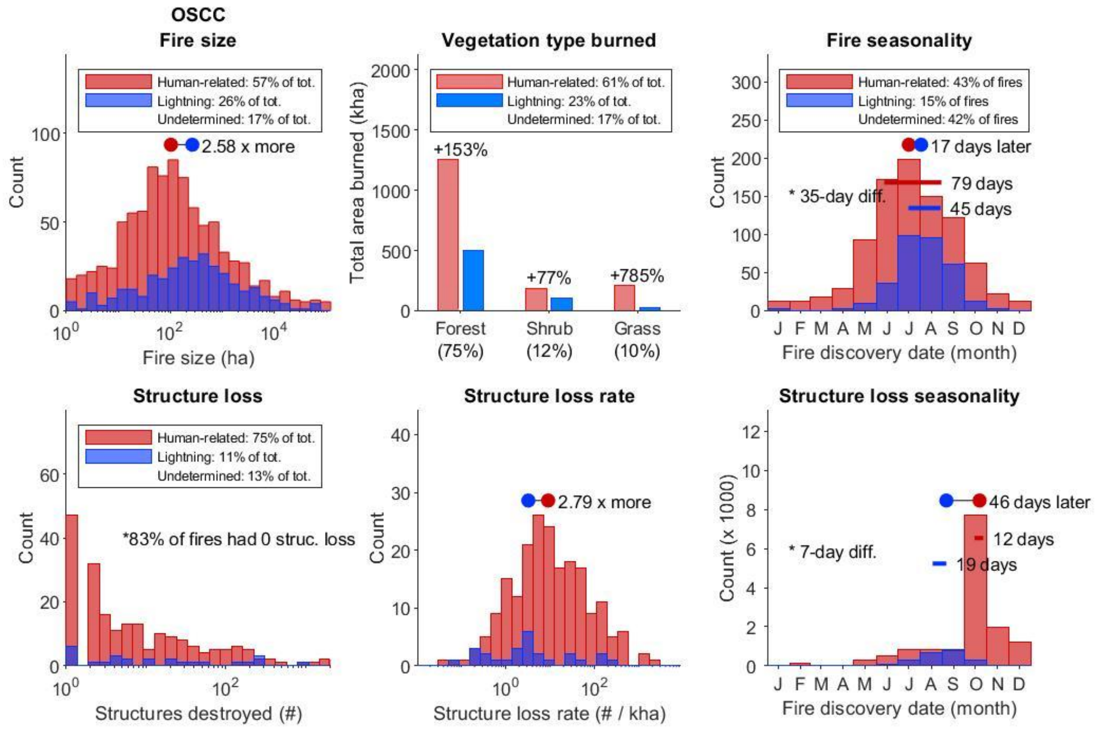
OSCC
Fire size
Human-related: 57% of tot.
Lightning: 26% of tot.
Undetermined: 17% of tot.
2.58 x more
Count
Fire size (ha)
Vegetation type burned
Human-related: 61% of tot.
Lightning: 23% of tot.
Undetermined: 17% of tot.
+153%
+77%
+785%
Total area burned (kha)
Forest (75%)
Shrub (12%)
Grass (10%)
Fire seasonality
Human-related: 43% of fires
Lightning: 15% of fires
Undetermined: 42% of fires
17 days later
79 days
45 days
* 35-day diff.
Fire discovery date (month)
Structure loss
Human-related: 75% of tot.
Lightning: 11% of tot.
Undetermined: 13% of tot.
*83% of fires had 0 struc. loss
Structures destroyed (#)
Structure loss rate
2.79 x more
Structure loss rate (# / kha)
Structure loss seasonality
46 days later
12 days
19 days
* 7-day diff.
Count (x 1000)
Fire discovery date (month)

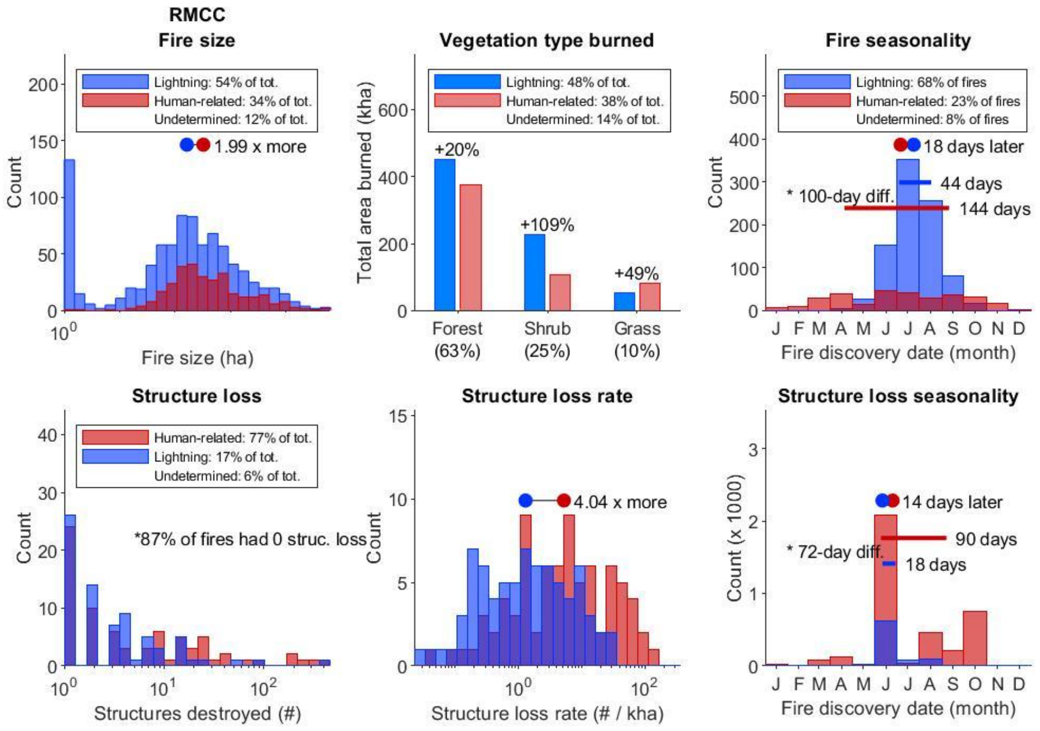
RMCC
Fire size
Lightning: 54% of tot.
Human-related: 34% of tot.
Undetermined: 12% of tot.
1.99 x more
Count
Fire size (ha)
Vegetation type burned
Lightning: 48% of tot.
Human-related: 38% of tot.
Undetermined: 14% of tot.
Total area burned (kha)
+20%
+109%
+49%
Forest (63%)
Shrub (25%)
Grass (10%)
Fire seasonality
Lightning: 68% of fires
Human-related: 23% of fires
Undetermined: 8% of fires
18 days later
44 days
* 100-day diff.
144 days
Fire discovery date (month)
Structure loss
Human-related: 77% of tot.
Lightning: 17% of tot.
Undetermined: 6% of tot.
*87% of fires had 0 struc. loss
Structures destroyed (#)
Structure loss rate
4.04 x more
Structure loss rate (# / kha)
Structure loss seasonality
14 days later
90 days
* 72-day diff.
18 days
Count (x 1000)
Fire discovery date (month)

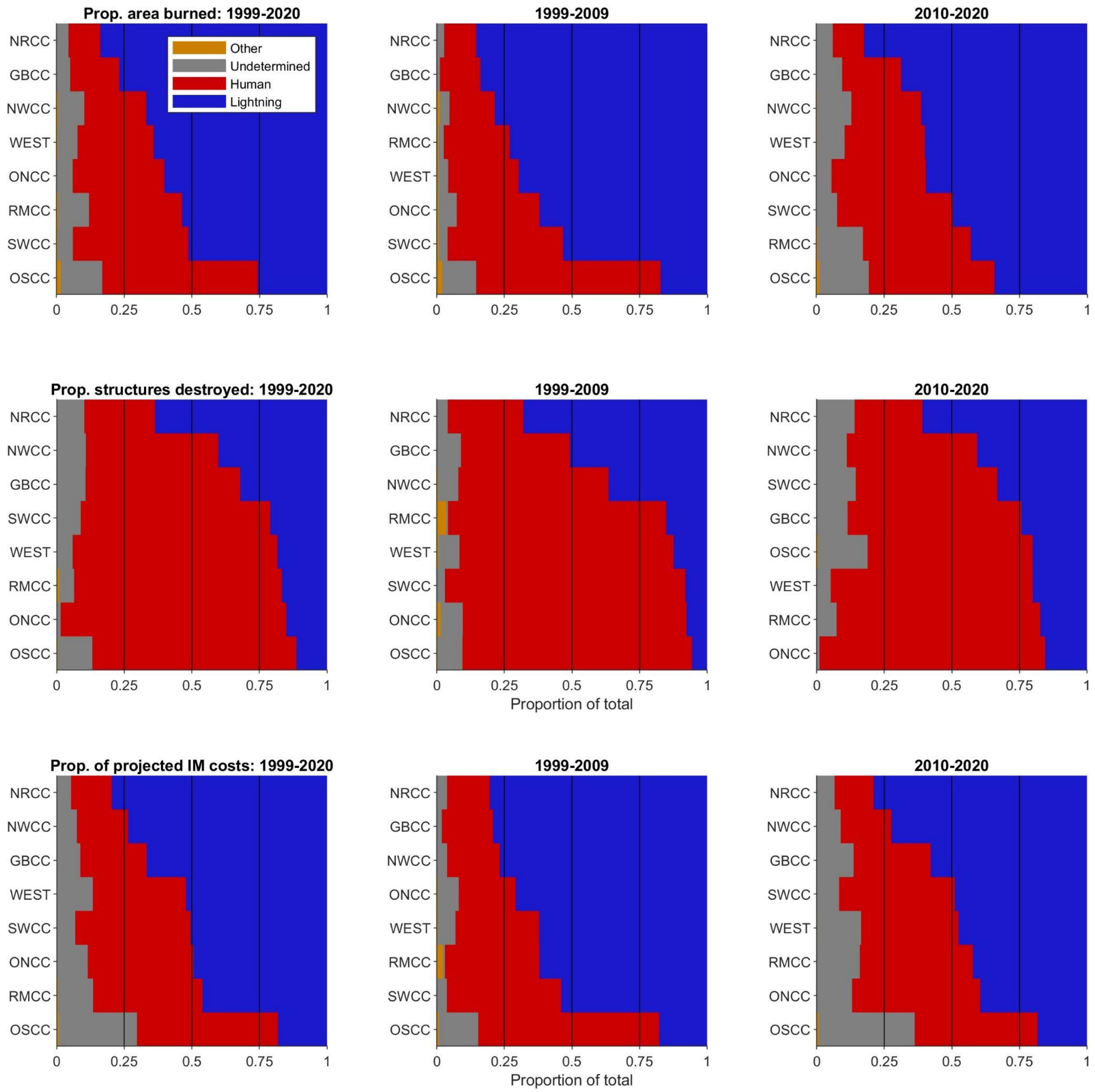

Fig. S12. GACC-level proportion of total area burned, structure loss, and incident-management costs by ignition classification in the raw dataset, by time period. Within each panel, states are ordered from highest to lowest based on the contribution from lightning-ignited fires, including the West-wide value ("WEST"). Projected incident-management (IM) costs do not reflect final costs of each fire event, but are rather the estimated costs at the closing of the incident management phase.
